# A sting affair: A global quantitative exploration of bee, wasp and ant hosts of velvet ants

**DOI:** 10.1371/journal.pone.0238888

**Published:** 2020-09-11

**Authors:** Federico Ronchetti, Carlo Polidori

**Affiliations:** 1 Department of Animal Ecology and Tropical Biology, University of Wuerzburg, Würzburg, Germany; 2 Instituto de Ciencias Ambientales (ICAM), Universidad de Castilla-La Mancha, Toledo, Spain; Free University of Bozen-Bolzano, ITALY

## Abstract

The vast majority of species of velvet ants (Hymenoptera: Aculeata: Mutillidae) are ectoparasitoids of immature stages of other aculeate Hymenoptera (bees, wasps and ants). Due to their cryptic, furtive behaviour at the host nesting sites, however, even basic information on their biology, like host use diversity, is still unknown for entire subfamilies, and the known information, scattered in over two centuries of published studies, is potentially hiding tendencies to host specialization across velvet ant lineages. In this review, based on 305 host associations spanning 132 species in 49 genera and 10 main lineages (tribes/subfamilies), we explored patterns of host use in velvet ants. Overall, 15 families and 29 subfamilies of Aculeata are listed as hosts of mutillids, with a strong predominance of Apoidea (bees and apoid wasps: 19 subfamilies and 82.3% of host records). A series of bipartite networks, multivariate analyses and calculations of different indices suggested possible patterns of specialization. Host taxonomic spectrum (number of subfamilies) of velvet ants was very variable and explained by variation in the number of host records. Instead, we found a great variation of network-based host specialization degree and host taxonomic distinctness that did not depend on the number of host records. Differences in host use patterns seemed apparent across mutillid tribes/subfamilies, among genera within several tribes/subfamilies, and to lesser extent within genera. Taxonomic host use variation seemed not dependent on phylogeny. Instead, it was likely driven by the exploitation of hosts with different ecological traits (nest type, larval diet and sociality). Thus, taxonomically more generalist lineages may use hosts that essentially share the same ecological profile. Interestingly, closely related mutillid lineages often show contrasting combinations of host ecological traits, particularly sociality and larval diet, with a more common preference for ground-nesting hosts across most lineages. This review may serve as a basis to test hypotheses for host use evolution in this fascinating family of parasitoids.

## Introduction

**Fig 1:**
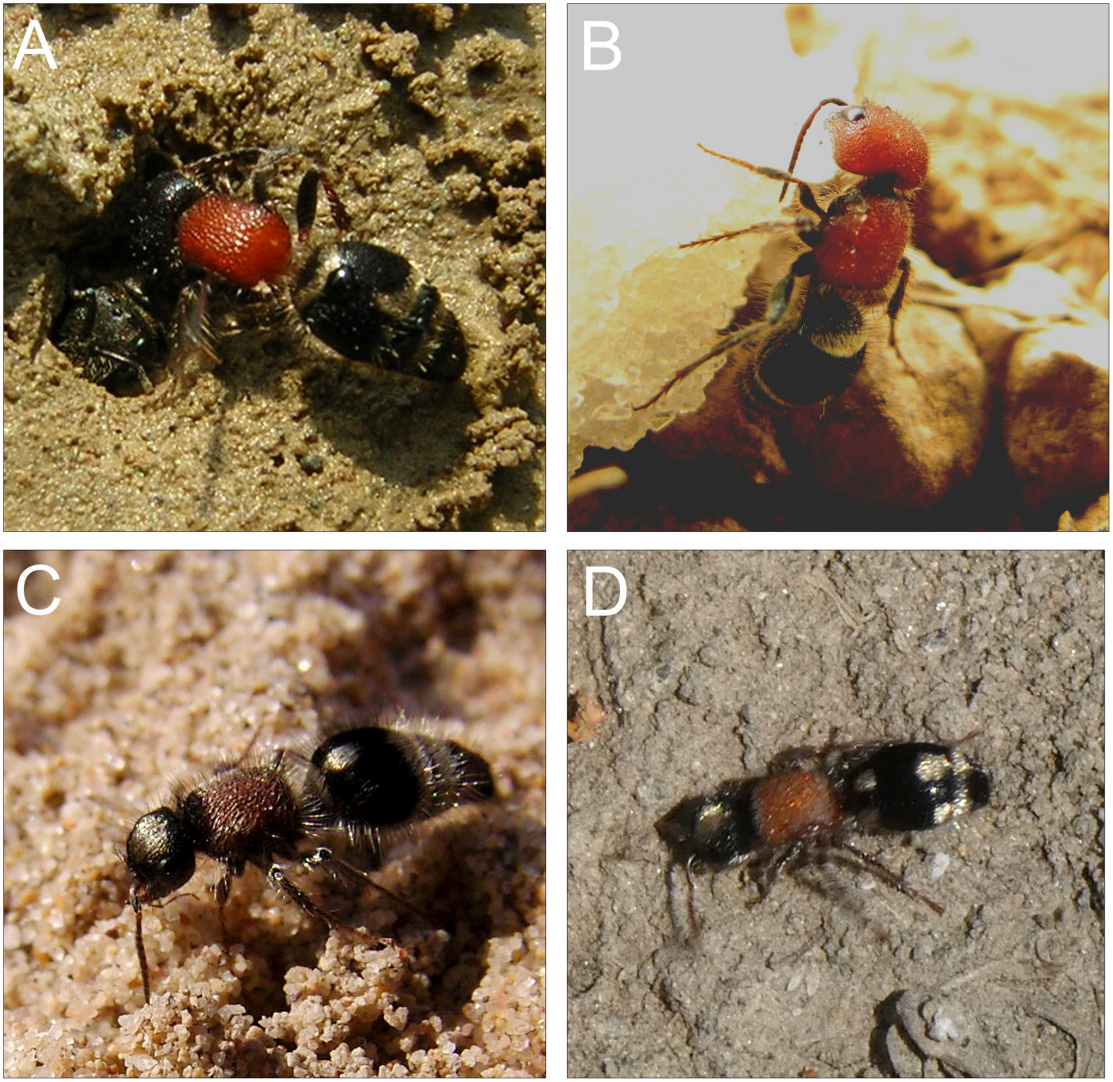
Pictures of representative species of velvet ants. **A**, *Myrmilla capitata* (Myrmillinae) at the nest entrance of its *Lasioglossum* host (note the bee head in the nest). **B**. *Myrmilla erythrocephala* (Myrmillinae). **C**, *Nemka viduata* (Smicromyrmini). **D**, *Ronisia ghilliani* (Mutillini).

The large wasp family Mutillidae (Hymenoptera: Aculeata) includes 4603 valid species in 220 genera [1, 2]. Their common name, velvet ants, stems from the well-visible and often dense pilosity all over their body as well as from the wingless nature of females (males are, except few exceptions, winged), which remind worker ants [3–5] (Fig 1). Velvet ants show an interesting bouquet of defensive strategies [6] that recently deserved them the name of “indestructible insects” [7], after having proved the largely unsuccessful predation by vertebrates. Almost all of species studied to date have the ability to stridulate by rubbing a scraper on the gaster tergite II against a file on gaster tergite III [8–10]. While this acoustical behaviour was associated with mating in several species [11, 12], stridulation is believed to primarily serve as deterrent to the attack of predators [13, 14]. Mutillids also express different patterns of aposematic coloration, building up in some areas large Müllerian mimicry complexes [15, 16]. Females possess the longest sting compared to their body size among aculeate Hymenoptera (stinging wasps, bees and ants) [17], have a remarkably strong exoskeleton [13], Zn-enriched mandibles [18], and are heavily poisonous [19, 20].

Despite such peculiar traits which make them unique among aculeate Hymenoptera, biological information on velvet ants are still scarce and fragmentary after more than two centuries of investigations [3, 21–25]. This is certainly due to their shy, cryptic behaviour in the field, which limit extensive observations [26], to the difficulty to rear them in the laboratory, and to the fact that most information on their behaviour and ecology actually comes as side results in studied devoted to the biology of their hosts (see S1 Table).

What we known on the biology of velvet ants is essentially that they are ectoparasitoids (natural enemies attacking one victim during a life-stage, eliminating its fitness [27]) of developmental stages of other insects. Despite several hosts were reported from Diptera [28, 29], Coleoptera [30], Lepidoptera [31], and Blattodea [32], the vast majority of hosts belong to other aculeate Hymenoptera [3, 21,22,33]. Female mutillids normally attack the post-defecated larvae or pupae of their hosts, and especially parasitize species which enclose the offspring in concealed places such as brood cells within nests or buried or exposed oothecae and cocoons [21, 34]. Females of species attacking bees and wasps are often observed patrolling the soil patches where high densities of host nests occur [35–39], especially in the early morning and late afternoon, perhaps to avoid walking on very hot surface during the central hours of summer days [38–40]. Some species are nocturnal [41, 42].

Almost 170 years after the first clear record of parasitoidism from an aculeate host [43, 44], it is estimated that hosts are known for only 2%-3% of all described species of Mutillidae [21, 45]. Host records are sparse and widely scattered across a huge amount of papers and books, and to date the only attempts to review such information consist in the works of Brothers et al. [22] and Luz et al. [33]. These reviews help exploring host diversity across mutillid lineages but with important biases, since the former study only concerns mutillids that attack social Hymenoptera and the latter study only concerns mutillids attacking bees in the Neotropics. No efforts were carried out to date to summarize and explore the host associations known for velvet ants globally.

The purpose of our study is to fill this gap through a global revision of the literature, by presenting and quantitatively exploring hymenopteran host use in Mutillidae. We decided to focus on hymenopteran hosts because they represent the vast majority of known hosts, and because restricting to such host group may allow identifying scenarios of host use patterns within a single, albeit rich and diverse, insect group which also includes mutillids themselves [46]. The scattered distribution of biological data on velvet ants makes unclear if there is a widespread potential for host polyphagy, which would be in general agreement with what predicted for hymenopteran parasitoids [47, 48]. As a matter of fact, many species of bees and wasps from most of known families and several species of ants are currently listed as hosts of velvet ants [3, 21,22,33]. However, different clades of velvet ants may tend to specialize to different hosts, as it was seen in other aculeate parasitoids of Aculeata such as Chrysididae [49]. The lack of quantitative, global studies may be hiding such tendencies to specialization across velvet ant taxonomic levels. Such specialization may be driven by taxonomic biases in host use as well as by variation in ecological traits of the hosts, according to the general hypothesis that species evolution toward true generalism is unlikely, given that specialization lowers the competition for resources [50]. Thus, in the present study, we created a large dataset of the hymenopteran hosts of velvet ants and quantitatively explored patterns in host use from both a taxonomic and an ecological point of view.

## Materials and methods

### Literature survey and extracted data

We retrieved information on host associations in velvet ants from a total of 217 articles/books (S1 Table). Initially, a series of queries in Web of Science were performed by using the key words: Mutillidae, velvet ant, biology, behavior, ecology, host. To the articles found in such a way, we added important books on ecology and behavior of different groups of Hymenoptera that are known to include hosts of mutillids (S1 Table). Some host associations reported in old works were retrieved from more recent works citing those studies. Overall, the literature survey rewarded a total of 433 confirmed or potential host associations across 187 species of velvet ants. We cannot exclude that some old host records were missed. However, our total sample was large and we feel it was reasonably adequate to depict patterns of host use.

We considered as confirmed host associations those proved by emergence of mutillid individuals from the host immatures and/or from the host nests, and those cases in which adult mutillid females were discovered while inside the host nests. Rarely, mutillids act as kleptoparasites rather than parasitoids of other hymenopterans, and these associations were also included. Potential host records refer to a diverse range of situations suggesting parasitoidism, such as observations of adult mutillid females at potential host nest aggregations but not found/not checked into the host nests [e.g. 51–54]. One new host association was also added, after a field work in Central Italy (Alberese, Grosseto Province) in July 2010, during which we observed two females of *Myrmilla erythrocephala* by digging through a nest entrance (1 case) and inside a brood cell of a nest (1 case) of the ground-nesting bee *Halictus scabiosae*. At a second nesting site in the same area, the emergence of three females and one male from a nest was observed in July 2020, further confirming this host association. After having applied such criteria, we obtained a total of 305 confirmed host associations and 128 potential host associations (S1 Table). The confirmed host associations regarded 132 species of velvet ants in 49 genera, 9 tribes and 5 subfamilies (S1 Table). The confirmed host associations come from 6 continents, mostly from North America and Europe, which roughly sum up 2/3 of all records, while Africa was the continent with less data (10 records). Less than 50 host records each come from Asia, Australia and South America. The distribution of host records for each mutillid tribe/subfamily differs among continent: for example, Sphaeropthalmini were much more studied in North America, while Smicromyrmini were more heavily investigated in Europe and Asia. Dasymutillini were the only tribe studied in Australia.

Once all data were retrieved, we checked for correct names of all taxa. For Mutillidae, we used the most recent classification provided in [1] and the worldwide updated list of species provided in [2]. The highest taxonomic level used for the quantitative analysis was the tribe, the lowest level was the species. In this work, species-group complexes were treated as a species, and subspecies were not considered. Since Myrmillinae does not include recognized tribes, in the following text we will refer to tribe/subfamily for this level of analysis. For the hosts, we checked the species names by using a diverse bulk of works, mainly [55] for bees and [56] for apoid wasps, *plus* recent papers and websites focusing on taxonomy and/or checklists of certain genera, tribe or subfamilies for the other host groups. Classification of host species follows the most recent molecular phylogenetic studies as well as currently accepted classifications based on morphology [46, 56, 57].

To each host species, we assigned binary values representing three main ecological traits. These were larval diet (carnivorous or herbivorous), nest type (dug in the ground or built as aerial, above-ground structure) and sociality (solitary or social, the latter, in its broadest definition, including eusocial, semisocial and communal organizations). We scored as 0 the states which are believed to be ancestral in the Aculeata (carnivorous larvae, ground-nesting and solitary) and as 1 the states supposedly derived (herbivorous larvae, aerial-nesting and social) [46]. Several hosts of mutillids are parasitoids (e.g. Sapygidae, Scoliidae) and thus do not build a nest. In these cases, nest type refers to the concealment type of the hosts of these parasitoids. Ecological data of hosts were retrieved from relevant literature [55, 58–63].

We used only the 305 confirmed associations in all subsequent analyses, while we briefly discussed in the Discussion section the possible occurrence and diversity of further hosts that at the moment are only potential and need verification.

### Data analysis

In the present work we define our data analysis as explorative, since formal tests related with the diversification of host use cannot be done using such a sample, which spanned data from different sources, different geographic areas, highly variable sample size among taxa and areas, and lacking an ancestral state reconstruction of host use in the family. Our analysis is intended to summarize in an organized way our knowledge of hosts attacked by velvet ants and to explore how different mutillid taxa differ in their host use, with the purpose to present a base on which building future evolutionary studies.

We performed the data analyses at three levels: mutillid tribe/subtribe, mutillid genus and mutillid species. This type of hierarchical analyses, in which host associations are quantitatively explored within progressively lower taxonomic levels, rather than presenting a single analysis in which all mutillid species are considered at once, allows an easier detection of possible patterns in host use. For each level of analysis, we performed two types of quantitative explorations.

First, we attempted to draw patterns in the taxonomic host use. To this purpose, we built a number of interaction matrices in which columns are the mutillid taxa (tribes/subfamilies within the family, genera within each tribe/subfamily, species within each genus) and rows are the host subfamilies. We chose to limit our investigation using this taxonomic level for hosts because very rarely mutillid species had more than one associated host record (S1 Table). Instead, using host subfamily allowed grouping a sufficient number of host records in the matrix cells to perform meaningful calculations. However, we also reported a summary of all the genera listed as hosts of the taxa examined in the present study. The cells in the matrices were populated with interaction frequencies, i.e. the numbers of host records for a particular mutillid taxon retrieved for each of its host subfamily.

We visualized the webs of interactions by pooling the data as bipartite networks in which mutillid taxa were linked to host subfamilies by lines of varying thickness, which represent interaction frequency [64]. Nodes in the webs (taxa) have widths that are proportional to the sum of interactions involving them. Several indices were calculated from these networks [65]. The standardized index *H*_*2*_*’* [66] characterizes the degree of complementary specialization, i.e. host subfamilies’ partitioning; the index ranges from 0 for the most generalized to 1 for the most specialized case. The mean number of shared partners, which is based on the distance matrix between taxa, counts the number of taxa in the other level that both interact with [67, 68]; in our case this is the mean number of host subfamilies shared by any two mutillid taxa. The Horn’s index [69] represents the mean similarity in interaction pattern between taxa of that level, and ranges from 0 (no common use of host subfamilies) to 1 (perfect host niche overlap). The package ‘bipartite’ [65] of the R software [70] has been used to obtain the graphs and calculate the indices.

We explored the relationships among the mutillid taxa based on Bray-Curtis similarity in their host use (i.e. matrices of abundance data of host records for each host subfamily) through a series of cluster analyses, using the UPGMA (Unweighted Pair Group Method with Arithmetic Mean) [71]. The Bray-Curtis similarity index can take values between 0 and 1, with 1 indicating complete overlap in host range and 0 no overlap in host range. The cluster analyses and the production of the associated dendrograms were performed in the software PAST 3.04 [72].

In each level of analysis and for each mutillid taxon, we also calculated the host average taxonomic distinctness (Δ^+^), which is a univariate measure that uses information derived from a hierarchical taxonomic tree to estimate diversity. Δ^+^ is indeed the average path length between all pairs of species through a Linnaean taxonomic tree, thus being a measure of pure taxonomic relatedness [73]. Furthermore, we calculated the variation in taxonomic distinctness (Λ^+^), which represents the variance of these pairwise path lengths and reflects the unevenness of the taxonomic tree, hence being useful to detect those mutillid taxa that use different (unrelated) hosts in a more uneven pattern [74]. Thus, Λ^+^ would be large for a sample that contained clusters not necessarily closely related (contributing long path-lengths) of closely related host species (contributing short path‐lengths). We entered the host taxonomic information on five levels: species, genus, subfamily, family and superfamily. Values of Δ^+^ and Λ^+^ were calculated in the software PRIME 7 [75].

The bipartite graphs, the network indices, the cluster analyses, the indices of taxonomic diversity, and the correlation tests were all produced, at each level of analysis, only for mutillid taxa with ≥ 10 host records (i.e. for matrices with n ≥ 10) to achieve reasonably meaningful results.

Second, we attempted to draw patterns in the ecology-based host use. We visualized all the combinations of the binary states for each of the host three ecological traits at the tribe/subfamily level with matrix plots. The matrix plots show all the individual host records for each mutillid tribe/subfamily, with each line in the plot corresponding to a given host record, represented by the combination of two colours, one linked with ancestral (carnivorous, ground, solitary) and the other linked with derived (herbivorous larvae, aerial, social) host states. We calculated the % of the records with host ancestral states over the total number of records for tribes/subfamilies, of genera within each tribe/subfamily and of species within each genus, and we visualized the results in radial (triangle) plots to help the identification of possible patterns in host ecology variation among mutillid taxa. In each triangular plot, each apex identifies one of the three traits, so that the extant and position of the filled areas in the triangle represent a given combination of % for these traits and allow a rapid identification of host ecology-based use. The plots to visualize host ecology use were produced for all the lower-level taxa within the higher-level taxa used in the previous host taxonomy-analysis (i.e. taxa with ≥ 10 host records).

**Fig 2:**
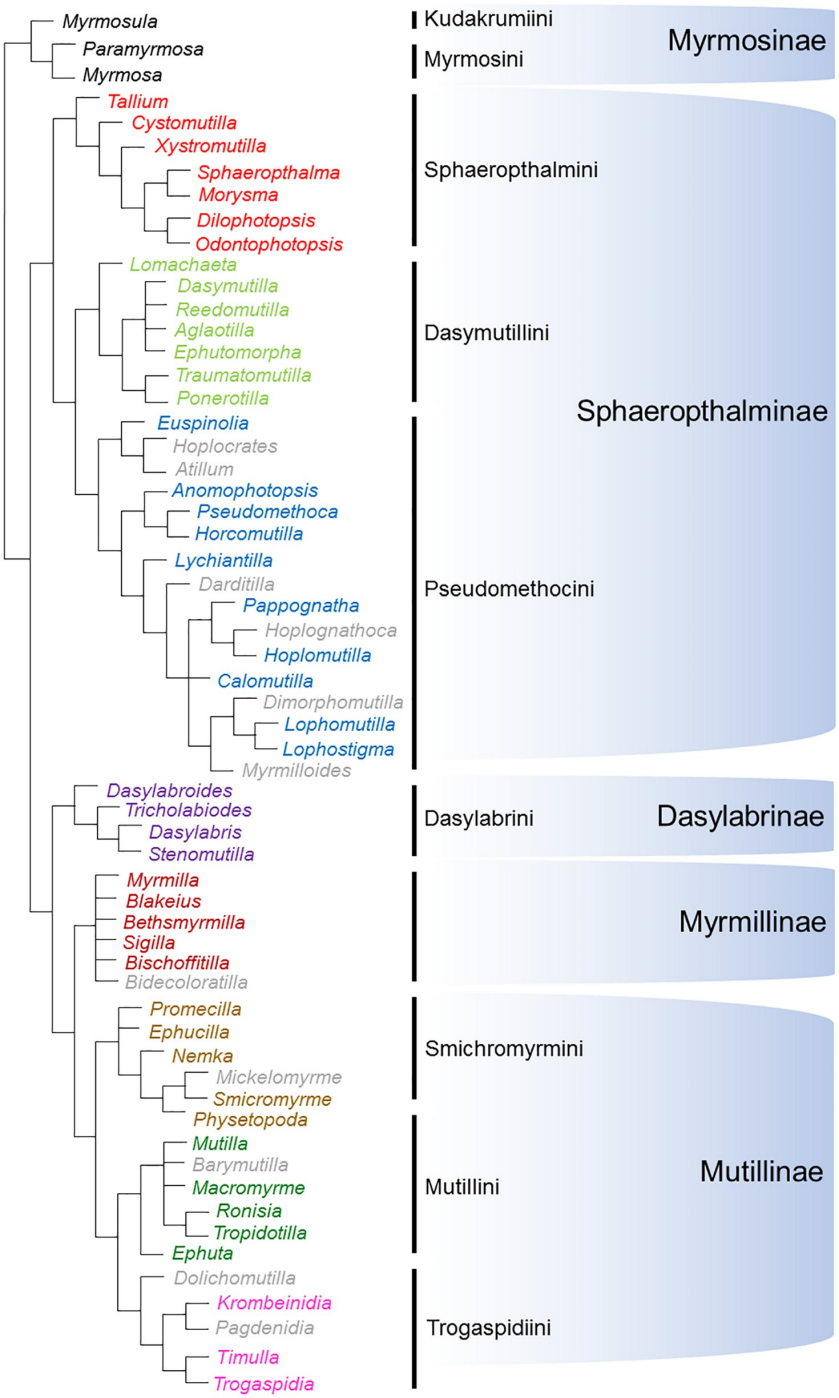
Phylogenetic relationships among the mutillid genera for which we retrieved information from the literature. The hand-made tree follows the most recent phylogenetic hypothesis of [1], based on morphological characters. Differently coloured names indicate the different tribe/subfamilies to which the genera used in the analysis (i.e. confirmed host associations) belong, while names in grey represent genera for which we found potential host associations (not used in the analyses).

We explored any possible connection of patterns of host taxonomic and ecology-based use with phylogenetic relationships between mutillid taxa by hand-drawing a tree based on the most recent morphological phylogeny [1] (Fig 2) and comparing it with bipartite networks, cluster analyses’ dendrograms and host ecology plots.

We carried out Spearman’s correlation tests to verify if the considered measures of host spectrum width (number of host subfamilies), diversity (Δ^+^, Λ^+^) and network-based specialization (*H*_*2*_*’*, mean number of shared hosts, Horn’s index) increase by increasing the sample size (i.e. the number of host records) across all the levels of analysis. Significant positive relationships would suggest that host use patterns are dependent on sample size, so that any conclusion on host specialization should be taken with caution. Furthermore, for the tribe/subfamily level of analysis (only for taxa with ≥ 10 host records), we additionally tested if the indices and measures of host taxonomic and ecological use were inter-correlated. The tests were performed in PAST 3.04.

## Ethics statement

The research is the result of analyses on a dataset compiled with information almost exclusively retrieved from the literature. The single original information comes from a field work in the Maremma Regional Park (Alberese, Grosseto, Italy), which provided the necessary permits to perform the field observations of insects. These observations obey the current Italian law.

## Results

### Brief overview of mutillid hosts

**Fig 3:**
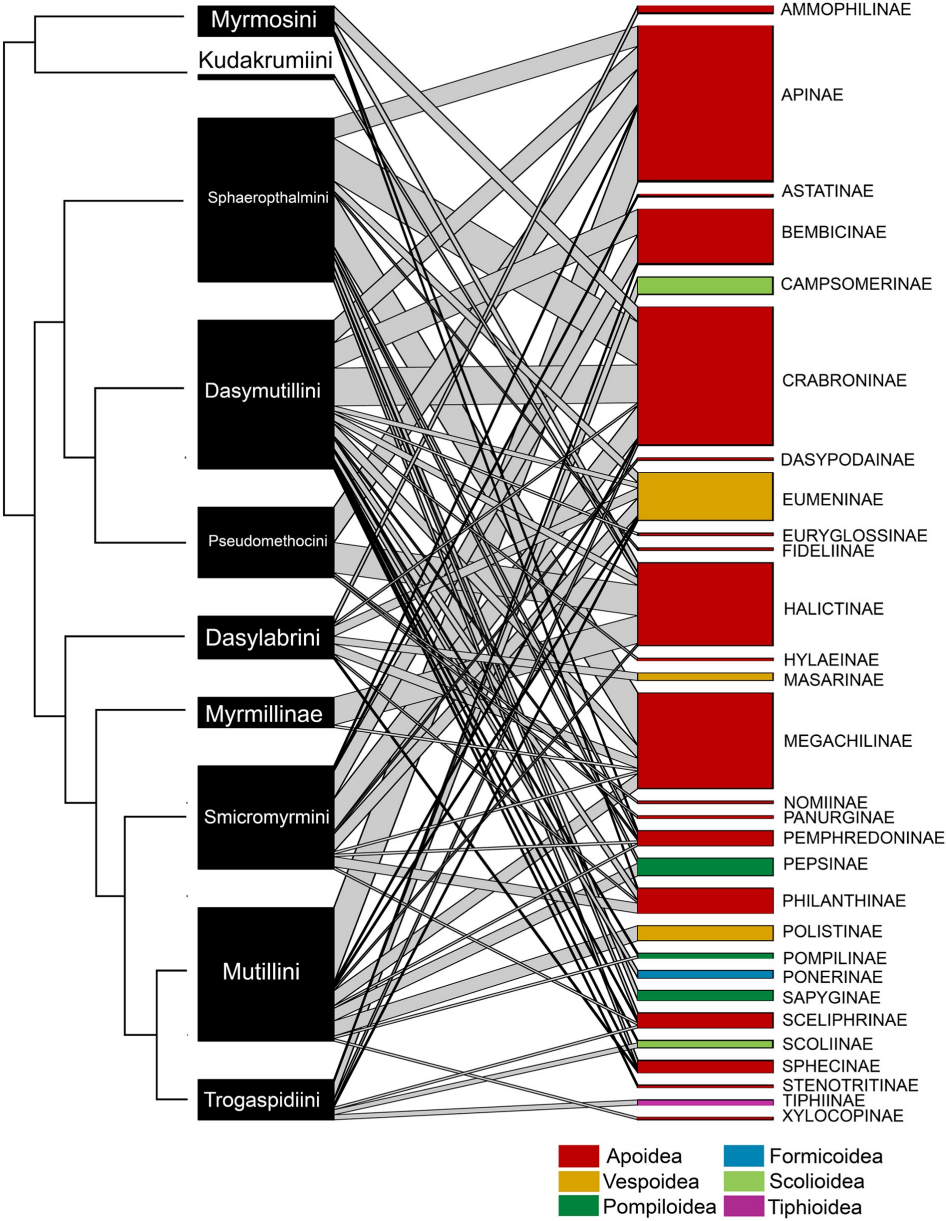
Bipartite graph of the quantitative mutillid–host network. Mutillid nodes are tribes/subfamilies, host nodes are subfamilies. Dendrogram at the left side shows the relationships among mutillid nodes (topology follows the tree of Fig 2). Different colours in the host nodes indicate the superfamilies to which they belong.

We found confirmed host associations for five subfamilies and nine tribes of Mutillidae (Fig 3, S1 Table), which corresponded, respectively, to 62.5% and 69.2% of the total number of valid subfamilies and tribes [2]. Hosts of the 10 main lineages considered here (the 9 tribes *plus* Myrmillinae, which is not divided into tribes) spanned a wide range of aculeate superfamilies, families, subfamilies and genera (Fig 3, Table 1, S1 Table). Overall, 15 families and 29 subfamilies of Aculeata are listed as hosts, spanning all major superfamilies (6) and including bees, predatory wasps, parasitoid wasps and ants (Fig 3, Table 1, S1 Table). Among hosts, Apoidea (bees and apoid wasps) were clearly predominant (19 subfamilies and 82.3% of host records). The bee subfamilies Apinae, Megachilinae, Halictinae and the apoid wasp subfamily Crabroninae are overall heavily used as hosts by mutillids. Out of a total of 102 cited host genera, only 31 covered ≥ 1% of host records (mainly Apoidea: 24 genera). The bee genera *Lasioglossum* and *Bombus*, and the apoid wasp genera *Pison* and *Trypoxylon*, were overall the most abundantly recorded host genera (> 3% each).

**Table 1:** Overview of the information on the taxonomic spectrum of both mutillids and their hosts reviewed in this study. Confirmed host associations are here resumed as lists of host genera and subfamilies overall recorded for each mutillid genus (number of species in brackets and number of records shown in the rightmost column), tribe and subfamily. For the complete information on all host associations at species-level, together with ecological data for each host record and the associated references, see the (S1 Table).

Mutillid subfamily	Mutillid tribe	Mutillid genus	Host subfamilies	Host genera	*N* records
Dasylabrinae	Dasylabrini	*Dasylabris* (2)	Ammophilinae, Sphecinae, Philanthinae, Crabroninae, Eumeninae	*Ammophila*, *Sphex*, *Philanthus*, *Tachysphex*, *Katamenes*	5
		*Dasylabroides* (1)	Ammophilinae, Masarinae	*Ammophila*, *Ceramius*	3
		*Stenomutilla* (3)	Megachilinae, Masarinae, Eumeninae	*Hoplitis*, *Osmia*, *Masaris*, *Leptochilus*,	9
		*Tricholabiodes* (1)	Masarinae	*Quartinia*	1
Mutillinae	Mutillini	*Ephuta* (6)	Pemphredoninae, Pepsinae, Pompilinae	*Diodontus*, *Aupoplus*, *Dipogon*, *Phanagenia*, *Episyron*	7
		*Macromyrme* (1)	Apinae	*Anthophora*	1
		*Mutilla* (7)	Apinae, Megachilinae, Polistinae, Xylocopinae	*Apis*, *Bombus*, *Ceratina*, *Anthidium*, *Megachile*, *Osmia*, *Polistes*	36
		*Ronisia* (2)	Apinae, Crabroninae, Megachilinae, Polistinae	*Anthophora*, *Larra*, *Megachile*, *Polistes*	6
		*Tropidotilla* (1)	Eumeninae, Polistinae	*Paragymnomerus*, *Polistes*	3
	Smicromyrmini	*Ephucilla* (3)	Eumeninae	*Paraleptomenes*, *Phimenes*	3
		*Nemka* (2)	Bembicinae, Megachilinae, Dasypodainae	*Bembecinus*, *Bembix*, *Gorytes*, *Stizus*, *Dasypoda*, *Megachile*	7
		*Physetopoda* (2)	Bembicinae, Crabroninae	*Bembecinus*, *Krombeinictus*	2
		*Promecilla* (5)	Crabroninae, Sceliphrinae, Eumeninae	*Dasyproctus*, *Pison*, *Delta*, *Paraleptomenes*, *Sceliphron*	7
		*Smicromyrme* (5)	Apinae, Astatinae, Bembicinae, Crabroninae, Pemphredoninae, Philanthinae	*Bombus*, *Astata*, *Bembecinus*, *Bembix*, *Gorytes*, *Hoplisoides*, *Tachysphex*, *Crossocerus*, *Miscophus*, *Nitela*, *Oxybelus*, *Palarus*, *Tracheliodes*, *Diodontus*, *Cerceris*, *Philanthus*	22
	Trogaspidiini	*Krombeinidia* (1)	Sceliphrinae	*Sceliphron*	1
		*Timulla* (4)	Bembicinae, Crabroninae, Eumeninae	*Gorytes*, *Liris*, *Tachysphex*, *Eumenes*	4
		*Trogaspidia* (1)	Campsomerinae, Scoliinae, Tiphiinae	*Campsomeris*, *Liacos*, *Megascolia*, *Tiphia*	11
Myrmillinae		*Bethsmyrmilla* (1)	Halictinae	*Lasioglossum*	1
		*Bischoffitilla* (1)	Halictinae	*Lasioglossum*	1
		*Blakeius* (1)	Halictinae	*Lasioglossum*	2
		*Myrmilla* (3)	Halictinae, Megachilinae	*Lasioglossum*, *Halictus*, *Osmia*	7
		*Sigilla* (1)	Halictinae	*Lasioglossum*	1
Myrmosinae	Kudakrumiini	*Myrmosula* (1)	Halictinae	*Lasioglossum*	2
	Myrmosini	*Myrmosa* (2)	Crabroninae, Halictinae, Pemphredoninae	*Crabro*, *Crossocerus*, *Lindenius*, *Lasioglossum*, *Diodontus*	7
		*Paramyrmosa* (1)	Crabroninae, Halictinae, Philanthinae	*Crabro*, *Lasioglossum*, *Cerceris*	5
Sphaeropthalminae	Dasymutillini	*Aglaotilla* (6)	Crabroninae, Megachilinae, Eumeninae	*Aulacophilinus*, *Pison*, *Paralastor*, *Abispa*, *Megachile*	18
		*Dasymutilla* (14)	Apinae, Bembicinae, Megachilinae, Philanthinae, Pompilinae, Sceliphrinae, Sphecinae, Scoliinae	*Anthophora*, *Bombus*, *Diadasia*, *Melitoma*, *Bembecinus*, *Bembix*, *Microbembix*, *Stictia*, *Dianthidium*, *Cerceris*, *Philanthus*, *Anoplius*, *Chalybion*, *Scolia*, *Sphex*	25
		*Ephutomorpha* (6)	Euryglossinae, Halictinae, Hylaeinae, Stenotritinae	*Xanthesma*, *Lasioglossum*, *Amphylaeus*, *Stenotritus*	6
		*Lomachaeta* (2)	Crabroninae, Pemphredoninae	*Solierella*, *Diodontus*	3
		*Ponerotilla* (3)	Ponerinae	*Brachyponera*	3
		*Reedomutilla* (1)	Apinae	*Melissoptila*	1
		*Traumatomutilla* (2)	Apinae, Bembicinae	*Diadasina*, *Monoeca*, *Bicyrtes*	3
	Pseudomethocini	*Anomophotopsis* (1)	Halictinae	*Paroxystoglossa*	1
		*Calomutilla* (1)	Halictinae	*Pseudaugochlora*	1
		*Euspinolia* (2)	Apinae	*Anthophora*, *Melitoma*	2
		*Hoplomutilla* (5)	Apinae	*Centris*, *Epicharis*, *Eulaema*, *Eufrisea*	5
		*Horcomutilla* (1)	Apinae	*Exomalopsis*	1
		*Lophomutilla* (1)	Halictinae	*Lasioglossum*	1
		*Lophostigma* (1)	Halictinae	*Megalopta*	2
		*Lynchiatilla* (1)	Halictinae	*Paroxystoglossa*	1
		*Pappognatha* (5)	Apinae	*Euglossa*	5
		*Pseudomethoca* (7)	Apinae, Halictinae, Nomiinae, Panurginae	*Exomalopsis*, *Lasioglossum*, *Augochloropsis*, *Nomia*, *Perdita*	10
	Sphaeropthalmini	*Cystomutilla* (1)	Crabroninae, Pemphredoninae	*Ectemnius*, *Pemphredon*	2
		*Dilophotopsis* (1)	Crabroninae	*Tachysphex*	1
		*Morsyma* (1)	Pemphredoninae	*Diodontus*	1
		*Odontophotopsis* (1)	Crabroninae	*Oxybelus*	1
		*Sphaeropthalma* (9)	Apinae, Crabroninae, Eumeninae, Fideliinae, Megachilinae, Pepsinae, Sapyginae, Sceliphrinae, Sphecinae	*Notanthidium*, *Ancistrocerus*, *Anthidium*, *Anthophora*, *Ashmeadiella*, *Atoposmia*, *Auplopus*, *Diadasia*, *Dianthidium*, *Hoplitis*, *Isodontia*, *Leptochilus*, *Megachile*, *Melissodes*, *Microdynerus*, *Neofidelia*, *Osmia*, *Pisonopsis*, *Sapyga*, *Sceliphron*, *Symmorphus*, *Tachysphex*, *Trypoxylon*	53
		*Tallium* (1)	Apinae	*Centris*	1
		*Xystromutilla* (2)	Crabroninae, Sceliphrinae	*Trypoxylon*, *Podium*	4

### Host use at mutillid tribe/subfamily level

#### Host taxonomic diversity

Tribes/subfamilies of Mutillidae seemed to vary in their host use. Among the most basal subfamily (Myrmosinae), Kudrakumini were recorded attacking bees in the subfamily Halictinae (Table 1), but there are only two records available. Myrmosini, the other tribe in this basal subfamily, attack a variety of bees and wasps in the Apoidea (4 subfamilies) (Fig 3, Table 1, S1 Table). Among Sphaeropthalminae, Sphaeropthalmini attack a total of 10 host subfamilies spanning bees and wasps from Apoidea, Vespoidea and Pompiloidea (mainly bees in the family Megachilidae and apoid wasps in the family Crabroninae). Dasymutillini parasitize hosts from 15 subfamilies (mainly apoid wasps in Crabroninae) spanning 5 out of the 6 superfamilies recorded overall as hosts for mutillids, and they are also notable to use (rarely) ants as hosts. Pseudomethocini slightly differed having a narrower host range including bees from 4 subfamilies, particularly Apinae and Halictinae (Fig 3, Table 1, S1 Table). Dasylabrini, the only tribe in the Dasylabrinae, attack bees and wasps from 7 subfamilies in 2 superfamilies (Apoidea and Vespoidea). Myrmillinae are associated with bees of the subfamilies Halictinae (most cases) and Megachilinae (Fig 3, Table 1, S1 Table). Among Mutillinae, Smicromyrmini parasitize hosts from 10 subfamilies of bees, particularly, wasps in the Apoidea and Vespoidea. Mutillini attack hosts from 9 subfamilies, being bees in the Apinae predominant. Trogaspidiini depart from the other tribes in the Mutillinae by attacking, among other taxa, wasps from the otherwise rarely recorded parasitoid superfamilies Scolioidea and Tiphioidea (Fig 3, Table 1, S1 Table).

**Fig 4:**
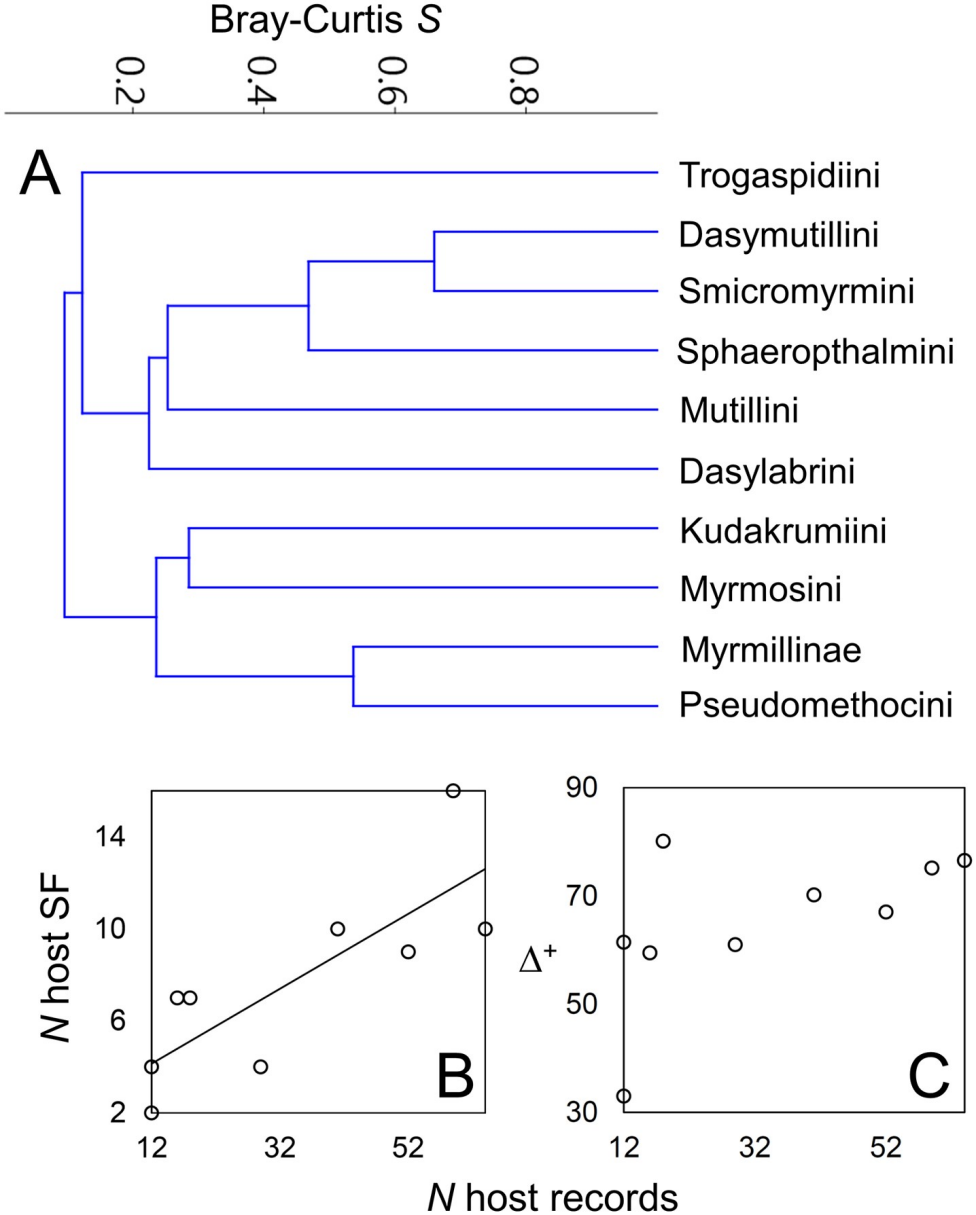
**A**, Dendrogram resulting from the cluster analysis of mutillid tribes/subfamilies, based on Bray-Curtis similarity (*S*) of host use (i.e. host subfamilies’ abundances). **B**, Relationship between the number (*N*) of host records and the number of recorded host subfamilies (SF) across the mutillid tribes/subfamilies with ≥ 10 host records. **C**, Relationship between the number (*N*) of host records and the average taxonomic diversity (Δ^+^) across the mutillid tribes/subfamilies with ≥ 10 host records. Trend line is shown only for the significant linear correlation in **B**.

A cluster analysis depicted three main groups (Fig 4A). One group includes Dasymutillini, Smicromyrmini, Sphaeropthalmini, Mutillini and Dasylabrini, which are the lineages showing a wider host range from several to many subfamilies. Another group includes Myrmosini, Kudakrumiini, Pseudomethocini and Myrmillinae, which have a narrower host range and shared the abundant use of Halictinae. The last group, distant from the other two, includes only Trogaspidiini, which were unique in frequently attacking parasitoid wasps from lineages not used by other tribes/subfamilies. Thus, no apparent accordance between host use diversity and phylogeny is visible at this level of analysis (Figs 2 and 4A). Such variable host taxonomic use leads to an overall moderate value of complementarity (i.e. specialization) (*H*_*2*_*’* = 0.40), low mean number of shared hosts (2.26) and low host niche overlap (Horn’s index = 0.27) of the whole network, suggesting a certain degree of specialization for Mutillidae as a whole.

Accordingly, the observed variability in host use among tribes/subfamilies rewarded variable values of the network indices of each lineage. Complementarity (*H*_*2*_*’*) was higher in Dasylabrini, Dasymutillini, Mutillini, Pseudomethocini, Smicromyrmini and Trogaspidiini (≥ 0.5) than in Myrmillinae, Myrmosini and Sphaeropthalmini (≤ 0.4) (Table 2). The mean number of shared hosts was higher in these last three tribes/subfamilies, as well as in Dasylabrini and in Mutillini (> 0.6) than in the other tribes (< 0.6) (Table 2). Niche overlap (Horn’s index) was highest in Myrmillinae (0.99) and null to low in Trogaspidiini, Dasylabrini, Dasymutillini and Mutillini (< 0.25) (Table 2). Average taxonomic distinctness (Δ^+^) was lowest in Myrmillinae (33.0) and highest in Dasylabrini (80.1) (Table 2). Variation in taxonomic distinctness (Λ^+^) was clearly higher in Mutillini (1176.0) than in the other tribes/subfamilies, which had more similar values (440.1–603.9); this great uneven distribution in Mutillini is related with the fact that *Bombus* (Apidae: Apinae) accounts for most of records, followed by *Polistes* (Vespidae: Polistinae), a distant lineage to *Bombus* (Table 2). While the number of host records *per* tribe/subfamily was positively correlated with the number of recorded host subfamilies (Spearman test, ρ = 0.86, P = 0.004), it was not correlated with Δ^+^, Λ^+^ or any of the network indices (*H*_*2*_*’*, mean number of shared hosts, Horn’s index) (ρ ≤ 0.7, P > 0.06). *H*_*2*_*’* was negatively correlated both with the mean number of shared hosts (ρ = -0.82, P = 0.01) and the Horn’s index (ρ = -0.76, P = 0.02). The other indices or measures of host taxonomic diversity/specialization were not inter-correlated (-0.41 ≤ ρ ≤ 0.56, P > 0.11).

**Table 2:** Number of records (*N* records) and host (subfamily-level) taxonomic diversity parameters of the 9 reviewed mutillid tribes/subfamilies with ≥ 10 host records.

Mutillid tribe	*N* records	*N* host SF	Δ^+^	Λ^+^	*H*_*2*_*’*	Mean *N* shared	Horn’s index
Dasylabrini	18	7	80.1	603.9	0.55	0.83	0.18
Dasymutillini	59	16	75.2	440.1	0.72	0.29	0.14
Mutillini	52	9	67.1	1176.0	0.60	0.70	0.22
Myrmillinae	12	2	33.0	490.8	0.00	1.00	0.99
Myrmosini	12	4	61.5	492.4	0.29	2.00	0.46
Pseudomethocini	29	4	61.0	489.7	0.56	0.56	0.47
Smicromyrmini	41	10	70.2	535.1	0.55	0.56	0.45
Sphaeropthalmini	64	10	76.6	517.8	0.40	0.62	0.30
Trogaspidiini	16	7	59.5	509.8	1.00	0.00	0.00

*N* host SF = number of host subfamilies, Δ^+^ = average taxonomic distinctness, Λ^+^ = variation in taxonomic distinctness, *H*_*2*_*’* = network specialization. Mean *N* shared = mean number of host subfamilies shared by any two mutillid tribes/subfamilies, Horn’s index = mean similarity in interaction pattern between mutillid tribes/subfamilies (i.e. degree of niche overlap).

#### Host ecological profile

**Fig 5:**
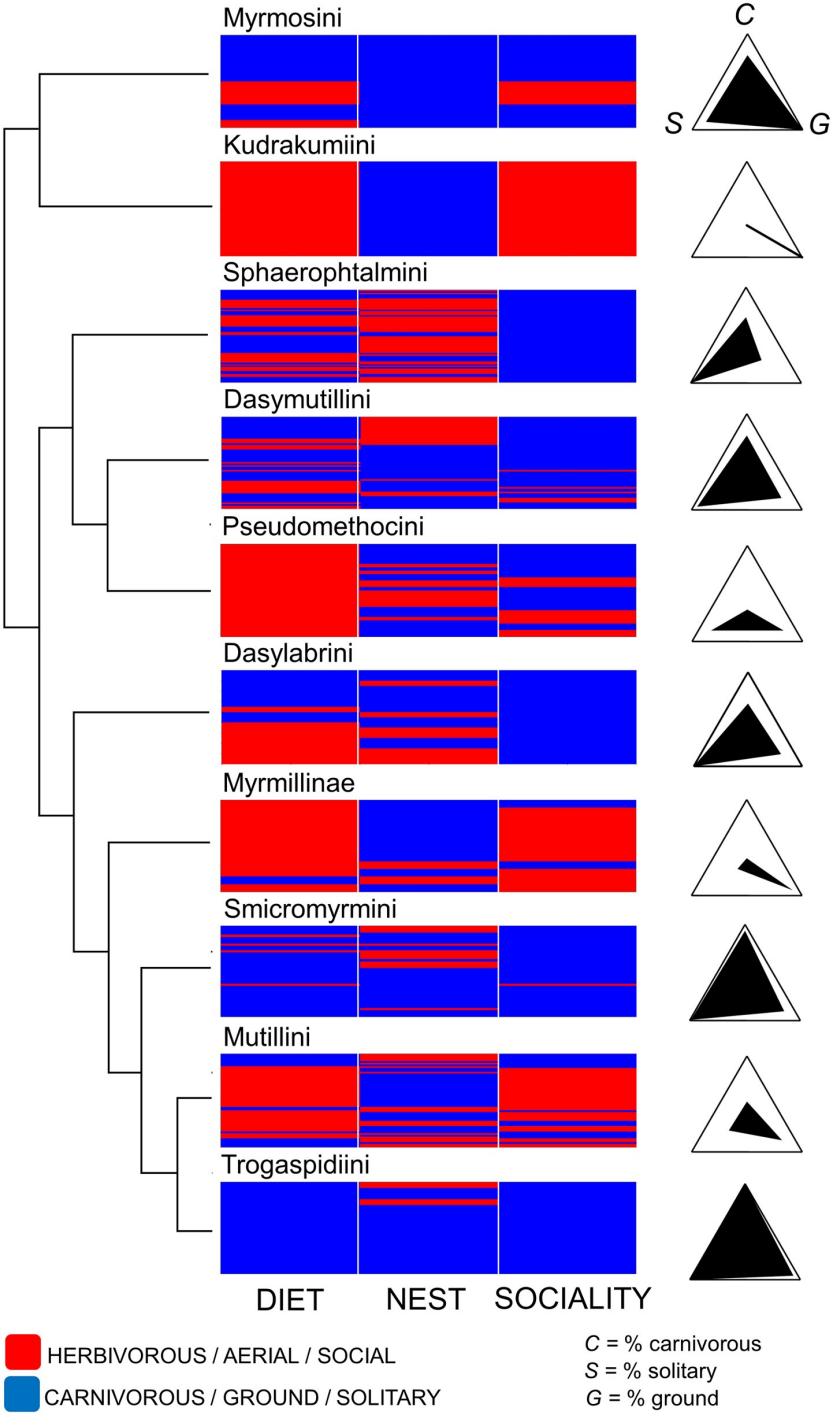
Graphical representation of host use in mutillid tribes/subfamilies according to host ecological traits (larval diet (DIET), nest type (NEST) and SOCIALITY). At the leftmost side there is the dendrogram showing the relationships among mutillid tribes/subfamilies (topology follows the tree of Fig 2). The matrix plots, right close to the dendrogram, show all the individual host records for each mutillid tribe/subfamily; each line is a host record and it is represented by the combination of blue (carnivorous, ground, solitary) and/or red (herbivorous larvae, aerial, social) host states. On the rightmost side, there are triangular plots showing the % frequency of the host ancestral states over the total number of records; in each triangular plot, the upper apex identifies the % of carnivorous (*C*), the left apex identifies the % of solitary (*S*) and the right apex identifies the % of ground (*G*). Thus, a completely black triangle means that all records refer to carnivorous, ground-nesting and solitary hosts, while the opposite situation will result in a completely white triangle.

Different tribes/subfamilies showed an important variability in host ecological traits. Smicromyrmini and Trogaspidiini have clearly similar host ecological profiles, attacking mostly hosts with all the three ancestral states, i.e. ground-nesting solitary wasps (Fig 5). On the other hand, Kudrakumiini, Myrmillinae and Mutillini have a high proportion of hosts which are herbivorous at the larval stage (i.e. bees), nest in the ground and that are social (Fig 5). The remaining 5 tribes attack exclusively or mostly solitary hosts, but a higher variability was found concerning larval diet (except for Pseudomethocini, which only attack bees) and nest type of the hosts (Fig 5). However, while Sphaeropthalmini were peculiar in using mainly aerial-nesting hosts, the other tribes/subfamilies showed a general preference for ground-nesting wasps (Fig 5). The variation in host ecological profile weakly associated with phylogenetic relationships among tribes/subfamilies. While tribes in Sphaeropthalminae seem reasonably similar, within the other subfamilies the tribes showed striking differences (Fig 5). Myrmillinae, which falls in a large clade including Dasylabrinae and Mutillinae, had a host ecological profile similar to Mutillini (Fig 5). The % of solitary hosts, the % of ground-nesting hosts and the % of carnivorous hosts were not inter-correlated and were not correlated with the indices or measures of host taxonomic diversity/specialization (-0.64 ≤ ρ ≤ 0.61, P > 0.09). However, one notes that higher values of Δ^+^ and lower values of Horn’s index were more often recorded for tribes/subfamilies with higher % of solitary hosts (Table 2, Fig 5).

### Host use at mutillid genus level

#### Host taxonomic diversity

**Fig 6:**
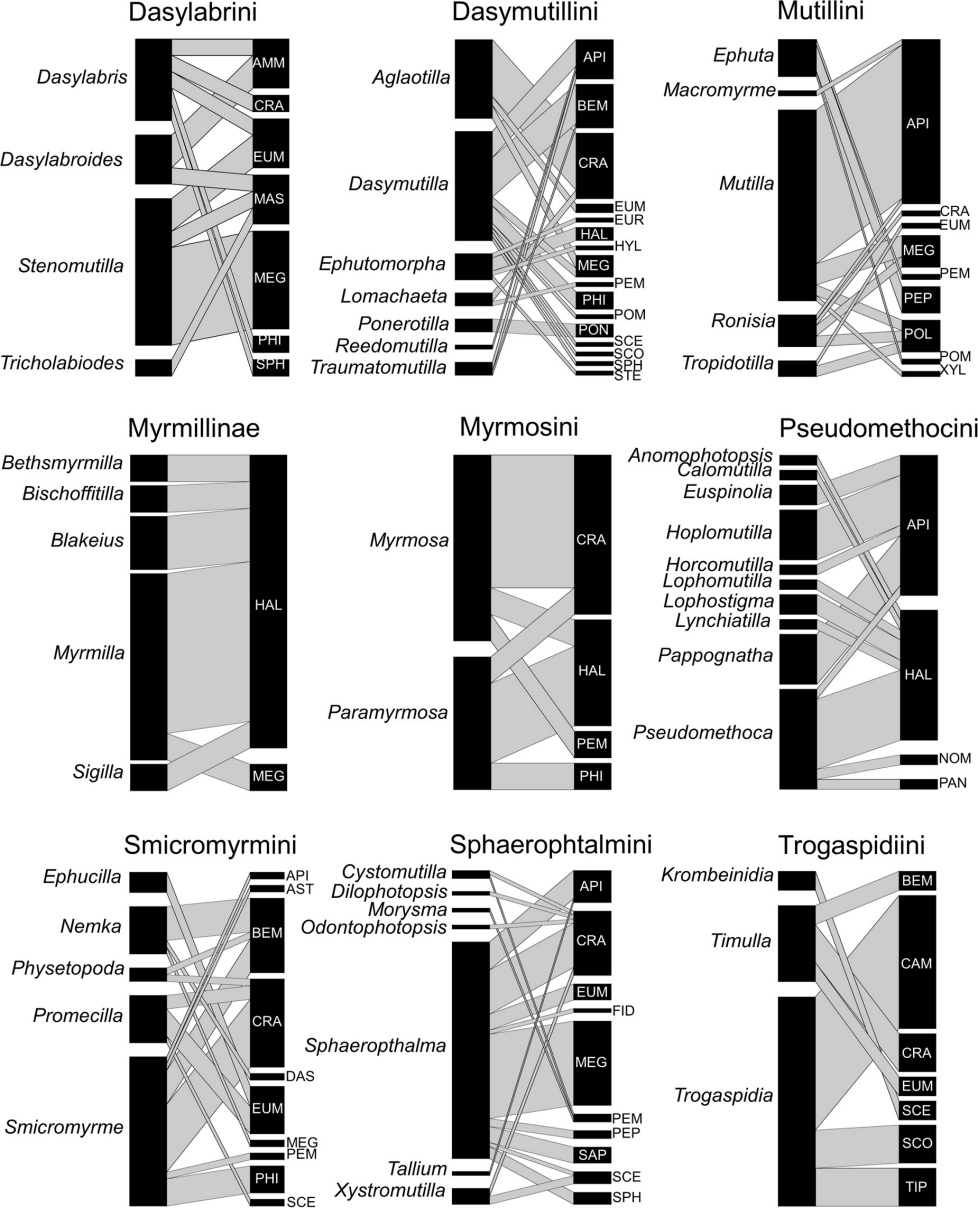
Bipartite graphs of the quantitative mutillid–host networks, one per each of the 9 tribe/subfamily with ≥ 10 host records. Mutillid nodes are genera, host nodes are subfamilies. Codes for host subfamilies: AMM = Ammophilinae, API = Apinae, AST = Astatinae, BEM = Bembicinae, CAM = Campsomerinae, CRA = Crabroninae, DAS = Dasypodainae, EUM = Eumeninae, EUR = Euryglossinae, FID = Fideliinae, HAL = Halictinae, HYL = Hylaeinae, MAS = Masarinae, MEG = Megachilinae, NOM = Nomiinae, PAN = Panurginae, PEM = Pemphredoninae, PEP = Pepsinae, PHI = Philanthinae, POL = Polistinae, POM = Pompilinae, PON = Ponerinae, SAP = Sapyginae, SCE = Sceliphrinae, SCO = Scoliinae, SPH = Sphecinae, STE = Stenotritinae, TIP = Tiphiinae, XYL = Xylocopinae.

Within the tribes/subfamilies with ≥ 10 host records, different genera also showed some variation in host use, though inter-generic differences seemed very variable (Fig 6, Table 1, S1 Table). Some tribes seem to present visible variation among genera (Fig 6). In Dasylabrini, *Dasylabris* and *Stenomutilla* attack 5 and 3 host subfamilies respectively, but they only share the vespid wasp subfamily Eumeninae as host. In Dasymutillini, *Dasymutilla* is mainly confined to hosts in the apoid wasp subfamilies Bembicinae and Philanthinae and in the bee subfamily Apinae, while *Aglaotilla* essentially attacks apoid wasps in the subfamily Crabroninae. Within Mutillini, *Mutilla* is clearly associated with bees in the Apinae and secondarily with Megachilinae, while *Ephuta* is typically associated with pompiloid wasps (Pompilinae and Pepsinae). In Pseudomethocini, *Haplomutilla* and *Pappognatha* are exclusively associated with Apinae bees, while *Pseudomethoca* is almos exclusively associated with Halictidae (Halictinae and Nomiinae). In Smicromyrmini, besides the widely generalist *Smicromyrme*, which attacks 6 out of the 10 host subfamilies recorded for the tribe, differences can be found between *Nemka* (more associated with apoid wasps in Bembicinae) and *Promecilla* (more associated with Crabroninae (Apoidea) and Eumeninae (Vespoidea)). In Trogaspidiini, *Timulla* and *Trogaspidia* clearly differed in that the latter is uniquely associated with scolioid and tiphioid parasitoid wasps (Fig 6, Table 1, S1 Table). In other cases, differences among genera seem to be very weak. In Myrmillinae, all genera exclusively or mostly attack Halictinae bees. In Myrmosini, while *Myrmosa* is mainly associated with apoid wasps and *Paramyrmosa* mainly with bees, the two genera shared 2 out of the 4 host subfamilies recorded for the tribe. In Sphaeropthalmini, *Sphaeropthalma* is fairly generalist, attacking 9 out of the 10 host subfamilies recorded for the tribe, while the other genera have too few host records (1–4) to suggest any degree of specialization.

The cluster analyses produced dendrograms in which these differences among genera in each tribe/subfamily can be explored by looking at Bray-Curtis similarities. The phylogenetic relationships among genera are not well resolved for many tribes/subfamilies, so it is difficult at the moment to suggest any effect of common ancestry on the host at this level of analysis. In general, the cluster analyses do not seem to point towards an effect of phylogenetic relationships on host use within most tribes/subfamilies (Figs 2 and 6). However, an effect may be possible in Simcromyrmini, where closely related pairs of genera (*Smicrmomyrme* and *Nemka* vs. *Promecilla* and *Ephucilla*) fall in different clusters based on their host use (Figs 2 and 6), and in Mutillini, where *Ephuta*, which differed from the other genera in host use (Fig 6) is also distant from all the other genera in the phylogeny (Fig 2).

Variation in host use among genera was also visible by inspecting the network and diversity indices for the genera with ≥ 10 host records. Complementarity (*H*_*2*_*’*) was higher in *Aglaotilla*, *Dasymutilla*, *Pseudomethoca* and *Mutilla* (> 0.65), while it was lower in *Smicromyrme* and *Sphaeropthalma* (< 0.45) (Table 3). Conversely, the mean number of shared hosts was higher in these two latter genera (> 0.8) than in the other tribes (< 0.5) (Table 2). All genera have low to moderate (0.17–0.41) niche overlap (Horn’s index) (Table 3). Average taxonomic distinctness (Δ^+^) was lowest in *Mutilla* (43.0) and highest in *Sphaeropthalma* (76.5) (Table 3). Variation in taxonomic distinctness (Λ^+^) was very high in *Aglaotilla* and *Trogaspidia* (> 1150) and lowest in *Smicromyrme* (166); in the latter, distribution of taxonomic distinctness was more even since essentially all hosts come from several Crabronidae subfamilies (Table 3).

**Table 3:** Number of records (*N* records) and host (subfamily-level) taxonomic diversity parameters of the 7 reviewed mutillid genera with ≥ 10 host records.

Mutillid genus	*N* records	*N* host SF	Δ^+^	Λ^+^	*H*_*2*_*’*	Mean *N* shared	Horn’s index
*Aglaotilla*	18	3	53.7	1153.0	0.76	0.47	0.41
*Dasymutilla*	25	8	71.7	473.0	0.68	0.21	0.17
*Mutilla*	36	4	43.0	944.3	0.68	0.33	0.33
*Pseudomethoca*	10	4	51.6	666.5	1.00	0.29	0.29
*Smicromyrme*	22	6	55.8	166.3	0.14	1.20	0.38
*Sphaeropthalma*	53	9	76.5	589.1	0.45	0.83	0.25
*Trogaspidia*	11	3	56.7	1160.0	-	-	-

*N* host SF = number of host subfamilies, Δ^+^ = average taxonomic distinctness, Λ^+^ = variation in taxonomic distinctness, *H*_*2*_*’* = network specialization. Mean *N* shared = mean number of host subfamilies shared by any two mutillid genera, Horn’s index = mean similarity in interaction pattern between mutillid genera (i.e. degree of niche overlap). “-” indicates that calculations were not performed because only one species in the genus had data (i.e. no network possible).

The number of host records *per* genus was neither correlated with the number of recorded host subfamilies (Spearman test, ρ = 0.66, P = 0.15), or with any of the diversity or network indices (ρ ≤ 0.20, P ≥ 0.20), suggesting that variability within genera is not affected by sample size.

#### Host ecological profile

**Fig 7:**
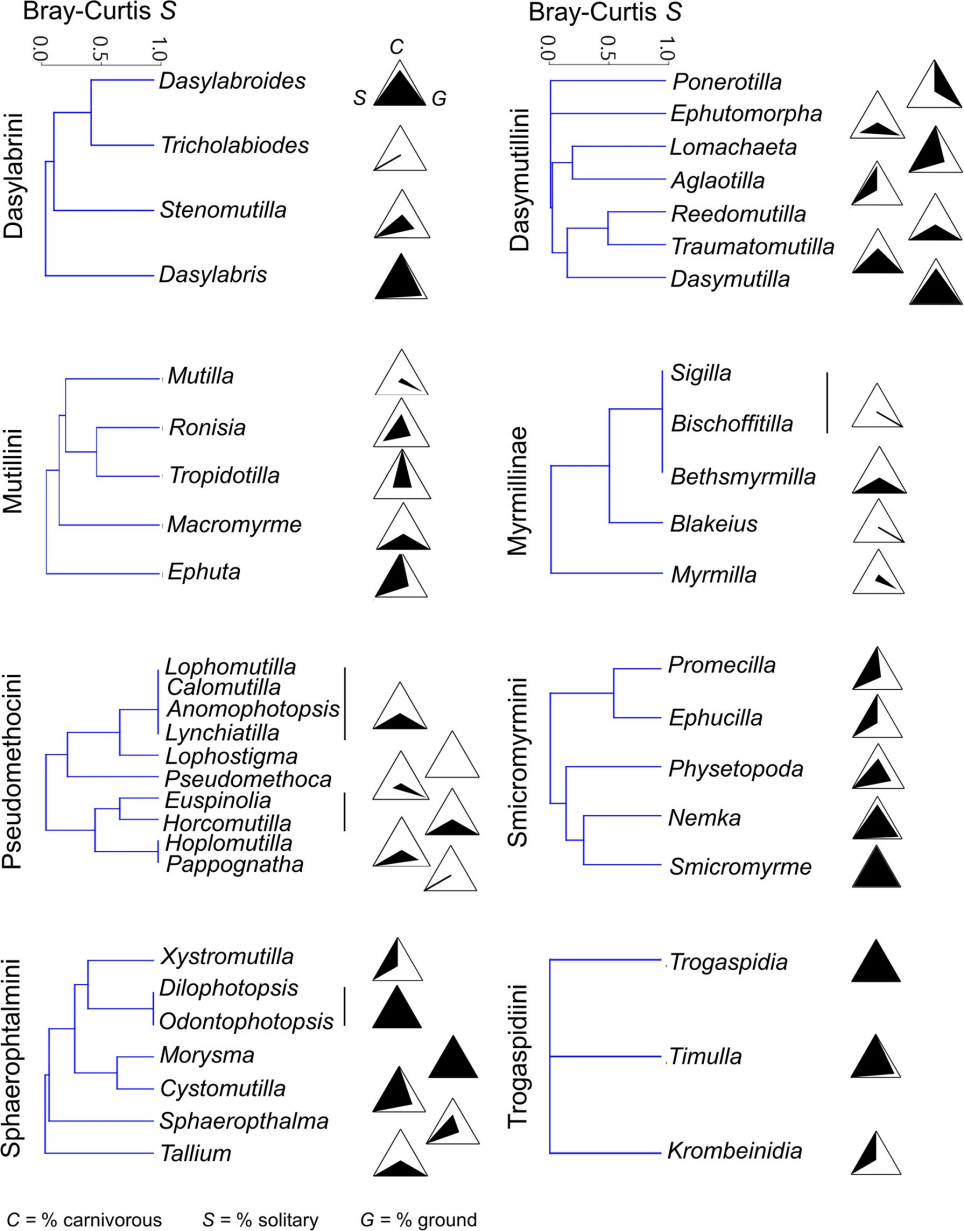
Dendrograms resulting from the cluster analyses of mutillid genera in each of the 8 tribes/subfamilies with at least two genera, based on Bray-Curtis similarity (*S*) of host use (i.e. host subfamilies’ abundances). On the right side of the dendrograms, there are triangular plots showing the % frequency of the host ancestral states over the total number of records, *per* each mutillid genus; in each triangular plot, the upper apex identifies the % of carnivorous (*C*), the left apex identifies the % of solitary (*S*) and the right apex identifies the % of ground (*G*). Thus, a completely black triangle means that all records refer to carnivorous, ground-nesting and solitary hosts, while the opposite situation will result in a completely white triangle.

Different genera within tribes/subfamilies show a variable host ecological profile, though this variability is better visible in only some of them. While we have produced plots for all genera (Fig 7), we here describe only those with at least two host records. Variability in host ecological profile was apparently very low in Myrmillinae, in which all genera attack ground-nesting social bees with only one case of ground-nesting solitary bees (Fig 7). In Trogaspidiini variability was also low, with almost all cases concerning ground-nesting solitary wasps (Fig 7). In Sphaeropthalmini, *Sphaeropthalma* seems quite generalist in host ecological profile but is mainly associated with solitary aerial-nesting wasps, as also *Xystromutilla* and *Cystomutilla* (Fig 7). A higher inter-generic variability can be found in other lineages. Dasylabrini have genera mostly associated with soilitary ground-nesting wasps (*Dasylabris*, *Dasylabroides*) and other most associated with solitary bees (with variable nest type) (*Stenomutilla*). In Smicromyrmini, *Smicrmomyrme* and *Nemka* attack mostly solitary ground-nesting wasps, while *Promecilla* and *Ephucilla* mostly parasitize solitary aerial-nesting wasps (Fig 7). In Pseudomethocini, *Paseudomethoca* attacks a mix of solitary and social (mostly) ground-nesting bees, while *Pappognatha* attacks almost exclusively solitary aerial-nesting bees (Fig 7). Within Mutillini, *Mutilla* strongly differed from other genera in attacking almost exclusively ground-nesting social bees, with wasps more common as hosts in other genera (Fig 7). In Dasymutillini, the main differences appeared among *Ponerotilla*, attacking ants (i.e. carnivorous, ground nesting and social), *Dasymutilla*, almost exclusively attacking solitary ground-nesting wasps, *Ephutomorpha*, most likely associated with ground-nesting social bees, and *Aglaotilla*, mostly associated with solitary aerial-nesting wasps (Fig 7).

An effect of common ancestry on the variability of host ecological profiles within tribes/subfamilies was unclear. From one side, in Dasylabrini it is possible to see how closely related genera (*Dasylabris*, *Stenomutilla*) have quite different host ecological profiles, and that the more distant *Dasylabroides* shows similarity with *Dasylabris* (Figs 2 and 7). On the opposite side, in Simcromyrmini, more closely related pairs of genera (*Smicrmomyrme* and *Nemka* vs. *Promecilla* and *Ephucilla*) effectively have more similar host ecological profiles (Figs 2 and 7). Similarly, in Mutillini, *Ephuta*, which is unique in the tribe by attacking mainly aerial-nesting solitary wasps, is indeed phylogenetically distant from the other genera (*Mutilla*, *Tropidotilla*).

### Host use at mutillid species level

#### Host taxonomic diversity

**Fig 8:**
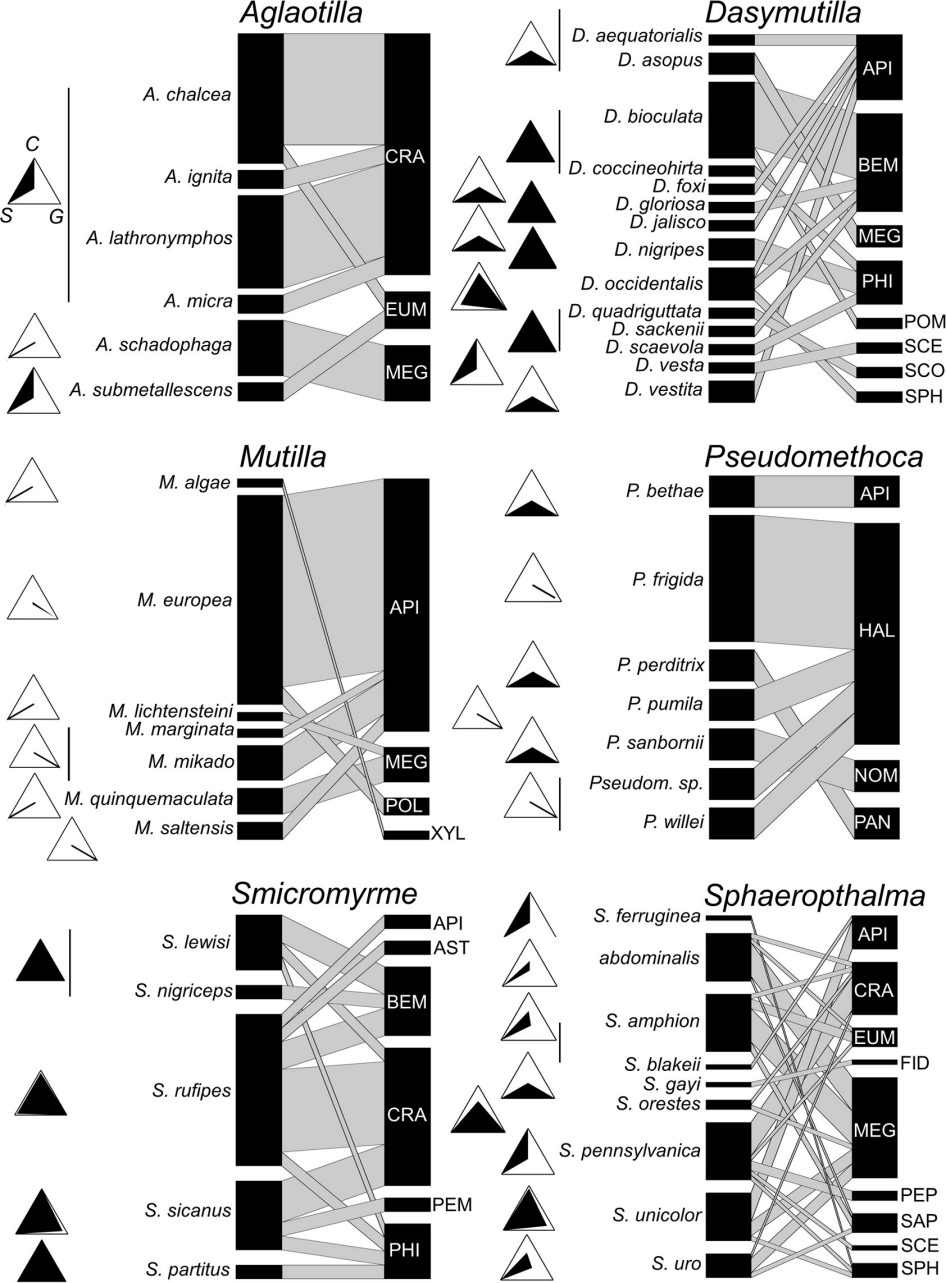
Bipartite graphs of the quantitative mutillid–host networks, one per each of the 6 genera with ≥ 10 host records. Mutillid nodes are species, host nodes are subfamilies. Codes for host subfamilies: API = Apinae, AST = Astatinae, BEM = Bembicinae, CRA = Crabroninae, EUM = Eumeninae, FID = Fideliinae, HAL = Halictinae, MEG = Megachilinae, NOM = Nomiinae, PAN = Panurginae, PEM = Pemphredoninae, PEP = Pepsinae, PHI = Philanthinae, POL = Polistinae, POM = Pompilinae, SAP = Sapyginae, SCE = Sceliphrinae, SPH = Sphecinae, XYL = Xylocopinae. On the left side of the bipartite graphs, there are triangular plots showing the % frequency of the host ancestral states over the total number of records, *per* each mutillid species; in each triangular plot, the upper apex identifies the % of carnivorous (*C*), the left apex identifies the % of solitary (*S*) and the right apex identifies the % of ground (*G*). Thus, a completely black triangle means that all records refer to carnivorous, ground-nesting and solitary hosts, while the opposite situation will result in a completely white triangle.

The reduced sample of host records for most species limited the detection of variability in host use among species within genera. Apparently, some differences occur within certain genera (S1 Table, Fig 8). In *Mutilla*, *M*. *europea* and *M*. *mikado* seemed largely specialized on *Bombus* (Apinae), while *M*. *quinquemaculata* seems specialized in attacking Megachilinae. Within *Aglaotilla*, *A*. *chalcea* and *A*. *lathronymphos* attack almost exclusively Crabroninae, while *A*. *schadophaga* attacks Megachilinae. Within *Sphaeropthalma*, *S*. *abdominalis*, *S*. *amphion* and *S*. *uro* seemed more specialized in attacking Megachilinae, while *S*. *pennsylvanica* is more associated with Crabroninae and *S*. *unicolor* is more associated with Apinae. Other genera (e.g. *Smicromyrme*, *Myrmilla*) seem to include species that largely overlap their host taxonomic spectrum (S1 Table). Considering only those species with ≥ 10 host records, average taxonomic distinctness (Δ^+^) was lowest in *M*. *europaea* (34.3) and highest in *S*. *amphion* (75.8) (Table 4). Variation in taxonomic distinctness (Λ^+^) reached high values (> 800) in five out of the seven analysed species, suggesting that an uneven distribution of host use across the taxonomic tree is common at the species level. However, *Smicromyrme rufipes* had low Λ^+^ (184.3), according with the use of host species essentially from few subfamilies of Crabronidae (Table 4). Also across species, the number of host records was not correlated with the number of recorded host subfamilies, Δ^+^ or Λ^+^ (Spearman test, ρ ≤ -0.21, P ≥ 0.60).

**Table 4:** Number of records (*N* records) and host (subfamily-level) taxonomic diversity parameters of the 7 reviewed mutillid species with ≥ 10 host records.

Mutillid species	*N* records	*N* host SF	Δ^+^	Λ^+^
*Mutilla europaea*	24	2	34.3	846.9
*Smicromyrme rufipes*	11	6	57.8	184.3
*Sphaeropthalma abdominalis*	10	4	65.8	1007.0
*Sphaeropthalma amphion*	12	4	75.8	879.0
*Sphaeropthalma pennsylvanica*	11	5	71.6	1174.0
*Sphaeropthalma unicolor*	10	3	61.3	558.2
*Trogaspidia castellana*	11	3	56.7	1160.0

*N* host SF = number of host subfamilies, Δ^+^ = average taxonomic distinctness, Λ^+^ = variation in taxonomic distinctness.

#### Host ecological profile

Host ecological profiles seemed quiet conserved within genera. In *Aglaotilla*, all species attack solitary aerial-nesters, most often wasps. In *Mutilla*, the commonest hosts are ground-nesting social bees. All *Dasymutilla* species are associated with solitary ground nesting wasps and bees. All *Pseudomethoca* species attack ground nesting, most often social, bees. *Smicromyrme* species almost invariably were found in associations with ground nesting, solitary wasps. In *Sphaeropthalma*, the genus with the highest number of species (4) with abundant host records (≥ 10), includes species attacking almost exclusively aerial-nesting, always solitary hosts, suggesting that, at least within some genera, the few differences in host ecology among species may be limited to larval diet of the host rather than involving adult nesting behaviour (nest type and sociality).

## Discussion

While some groups of aculeate hymenopterans largely include species that parasitize a single or a few host species [49], generally hymenopteran parasitoids are found in nature to attack several to many different hosts [47]. Also velvet ants have, overall, a wide range of hosts. The use of a wide host taxonomic range may be favoured by the peculiar morphological adaptations in mutillids, such as a robust cuticle and a powerful sting, which may allow them to exploit many host species with different defense strategies. Furthermore, host preference is not genetically based in Mutillidae, since larvae successfully develop on non-hosts in the laboratory [76]. However, the degree of host taxonomic specialization oscillates from fairly specialized associations to more generalist ones, with such variation visible at least at the levels of tribe/subfamily and genus, and to a lesser extent at the level of species. Such important variation in host taxonomic use was apparent from all diversity and network measures/indices. Our explorative analysis suggests that this wide variation in specialization does not depend on phylogenetic relationships, but likely depends on ecological traits of hosts. Indeed, tribes/subfamilies of mutillids with wide taxonomic host range but restricted host ecology were common. On the other hand, mutillid lineages that appear taxonomically specialized often also attack hosts with very similar ecology (e.g. Myrmillinae on Halictinae). Interestingly, we found a wide range of variation in host taxonomic distinctness (Λ^+^) among mutillid lineages (with a greater occurrence of high values of Λ^+^ apparently at species-level), which may suggest an evolutionary tendency to specialize on few taxonomically unrelated clusters of closely related species. Again, these unrelated clusters of host species share important ecological traits (e.g. social behavior for *Bombus* and *Polistes*, largely exploited by Mutillini; aerial nesting for *Pison* and *Megachile*, preferred hosts of *Aglaotilla*), supporting our hypothesis of a greater role of host ecology on host use.

The influence of the host habitat is an important factor in host choice by parasitoids [48]. Mutillid females are apterous and our analysis shows a significant preference for hosts nesting in the ground. However, interestingly, some genera seem specialized in attacking aerial-nesting hosts. These aerial-nesting hosts are almost exclusively solitary, suggesting that mutillids may have a hard job to sneak into social hosts’ aerial nests, likely because these nests typically have many workers guarding on the nest surface. For instance, social stingless bees (Meliponinae), which are common in the tropics, where mutillid diversity is high [77], were never reported as hosts. Exceptional, rare cases however indicate that some mutillids may have evolved specific adaptations (e.g. chemical insignificance, i.e. a cuticular hydrocarbon profile harboring lower concentrations of recognition cues [78]) that allow them specializing in attacking aerial-nesting social wasps (*Polistes*) and perhaps bees (*Apis*). On the other hand, host sociality does not seem to be a strong limitation for ground-nesting hosts, likely because these nests have typically a single guard on a small entrance [58, 61]. Again, rare exceptions exist and can be due to particular coevolution patterns with certain hosts, notably the case of *Mutilla* and its preferred host *Bombus*, a social, ground-nesting bee genus whose colonies are defended by many workers at a large nest entrance [58].

The greater role of host ecology over host taxonomic group in mutillid specialization is also suggested by the fact that host size not necessarily represents a constraint in host use. Since mutillid females can dig through the host nest entrance, they may invade nests of small and large host species (i.e. nests with both small and large entrance). A single, multivoltine velvet ant species may vary in size across its yearly generations, since host species—and thus food mass—of different size are available at different times of the year [79, 80]. This may increase the taxonomic spectrum of larger mutillid species but not necessarily enlarge the host ecological profile.

One hypothesis that may explain the observed specialization in host ecology by many mutillid taxa would invoke competition as an important evolutionary force for host use [50]. Thus, mutillids may differentiate host ecological profiles to reduce competition. For example, some velvet ant species may have shift from ground-nesting to aerial-nesting hosts, a less commonly represented ecological host profile. This hypothesis is preliminary supported by the fact that often these shifts in host ecology seem to have occurred between closely related mutillid lineages (e.g. within Mutillinae and within Myrmosinae). Shifts from solitary to social hosts and *vice versa*, as well as from wasp to bee and *vice versa*, are particularly visible. Thus, there could be an “ethological selection” [81] of the hosts.

Despite patterns of preference for hosts with different ecological profiles did not correlated with host average taxonomic distinctness or network-based indices of specialization, it is interesting that the high values of Δ^+^ and the low values of Horn’s index were more often recorded for tribes/subfamilies with higher % of solitary hosts. A larger sample of species and lineages may unveil clearer trends. These trends in turn could be due to the generally greater diversity of available solitary wasps (many subfamilies and genera) than social bees (essentially Halictinae and Apinae in few genera). A reduced diversity of available social bees in the environment may indeed promote a higher host overlap.

Besides the 305 confirmed host associations here analysed in detail, we found in the literature 128 additional potential host associations. A look at these data reveal an overall accordance with the host spectrum defined through the confirmed associations, but also the possibility for some lineages to expand their known host range. For example, Mutillini, Smicromyrmini and Trogaspidiini would add 2–3 subfamilies to their known hosts, but all these potentially new hosts would not affect their overall host ecological profile, suggesting that these could be possible hosts. Similarly, Colletinae ground-nesting bees are listed as potential hosts for Pseudomethocini, which is known to attack other groups of ground-nesting bees, making this addition also possible. On the other hand, some host records seem more unlikely. For example, two wasp species in the subfamily Bembicinae appear among the potential host of Myrmillinae, a lineage completely devoted to attack bees and most often social Halictinae. One suggestion of an ant species as host of *Myrmosa* also seems unlikely. *Dasymutilla* was cited to potentially attack social Halictinae bees and social Polistinae wasps, which are clearly out of its known host range both taxonomically and ecologically; however, this genus is very generalist and some species may be actually specialized on these hosts.

Our results suggest that further studies aimed to understand more in depth the evolution of host use in velvet ants should focus on the following points:

1. attempt to confirm or exclude the currently potential hosts with new observations, and add as much as possible new confirmed hosts especially for mutillid lineages actually still completely unknown (including the entire subfamilies Pseudophotopsidinae and Rhopalomutillinae) and for those still poorly studied (e.g. Kudakrumiini)
2. test the hypothesis that the generally greater taxonomic diversity of available solitary wasps than of social bees in the environment promotes a higher host overlap among mutillids that mainly attack social bees.
3. evaluate if cases of lower specialization at the species level may be hiding specialization at the individual level, as it was observed in generalist cuckoo bees [82]
4. test if chemical insignificance is especially associated with social hosts, since velvet ants have to share the nest with host workers during the invasion [78, 83], or it is a more general feature for mutillids given their overall broad potential to attack taxonomically diverse hosts. The apparent lack of velvet ant species that are specialized to only one may have limited the evolution of precise mimicry [84, 85], though a weaker but still significant chemical mimicry cannot be discarded if all the hosts come from e.g. the same genus [86].
5. build a robust molecular phylogeny of Mutillidae to reconstruct the possible evolutionary shifts in host use across lineages, a phenomenon already reported for other aculeate parasitoids [87]. Host shifts in velvet ants seem to involve especially the host ecological profile. Hence, it is possible that host use evolution responds to the process of “ecological fitting”, which occurs when organisms encounter novel environmental conditions (e.g. a new type of host nest, a new type of host social behaviour) and persist by "fitting" through traits they already possess (e.g. capacity to parasitize concealed immature stages) [88]. For the same reason, species within mutillid lineages sharing a certain host ecological profile could have further segregate hosts by shifting to different host taxa that “fit” the host ecology profile they already exploit.

## Supporting information

S1 Table Complete information of the mutillid-host associations retrieved from the literature, together with their references. The dataset includes species names and classifications of both mutillids and hosts, *plus* the ecological traits of hosts. Binary states for host ecological traits: 0 = carnivorous, ground-nesting, solitary; 1 = herbivorous, aerial-nesting, social. Overall, 305 confirmed (C) host associations (i.e. those used in the analyses) and 128 potential (P) host associations are included.
(DOC)
