# Supplementary material for: A sting affair: A global quantitative exploration of bee, wasp and ant hosts of velvet ants
Source: PLoS One. 2020 Sep 11;15(9):e0238888. doi: 10.1371/journal.pone.0238888 (PMC7485775; doi:10.1371/journal.pone.0238888)
Supplement: S1 Table — The dataset includes species names and classifications of both mutillids and hosts, plus the ecological traits of hosts. Binary states for host ecological traits: 0 = carnivorous, ground-nesting, solitary; 1 = herbivorous, aerial-nesting, social. Overall, 305 confirmed (C) host associations (i.e. those used in the analyses) and 128 potential (P) host associations are included. (DOC) [file pone.0238888.s001.doc]

**Supporting information: S1 Tables**

| **MUTILLIDAE Species** | **HOST Family** | **HOST subfamily** | **Host Species** | **HOST LARVAL DIET** | **HOST NEST TYPE** | **HOST SOCIALITY** | **HOST DATA** | **REFERENCE** |
| --- | --- | --- | --- | --- | --- | --- | --- | --- |
| **Dasylabrinae: Dasylabrini** | | | | | | | | |
| *Dasylabris maura* (Linnaeus, 1758) | Crabronidae | Philanthinae | *Philanthus triangulum* (Fabricius, 1775) | 0 | 0 | 0 | C | Evans & O'Neill (1988) |
| *Dasylabris maura* (Linnaeus, 1758) | Sphecidae | Ammophilinae | *Ammophila heydeni* Dahlbom, 1845 | 0 | 0 | 0 | C | Bogusch (2006), Marí (1943) |
| *Dasylabris maura* (Linnaeus, 1758) | Sphecidae | Sphecinae | *Sphex occitanicus* Lepeletier & Serville, 1828 | 0 | 0 | 0 | C | Bogusch (2006) |
| *Dasylabris maura* (Linnaeus, 1758) | Vespidae | Eumeninae | *Katamenes arbustorum* (Panzer 1799) | 0 | 1 | 0 | C | Lelej (1985) |
| *Dasylabris zimini* Skorikov, 1935 | Crabronidae | Crabroninae | *Tachysphex* sp. | 0 | 0 | 0 | C | Lelej (1985) |
| *Dasylabroides caffra* (Kohl, 1882) | Sphecidae | Ammophilinae | *Ammophila ferrugineipes* Lepeletier, 1845 | 0 | 0 | 0 | C | Gess & Gess (2010) |
| *Dasylabroides caffra* (Kohl, 1882) | Sphecidae | Ammophilinae | *Ammophila insignis* Smith, 1856 | 0 | 0 | 0 | C | Gess & Gess (2010) |
| *Dasylabroides caffra* (Kohl, 1882) | Vespidae | Masarinae | *Ceramius lichtensteinii* (Klug, 1810) | 1 | 0 | 0 | C | Gess & Gess (2010) |
| *Stenomutilla argentata* (Villers, 1789) | Megachilidae | Megachilinae | *Hoplitis fertoni* (Pérez, 1891) | 1 | 1 | 0 | C | Ferton (1908) |
| *Stenomutilla argentata* (Villers, 1789) | Megachilidae | Megachilinae | *Osmia cyanoxantha* Pérez, 1879 | 1 | 0 | 0 | C | Ferton (1914) |
| *Stenomutilla argentata* (Villers, 1789) | Megachilidae | Megachilinae | *Osmia saundersi* Vachal, 1891 | 1 | 0 | 0 | C | Ferton (1921) |
| *Stenomutilla argentata* (Villers, 1789) | Megachilidae | Megachilinae | *Osmia tunensis* (Fabricius, 1787) | 1 | 1 | 0 | C | Ferton (1908) |
| *Stenomutilla argentata* (Villers, 1789) | Vespidae | Eumeninae | *Leptochilus alpestris* (de Saussure, 1856) | 0 | 0 | 0 | C | Marì (1943) |
| *Stenomutilla argentata* (Villers, 1789) | Vespidae | Eumeninae | *Leptochilus mauritanicus* (Lepeletier, 1841) | 0 | 1 | 0 | C | Marì (1943) |
| *Stenomutilla argentata* (Villers, 1789) | Vespidae | Masarinae | *Masaris vespiformis* Fabricius, 1793 | 1 | 0 | 0 | C | Invrea (1964) |
| *Stenomutilla bizonata* (Smith, 1856) | Megachilidae | Megachilinae | *Anthidium contractum* Latreille, 1809 | 1 | 1 | 0 | P | Marì (1943) |
| *Stenomutilla bizonata* (Smith, 1856) | Vespidae | Eumeninae | *Ancistrocerus dusmetiolus* (Strand, 1914) | 0 | 0 | 0 | P | Marì (1943) |
| *Stenomutilla collaris* (Fabricius, 1787) | Megachilidae | Megachilinae | *Hoplitis fertoni* (Pérez, 1891) | 1 | 1 | 0 | C | Bogusch et al. (2020) |
| *Stenomutilla hottentotta* (Fabricius, 1804) | Megachilidae | Megachilinae | *Hoplitis fertoni* (Pérez, 1891) | 1 | 1 | 0 | C | Bogusch et al. (2020) |
| *Tricholabiodes* sp. | Vespidae | Masarinae | *Quartinia* sp. | 1 | 1 | 0 | C | Gess & Gess (2010) |
| **Mutillinae: Mutillini** | | | | | | | | |
| *Barymutilla barbara* Linnaeus, 1758 | Crabronidae | Crabroninae | *Larra anathema* (Rossi, 1790) | 0 | 0 | 0 | P | André (1899-1903) |
| *Ephuta chrysodora* (Perty, 1833) | Apidae | Apinae | *Monoeca haemorrhoidalis* (Smith, 1854) | 1 | 0 | 0 | P | Rocha-Filho & Melo (2011) |
| *Ephuta icema* Casal, 1969 | Pompilidae | Pepsinae | *Auplopus subaurarius* Dreisbach, 1963 | 0 | 1 | 0 | C | Cambra et al. (2017) |
| *Ephuta pauxilla* Bradley, 1916 | Pompilidae | Pepsinae | *Dipogon sayi* Banks, 1941 | 0 | 1 | 0 | C | Cambra et al. (2017), Evans & Yoshimoto (1962) |
| *Ephuta pocinga* (Casal 1970) | Pompilidae | Pepsinae | *Auplopus militaris* (Lynch-Arribalzaga, 1873) | 0 | 1 | 0 | C | Cambra et al. (2017), Zanette et al. (2004) |
| *Ephuta sapuca* Casal, 1968 | Pompilidae | Pepsinae | *Auplopus militaris* (Lynch-Arribalzaga, 1873) | 0 | 1 | 0 | C | Cambra et al. (2017) |
| *Ephuta scrupea* Say, 1836 | Crabronidae | Pemphredoninae | *Diodontus virginianus* (Rohwer, 1917) | 0 | 0 | 0 | C | Krombein(1958) |
| *Ephuta scrupea* Say, 1836 | Pompilidae | Pepsinae | *Phanagenia bombycina* (Cresson, 1867) | 0 | 1 | 0 | C | Schuster (1956) |
| *Ephuta slossonae* (Fox 1899) | Pompilidae | Pompilinae | *Episyron conterminus* (Smith, 1873) | 0 | 0 | 0 | C | Krombein & Norden (1996) |
| *Macromyrme sinuata* (Olivier, 1811) | Apidae | Apinae | *Anthophora* sp. | 1 | 0 | 0 | C | Invrea (1965) |
| *Mutilla aglae* Peringuey, 1898 | Apidae | Xylocopinae | *Ceratina* sp. | 1 | 1 | 0 | C | Peringuey (1898) |
| *Mutilla europaea* Linnaeus,1758 | Apidae | Apinae | *Apis mellifera* Linnaeus, 1758 | 1 | 1 | 1 | C | Brothers et al. (2000), Jordan (1935) |
| *Mutilla europaea* Linnaeus,1758 | Apidae | Apinae | *Bombus argillaceus* (Scopoli, 1763) | 1 | 0 | 1 | C | Hoffer (1886) |
| *Mutilla europaea* Linnaeus,1758 | Apidae | Apinae | *Bombus breviceps* Smith, 1852 | 1 | 0 | 1 | C | Su et al. (2019) |
| *Mutilla europaea* Linnaeus,1758 | Apidae | Apinae | *Bombus confusus* Schenck, 1859 | 1 | 0 | 1 | C | Hoffer (1886) |
| *Mutilla europaea* Linnaeus,1758 | Apidae | Apinae | *Bombus haemorrhoidalis* Smith, 1852 | 1 | 0 | 1 | C | Su et al. (2019) |
| *Mutilla europaea* Linnaeus,1758 | Apidae | Apinae | *Bombus humilis* Illiger 1806 | 1 | 0 | 1 | C | Pouvreau (1973) |
| *Mutilla europaea* Linnaeus,1758 | Apidae | Apinae | *Bombus hypnorum* Linnaeus, 1758 | 1 | 0 | 1 | C | Skorikov (1935) Pouvreau (1973) |
| *Mutilla europaea* Linnaeus,1758 | Apidae | Apinae | *Bombus jonellus* (Kirby,1802) | 1 | 0 | 1 | C | Drewsen (1847) |
| *Mutilla europaea* Linnaeus,1758 | Apidae | Apinae | *Bombus lapidarius* (Linnaeus, 1758) | 1 | 0 | 1 | C | Pouvreau (1973), Jordan (1935), Hoffer (1886), May (1959) |
| *Mutilla europaea* Linnaeus,1758 | Apidae | Apinae | *Bombus lucorum* (Linnaeus, 1761) | 1 | 0 | 1 | C | Pouvreau (1973) |
| *Mutilla europaea* Linnaeus,1758 | Apidae | Apinae | *Bombus mesomelas* Gerstaecker, 1869 | 1 | 0 | 1 | C | Hoffer (1886), Saz (1935) |
| *Mutilla europaea* Linnaeus,1758 | Apidae | Apinae | *Bombus muscorum* (Linnaeus,1758) | 1 | 0 | 1 | C | Erlandsson (1964) |
| *Mutilla europaea* Linnaeus,1758 | Apidae | Apinae | *Bombus pascuorum* (Scopoli, 1763) | 1 | 0 | 1 | C | Hoffer (1886) Forsius (1927) Erlandsson (1964) |
| *Mutilla europaea* Linnaeus,1758 | Apidae | Apinae | *Bombus pomorum* (Panzer, 1805) | 1 | 0 | 1 | C | Hoffer (1886) |
| *Mutilla europaea* Linnaeus,1758 | Apidae | Apinae | *Bombus pratorum* (Linnaeus, 1756) | 1 | 0 | 1 | C | Hoffer (1886) |
| *Mutilla europaea* Linnaeus,1758 | Apidae | Apinae | *Bombus ruderarius* (Müller, 1776) | 1 | 0 | 1 | C | Pouvreau (1973), Dahlbom (1847), Hoffer (1886) |
| *Mutilla europaea* Linnaeus,1758 | Apidae | Apinae | *Bombus sichelii* Radoszkowski, 1859 | 1 | 0 | 1 | C | Móczár (1977) |
| *Mutilla europaea* Linnaeus,1758 | Apidae | Apinae | *Bombus subterraneus* (Linnaeus,1758) | 1 | 0 | 1 | C | Hoffer (1938) |
| *Mutilla europaea* Linnaeus,1758 | Apidae | Apinae | *Bombus sylvarum* (Linnaeus, 1761) | 1 | 0 | 1 | C | Hoffer (1886) |
| *Mutilla europaea* Linnaeus,1758 | Apidae | Apinae | *Bombus terrestris* (Linnaeus, 1758) | 1 | 0 | 1 | C | Pouvreau (1973), Jordan (1935), Hoffer (1886) |
| *Mutilla europaea* Linnaeus,1758 | Apidae | Apinae | *Bombus variabilis* (Cresson, 1872) | 1 | 0 | 1 | C | Hoffer (1886) |
| *Mutilla europaea* Linnaeus,1758 | Apidae | Apinae | *Bombus wurflenii* Radoszkowski, 1859 | 1 | 0 | 1 | C | Hoffer (1886) |
| *Mutilla europaea* Linnaeus,1758 | Vespidae | Polistinae | *Polistes biglumis* (Linnaeus, 1758) | 0 | 1 | 1 | C | Uboni & Lorenzi (2013) |
| *Mutilla europaea* Linnaeus,1758 | Vespidae | Polistinae | *Polistes gallicus* (Linnaeus, 1767) | 0 | 1 | 1 | C | Beljavsky (1935) |
| *Mutilla ferrugata* Fabricius, 1805 | Crabronidae | Philanthinae | *Philanthus* sp. | 0 | 0 | 0 | P | Melander & Brues (1903) |
| *Mutilla lichtensteini* Tournier, 1889 | Megachilidae | Megachilinae | *Anthidium contractum* Latreille, 1809 | 1 | 1 | 0 | C | Tournier (1889) |
| *Mutilla marginata* Baer, 1848 | Apidae | Apinae | *Bombus sylvarum* (Linnaeus, 1761) | 1 | 0 | 1 | C | Hoffer (1886) |
| *Mutilla mikado* Cameron, 1900 | Apidae | Apinae | *Bombus ardens* Smith, 1879 | 1 | 0 | 1 | C | Taniguchi (1955), Katayama (1966), Tsuneki (1972), Sakagamy & Katayama (1977) |
| *Mutilla mikado* Cameron, 1900 | Apidae | Apinae | *Bombus deuteronymus* Schulz, 1906 | 1 | 0 | 1 | C | Katayama et al. (1993) |
| *Mutilla mikado* Cameron, 1900 | Apidae | Apinae | *Bombus diversus* Smith, 1869 | 1 | 0 | 1 | C | Abe (1932), Morimoto et al. (1951), Miyamoto (1959), Katayama (1966), Sakagami & Katayama (1977) |
| *Mutilla mikado* Cameron, 1900 | Apidae | Apinae | *Bombus ignitus* (Smith, 1869) | 1 | 0 | 1 | C | Miyamoto (1963) Sakagami & Katayama (1977) |
| *Mutilla quinquemaculata* Cyrillo, 1787 | Megachilidae | Megachilinae | *Anthidium florenthinum* (Fabricius, 1775) | 1 | 1 | 0 | C | Lelej et al. (2016) |
| *Mutilla quinquemaculata* Cyrillo, 1787 | Megachilidae | Megachilinae | *Megachile* sp. | 1 | 1 | 0 | C | Invrea (1964) |
| *Mutilla quinquemaculata* Cyrillo, 1787 | Megachilidae | Megachilinae | *Osmia tricornis* Latreille, 1811 | 1 | 1 | 0 | C | Marì (1943) |
| *Mutilla saltensis* Radoszkowski, 1885 | Apidae | Apinae | *Bombus armeniacus* Radoszkowski, 1877 | 1 | 0 | 1 | C | Monfared & Shahrza (2010) |
| *Mutilla saltensis* Radoszkowski, 1885 | Apidae | Apinae | *Bombus humilis* Illiger 1806 | 1 | 0 | 1 | C | Radoszkowski (1885) |
| *Ronisia barbara* (Linnaeus, 1758) | Crabronidae | Crabroninae | *Larra anathema* (Rossi, 1790) | 0 | 0 | 0 | C | André (1899-1903) |
| *Ronisia brutia* (Petagna, 1787) | Apidae | Apinae | *Anthophora crinipes* Smith, 1854 | 1 | 0 | 0 | C | Marì (1943) |
| *Ronisia brutia* (Petagna, 1787) | Megachilidae | Megachilinae | *Megachile albisecta* (Klug, 1817) | 1 | 1 | 0 | C | Lelej et al. (2016) |
| *Ronisia brutia* (Petagna, 1787) | Megachilidae | Megachilinae | *Megachile parietina* (Geoffroy, 1785) | 1 | 1 | 0 | C | Bogusch (2006) |
| *Ronisia brutia* (Petagna, 1787) | Vespidae | Polistinae | *Polistes biglumis* (Linnaeus, 1758) | 0 | 1 | 1 | C | De Stefani Perez (1882) |
| *Ronisia brutia* (Petagna, 1787) | Vespidae | Polistinae | *Polistes gallicus* (Linnaeus, 1767) | 0 | 1 | 1 | C | De Stefani Perez (1882) |
| *Ronisia ghiliani* (Spinola, 1843) | Crabronidae | Bembicinae | *Stizus continuus* (Klug, 1835) | 0 | 0 | 0 | P | Giovanetti et al. (2006) |
| *Tropidotilla litoralis* (Petagna, 1787) | Vespidae | Eumeninae | *Paragymnomerus spiricornis* (Spinola, 1808) | 0 | 0 | 0 | C | Amiet (2008) |
| *Tropidotilla litoralis* (Petagna, 1787) | Vespidae | Polistinae | *Polistes biglumis* (Linnaeus, 1758) | 0 | 1 | 1 | C | De Stefani Perez (1882) |
| *Tropidotilla litoralis* (Petagna, 1787) | Vespidae | Polistinae | *Polistes gallicus* (Linnaeus, 1767) | 0 | 1 | 1 | C | De Stefani Perez (1882) |
| **Mutillinae: Smichromyrmini** | | | | | | | | |
| *Ephucilla brevitegula* Okayasu, 2020 | Vespidae | Eumeninae | *Phimenes flavopictus* (Zimmermann, 1931) | 0 | 1 | 0 | C | Okayasu (2020) |
| *Ephucilla ludovica* (Cameron, 1900) | Vespidae | Eumeninae | *Paraleptomenes mephitis* (Cameron, 1901) | 0 | 1 | 0 | C | Krombein(1978) |
| *Ephucilla poonaensis* (Cameron, 1892) | Vespidae | Eumeninae | *Paraleptomenes mephitis* (Cameron, 1901) | 0 | 1 | 0 | C | Krombein(1978) |
| *Mickelomyrme hageni* (Zavattari, 1913) | Halictidae | Halictinae | *Lasioglossum subtropicum* Sakagami, Miyanaga & Maeta 1994 | 1 | 0 | 1 | P | Brothers et al. (2000) |
| *Nemka aurantiaca* (Skorikov, 1935) | Crabronidae | Bembicinae | *Bembecinus acanthomerus* (Morice, 1911) | 0 | 0 | 0 | P | Lelej (1995) |
| *Nemka viduata* (Pallas) | Crabronidae | Bembicinae | *Bembecinus tridens* (Fabricius, 1781) | 0 | 0 | 0 | C | Tormos et al. (2009) |
| *Nemka viduata* (Pallas) | Crabronidae | Bembicinae | *Bembix olivacea* Fabricius, 1787 | 0 | 0 | 0 | C | Templado (1958) |
| *Nemka viduata* (Pallas) | Crabronidae | Bembicinae | *Gorytes* sp. | 0 | 0 | 0 | C | Bogusch (2006) |
| *Nemka viduata* (Pallas) | Crabronidae | Bembicinae | *Stizus continuus* (Klug, 1835) | 0 | 0 | 0 | C | Polidori et al. (2010) |
| *Nemka viduata* (Pallas) | Crabronidae | Crabroninae | *Larra anathema* (Rossi, 1790) | 0 | 0 | 0 | P | Bogusch (2006) |
| *Nemka viduata* (Pallas) | Megachilidae | Megachilinae | *Megachile leucomalla* Gerstäcker, 1869 | 1 | 1 | 0 | C | Lelej (1995) |
| *Nemka viduata* (Pallas) | Melittidae | Dasypodainae | *Dasypoda hirtipes* (Fabricius, 1793) | 1 | 0 | 0 | C | Lichtenstein (1878) Bogusch (2006) |
| *Nemka yasumatsui* (Mickel, 1936) | Crabronidae | Bembicinae | *Bembecinus hungaricus* (Frivaldszky, 1876) | 0 | 0 | 0 | P | Okayasu (2020) |
| *Nemka yasumatsui* (Mickel, 1936) | Crabronidae | Bembicinae | *Bembix niponica* Smith, 1873 | 0 | 0 | 0 | C | Okayasu (2020) |
| *Nemka yasumatsui* (Mickel, 1936) | Crabronidae | Bembicinae | *Stizus perrisi* Dufour, 1838 | 0 | 0 | 0 | P | Okayasu (2020) |
| *Physetopoda daghestanica* (Radoszkowski, 1885) | Crabronidae | Bembicinae | *Bembecinus tridens* (Fabricius, 1781) | 0 | 0 | 0 | C | Lelej (1985) |
| *Physetopoda fumigata* (Turner 1911) | Crabronidae | Crabroninae | *Krombeinictus nordenae* Leclercq, 1996 | 1 | 1 | 0 | C | Krombein (1999) |
| *Promecilla decora* Smith, 1879 | Crabronidae | Crabroninae | *Pison argentatum* Shuckard, 1838 | 0 | 1 | 0 | C | Pagden (1949) Taylor et al. (2019) |
| *Promecilla primana* Skorikov, 1935 | Sphecidae | Sceliphrinae | *Sceliphron* sp. | 0 | 1 | 0 | C | Lelej (1985) |
| *Promecilla regia* (Smith, 1855) | Vespidae | Eumeninae | *Delta campaniforme* (Fabricius, 1775) | 0 | 1 | 0 | C | Dutt (1912) |
| *Promecilla regia* (Smith, 1855) | Vespidae | Eumeninae | *Delta conoideum* Gmelin, 1790 | 0 | 0 | 0 | C | Dutt (1912) |
| *Promecilla unicingulata* Bischoff, 1921 | Crabronidae | Crabroninae | *Dasyproctus bipunctatus* Lepeletier de Saint Fargeauand Brullé, 1835 | 0 | 1 | 0 | C | Carpenter (1942) |
| *Promecilla yerburghi* (Cameron, 1892) | Vespidae | Eumeninae | *Paraleptomenes humbertianus* (de Saussure, 1867) | 0 | 1 | 0 | C | Krombein(1978) |
| *Promecilla yerburghi* (Cameron, 1892) | Vespidae | Eumeninae | *Paraleptomenes mephitis* (Cameron, 1901) | 0 | 1 | 0 | C | Krombein(1978) |
| *Smicromyrme lewisi* Mickel, 1935 | Colletidae | Colletinae | *Colletes* sp. | 1 | 0 | 0 | P | Okayasu (2020) |
| *Smicromyrme lewisi* Mickel, 1935 | Colletidae | Colletinae | *Hylaeus* sp. | 1 | 1 | 0 | P | Okayasu (2020) |
| *Smicromyrme lewisi* Mickel, 1935 | Crabronidae | Bembicinae | *Bembix niponica* Smith, 1873 | 0 | 0 | 0 | C | Okayasu (2020) |
| *Smicromyrme lewisi* Mickel, 1935 | Crabronidae | Bembicinae | *Gorytes tricinctus* (Pérez, 1905) | 0 | 0 | 0 | C | Okayasu (2020) |
| *Smicromyrme lewisi* Mickel, 1935 | Crabronidae | Crabroninae | *Oxybelus bipunctatus* Olivier, 1812 | 0 | 0 | 0 | P | Okayasu (2020) |
| *Smicromyrme lewisi* Mickel, 1935 | Crabronidae | Crabroninae | *Tachysphex nigricolor* (Dalla Torre, 1897) | 0 | 0 | 0 | C | Okayasu (2020) |
| *Smicromyrme lewisi* Mickel, 1935 | Crabronidae | Philanthinae | *Cerceris hortivaga* Kohl, 1880 | 0 | 0 | 0 | C | Okayasu (2020) |
| *Smicromyrme lewisi* Mickel, 1935 | Crabronidae | Philanthinae | *Cerceris* sp. | 0 | 0 | 0 | P | Lelej et al. (2001) |
| *Smicromyrme nigriceps* Nonveiller, 1959 | Crabronidae | Bembicinae | *Bembecinus tridens* (Fabricius, 1781) | 0 | 0 | 0 | C | Lelej et al. (2016) |
| *Smicromyrme rufipes* (Fabricius, 1787) | Apidae | Apinae | *Bombus hypnorum* Linnaeus, 1758 | 1 | 0 | 1 | C | Sichel & Radoszkowksy (1869) |
| *Smicromyrme rufipes* (Fabricius, 1787) | Apidae | Apinae | *Bombus lapidarius* (Linnaeus, 1758) | 1 | 0 | 1 | P | Sichel & Radoszkowksy (1869) |
| *Smicromyrme rufipes* (Fabricius, 1787) | Crabronidae | Astatinae | *Astata boops* (Schrank,1781) | 0 | 0 | 0 | C | Lelej (1985) |
| *Smicromyrme rufipes* (Fabricius, 1787) | Crabronidae | Bembicinae | *Bembecinus tridens* (Fabricius, 1781) | 0 | 0 | 0 | C | Tormos et al. (2009) |
| *Smicromyrme rufipes* (Fabricius, 1787) | Crabronidae | Bembicinae | *Bembix rostrata* (Linnaeus, 1758) | 0 | 0 | 0 | P | Polidori et al. (2012) |
| *Smicromyrme rufipes* (Fabricius, 1787) | Crabronidae | Bembicinae | *Hoplisoides latifrons* (Spinola, 1808) | 0 | 0 | 0 | C | Gogala (2017) |
| *Smicromyrme rufipes* (Fabricius, 1787) | Crabronidae | Crabroninae | *Crossocerus wesmaeli* (Vander Linden, 1829) | 0 | 0 | 0 | C | Lelej (1985) |
| *Smicromyrme rufipes* (Fabricius, 1787) | Crabronidae | Crabroninae | *Miscophus spurius* (Dahlbom, 1832) | 0 | 0 | 0 | C | Bogusch (2006) |
| *Smicromyrme rufipes* (Fabricius, 1787) | Crabronidae | Crabroninae | *Oxybelus bipunctatus* Olivier, 1812 | 0 | 0 | 0 | P | Bogusch (2006) |
| *Smicromyrme rufipes* (Fabricius, 1787) | Crabronidae | Crabroninae | *Oxybelus uniglumis* (Linnaeus, 1758) | 0 | 0 | 0 | C | Kieffer (1902), Bogusch (2006), Crèvecoeur (1930), Marì (1943) |
| *Smicromyrme rufipes* (Fabricius, 1787) | Crabronidae | Crabroninae | *Palarus variegatus* (Fabricius, 1781) | 0 | 0 | 0 | C | Gogala (2017) |
| *Smicromyrme rufipes* (Fabricius, 1787) | Crabronidae | Crabroninae | *Tachysphex* sp. | 0 | 0 | 0 | C | Gogala (2017) |
| *Smicromyrme rufipes* (Fabricius, 1787) | Crabronidae | Crabroninae | *Tracheliodes quinquenotatus* (Jurine, 1807) | 0 | 0 | 0 | C | Grandi (1961) |
| *Smicromyrme rufipes* (Fabricius, 1787) | Crabronidae | Philanthinae | *Cerceris arenaria* (Linnaeus, 1758) | 0 | 0 | 0 | C | Lelej (1985) |
| *Smicromyrme rufipes* (Fabricius, 1787) | Pompilidae | Pompilinae | *Evagetes laboriosus* Ferton, 1897 | 0 | 0 | 0 | P | Bouvier (1922) |
| *Smicromyrme sicanus* (De Stefani, 1887) | Crabronidae | Crabroninae | *Miscophus bicolor* Jurine,1807 | 0 | 1 | 0 | C | Lelej (1985) |
| *Smicromyrme sicanus* (De Stefani, 1887) | Crabronidae | Crabroninae | *Nitela spinolae* Latreille, 1809 | 0 | 0 | 0 | C | Lelej (1985) |
| *Smicromyrme sicanus* (De Stefani, 1887) | Crabronidae | Crabroninae | *Tachysphex pompiliformis* (Panzer,1805) | 0 | 0 | 0 | C | Lelej (1985) |
| *Smicromyrme sicanus* (De Stefani, 1887) | Crabronidae | Pemphredoninae | *Diodontus minutus* (Fabricius, 1793) | 0 | 0 | 0 | C | Lelej (1985) |
| *Smicromyrme sicanus* (De Stefani, 1887) | Crabronidae | Philanthinae | *Cerceris arenaria* (Linnaeus, 1758) | 0 | 0 | 0 | C | Lelej (1985) |
| *Smycromyrme partitus* (Klug, 1835) | Crabronidae | Philanthinae | *Philanthus triangulum* (Fabricius, 1775) | 0 | 0 | 0 | C | Invrea (1941) |
| **Mutillinae: Trogaspidiini** | | | | | | | | |
| *Dolichomutilla sycorax* (Smith 1855) | Sphecidae | Sceliphrinae | *Sceliphron spirifex* (Linnaeus, 1758) | 0 | 1 | 0 | P | Bayliss & Brothers (2001) |
| *Krombeinidia oglana* (Cameron, 1900) | Sphecidae | Sceliphrinae | *Sceliphron madraspatanum* (Fabricius, 1781) | 0 | 1 | 0 | C | Dutt (1912) |
| *Pagdenidia hymalajensis* (Radoszkowski, 1885) | Tiphiidae | Tiphiinae | *Tiphia matura* Allen & Jaynes, 1930 | 0 | 0 | 0 | P | Thompson (1972) |
| *Timulla centroamericana* (Dalla Torre, 1897) | Crabronidae | Crabroninae | *Liris* sp. near *argenticauda* (Cameron, 1889) | 0 | 0 | 0 | C | Quintero & Cambra (1996) |
| *Timulla cordillera* Mickel, 1938 | Sphecidae | Sphecinae | *Sphex ichneumoneus* (Linnaeus, 1758) | 0 | 0 | 0 | P | Quintero & Cambra (1996) |
| *Timulla eriphyla* Mickel, 1938 | Crabronidae | Crabroninae | *Tachysphex blatticidus* Williams, 1941 | 0 | 0 | 0 | C | Callan (1942) |
| *Timulla ferrugata* (Fabricius, 1804) | Vespidae | Eumeninae | *Eumenes fraternus* Say, 1824 | 0 | 1 | 0 | C | Fattig (1943) |
| *Timulla intermissa* Mickel, 1938 | Apidae | Apinae | *Exomalopsis fulvofasciata* (Smith, 1879) | 1 | 0 | 0 | P | Aranda & Graciolli (2013) |
| *Timulla leona* (Blake, 1871) | Crabronidae | Bembicinae | *Gorytes canaliculatus* Packard, 1867 | 0 | 0 | 0 | C | Evans (1966), Quintero & Cambra (1996) |
| *Timulla terminalis* (Gerstaecker, 1874) | Apidae | Apinae | *Exomalopsis fulvofasciata* (Smith, 1879) | 1 | 0 | 0 | P | Aranda & Graciolli (2013) |
| *Timulla vagans* (Fabricius, 1798) | Apidae | Apinae | *Svastra obliqua* (Say, 1837) | 1 | 0 | 0 | P | Rozen (1964) |
| *Timulla vagans* (Fabricius, 1798) | Halictidae | Halictinae | *Lasioglossum pruinosus* (Robertson, 1892) | 1 | 0 | 1 | P | Melander & Brues (1903) |
| *Trogaspidia castellana* (Mercet, 1903) | Scolidae | Campsomerinae | *Campsomeris annulata* (Fabricius, 1793) | 0 | 0 | 0 | C | thompson (1972), Fabricius (1973) |
| *Trogaspidia castellana* (Mercet, 1903) | Scolidae | Campsomerinae | *Campsomeris asiatica* (Saussure, 1858) | 0 | 0 | 0 | C | Thompson (1972) |
| *Trogaspidia castellana* (Mercet, 1903) | Scolidae | Campsomerinae | *Campsomeris aureicollis* Lepeletier, 1935 | 0 | 0 | 0 | C | Smith (1855), Thompson (1972) |
| *Trogaspidia castellana* (Mercet, 1903) | Scolidae | Campsomerinae | *Campsomeris auruluenta* (Smith, 1855) | 0 | 0 | 0 | C | Smith (1855), Thompson (1972) |
| *Trogaspidia castellana* (Mercet, 1903) | Scolidae | Campsomerinae | *Campsomeris luctuosa* Smith, 1864 | 0 | 0 | 0 | C | Smith (1855), Thompson (1972) |
| *Trogaspidia castellana* (Mercet, 1903) | Scolidae | Campsomerinae | *Campsomeris marginela* (Smith, 1855) | 0 | 0 | 0 | C | Smith (1855), Thompson (1972) |
| *Trogaspidia castellana* (Mercet, 1903) | Scolidae | Campsomerinae | *Campsomeris reticulata* Cameron, 1892 | 0 | 0 | 0 | C | Cameron (1892), Thompson (1972) |
| *Trogaspidia castellana* (Mercet, 1903) | Scolidae | Scoliinae | *Liacos analis* (Fabricius, 1804) | 0 | 0 | 0 | C | Fabricius (1804) |
| *Trogaspidia castellana* (Mercet, 1903) | Scolidae | Scoliinae | *Megascolia scutellaris* (Gribodo, 1893) | 0 | 0 | 0 | C | Lelej (2005) |
| *Trogaspidia castellana* (Mercet, 1903) | Scolidae | Scoliinae | *Scolia* sp. | 0 | 0 | 0 | P | Lopez (1931) |
| *Trogaspidia castellana* (Mercet, 1903) | Tiphiidae | Tiphiinae | *Tiphia lucida* Crawford, 1910 | 0 | 0 | 0 | C | Williams (1919) |
| *Trogaspidia castellana* (Mercet, 1903) | Tiphiidae | Tiphiinae | *Tiphia segregata* Crawford, 1910 | 0 | 0 | 0 | C | Thompson (1944), thompson (1972) |
| **Myrmillinae** | | | | | | | | |
| *Bethsmyrmilla alticola* Krombein & Lelej, 1999 | Halictidae | Halictinae | *Lasioglossum alphenum* (Cameron, 1897) | 1 | 0 | 0 | C | Krombein & Lelej (1999) |
| *Bidecoloratilla leopoldina* (Invrea, 1955) | Crabronidae | Bembicinae | *Bembix merceti* Parker, 1929 | 0 | 0 | 0 | P | Asís et al. (2004) |
| *Bischoffitilla ardescens* (Smith, 1873) | Halictidae | Halictinae | *Lasioglossum sakagamii* Ebmer, 1978 | 1 | 0 | 1 | C | Lelej (1985) |
| *Blakeius bipunctatus* (Latreille, 1792) | Halictidae | Halictinae | *Halictus cochlearitarsis* Dours, 1872 | 1 | 0 | 1 | P | Knerer (1973) |
| *Blakeius bipunctatus* (Latreille, 1792) | Halictidae | Halictinae | *Halictus fulvipes* (Klug, 1817) | 1 | 0 | 1 | P | Knerer (1973) |
| *Blakeius bipunctatus* (Latreille, 1792) | Halictidae | Halictinae | *Halictus resurgens* Nurse, 1903 | 1 | 0 | 1 | P | Knerer (1973) |
| *Blakeius bipunctatus* (Latreille, 1792) | Halictidae | Halictinae | *Lasioglossum glabriusculum* (Morawitz, 1872) | 1 | 0 | 1 | P | Knerer (1973) |
| *Blakeius bipunctatus* (Latreille, 1792) | Halictidae | Halictinae | *Lasioglossum malachurum* (Kirby, 1802) | 1 | 0 | 1 | C | Ferton (1898), Knerer (1973) |
| *Blakeius bipunctatus* (Latreille, 1792) | Halictidae | Halictinae | *Lasioglossum nigripes* (Lepeletier, 1841) | 1 | 0 | 1 | C | Knerer & Plateaux-Quénu (1970) |
| *Myrmilla calva* (Villers, 1789) | Crabronidae | Bembicinae | *Stizus perrisi* Dufour, 1838 | 0 | 0 | 0 | P | Asís et al. (1991) |
| *Myrmilla calva* (Villers, 1789) | Halictidae | Halictinae | *Lasioglossum calceatum* (Scopoli,1763) | 1 | 0 | 1 | C | Bogusch (2006), Sichel (1852) |
| *Myrmilla calva* (Villers, 1789) | Halictidae | Halictinae | *Lasioglossum malachurum* (Kirby, 1802) | 1 | 0 | 1 | C | Knerer (1973), Bogush (2006) |
| *Myrmilla calva* (Villers, 1789) | Halictidae | Halictinae | *Lasioglossum marginatum* (Brullé, 1832) | 1 | 0 | 1 | C | Bogusch (2006) |
| *Myrmilla calva* (Villers, 1789) | Halictidae | Halictinae | *Lasioglossum morio* (Fabricius, 1793) | 1 | 0 | 1 | C | Knerer (1973), Bogush (2006), Sichel (1852) |
| *Myrmilla capitata* (Lucas, 1849) | Halictidae | Halictinae | *Halictus scabiosae* (Rossi, 1790) | 1 | 0 | 1 | P | De Stefani Perez (1886) |
| *Myrmilla capitata* (Lucas, 1849) | Halictidae | Halictinae | *Lasioglossum malachurum* (Kirby, 1802) | 1 | 0 | 1 | C | Ferton (1898) Polidori et al. (2009) |
| *Myrmilla capitata* (Lucas, 1849) | Halictidae | Halictinae | *Lasioglossum nigripes* (Lepeletier, 1841) | 1 | 0 | 1 | P | De Stefani Perez (1886) |
| *Myrmilla capitata* (Lucas, 1849) | Megachilidae | Megachilinae | *Osmia metallica* Lucas, 1849 | 1 | 1 | 0 | C | Sichel & Radoszkowksy (1869) |
| *Myrmilla erythrocephala (*Latreille, 1792) | Halictidae | Halictinae | *Halictus scabiosae* (Rossi, 1790) | 0 | 1 | 1 | C | Present study |
| *Myrmilla erythrocephala (*Latreille, 1792) | Halictidae | Halictinae | *Halictus sexcinctus* (Fabricius, 1775) | 1 | 0 | 1 | P | Brothers et al. (2000) |
| *Myrmilla mutica* (Andre, 1903) | Halictidae | Halictinae | *Halictus scabiosae* (Rossi, 1790) | 1 | 0 | 1 | P | Gogala (1991) |
| *Sigilla dorsata* (Fabricius, 1798) | Halictidae | Halictinae | *Lasioglossum malachurum* (Kirby, 1802) | 1 | 0 | 1 | P | Knerer (1973) |
| *Sigilla dorsata* (Fabricius, 1798) | Halictidae | Halictinae | *Lasioglossum nigripes* (Lepeletier, 1841) | 1 | 0 | 1 | C | Knerer & Plateaux-Quénu (1970) |
| **Myrmosinae: Kudakrumiini** | | | | | | | | |
| *Myrmosula parvula* (Fox, 1893) | Halictidae | Halictinae | *Augochlorella persimilis* (Viereck, 1910) | 1 | 0 | 1 | P | Krombein(1979), Macgown (2006) |
| *Myrmosula parvula* (Fox, 1893) | Halictidae | Halictinae | *Augochlorella striata* (Provancher, 1888) | 1 | 0 | 1 | P | Macgown (2006) |
| *Myrmosula parvula* (Fox, 1893) | Halictidae | Halictinae | *Lasioglossum imitatum* (Smith, 1853) | 1 | 0 | 1 | C | Macgown (2006), Brothers (1978) |
| *Myrmosula parvula* (Fox, 1893) | Halictidae | Halictinae | *Lasioglossum zephyrum* (Smith, 1853) | 1 | 0 | 1 | C | Brothers (1978) |
| **Myrmosinae: Myrmosini** | | | | | | | | |
| *Myrmosa atra* Panzer, 1801 | Crabronidae | Crabroninae | *Crabro peltarius* (Schreber,1784) | 0 | 0 | 0 | C | Bogusch (2006) |
| *Myrmosa atra* Panzer, 1801 | Crabronidae | Crabroninae | *Crossocerus palmipes (Linnaeus)* | 0 | 0 | 0 | C | Bogusch (2006) |
| *Myrmosa atra* Panzer, 1801 | Crabronidae | Crabroninae | *Crossocerus wesmaeli* (Vander Linden, 1829) | 0 | 0 | 0 | C | Bogusch (2006) |
| *Myrmosa atra* Panzer, 1801 | Crabronidae | Crabroninae | *Lindenius albilabris* (Fabricius, 1793) | 0 | 0 | 0 | C | Bogusch (2006) |
| *Myrmosa atra* Panzer, 1801 | Crabronidae | Crabroninae | *Lindenius panzeri* (Vander Linden, 1829) | 0 | 0 | 0 | C | Bogusch (2006) |
| *Myrmosa atra* Panzer, 1801 | Crabronidae | Crabroninae | *Oxybelus uniglumis* (Linnaeus, 1758) | 0 | 0 | 0 | P | Marì (1943) |
| *Myrmosa atra* Panzer, 1801 | Crabronidae | Pemphredoninae | *Diodontus tristis* (Vander Linden, 1829) | 0 | 0 | 0 | C | Bogusch (2006) |
| *Myrmosa atra* Panzer, 1801 | Halictidae | Halictinae | *Lasioglossum fratellum* (Pérez, 1903) | 1 | 0 | 1 | P | Field (1996) |
| *Myrmosa atra* Panzer, 1801 | Halictidae | Halictinae | *Lasioglossum nigripes* (Lepeletier, 1841) | 1 | 0 | 1 | P | De Stefani Perez (1886), Brothers et al. (2000) |
| *Myrmosa unicolor* Say, 1824 | Crabronidae | Crabroninae | *Lindenius columbianus* (Kohl, 1892) | 0 | 0 | 0 | P | Macgown (2006) |
| *Myrmosa unicolor* Say, 1824 | Formicidae | Myrmicinae | *Myrmica scabrinodis* Nylander, 1846 | 0 | 0 | 1 | P | Weber (1934) |
| *Myrmosa unicolor* Say, 1824 | Halictidae | Halictinae | *Lasioglossum imitatum* (Smith, 1853) | 1 | 0 | 1 | C | Macgown (2006) |
| *Myrmosa unicolor* Say, 1824 | Halictidae | Halictinae | *Lasioglossum pruinosus* (Robertson, 1892) | 1 | 0 | 1 | P | Macgown (2006) |
| *Paramyrmosa brunnipes* (Lepeletier, 1845) | Crabronidae | Crabroninae | *Crabro peltarius* (Schreber,1784) | 0 | 0 | 0 | C | Bogusch (2006) |
| *Paramyrmosa brunnipes* (Lepeletier, 1845) | Crabronidae | Philanthinae | *Cerceris rybyensis* (Linnaeus,1771) | 0 | 0 | 0 | C | Bogusch (2006) |
| *Paramyrmosa brunnipes* (Lepeletier, 1845) | Halictidae | Halictinae | *Lasioglossum majus* (Nylander, 1852) | 1 | 0 | 0 | C | Bogusch (2006) |
| *Paramyrmosa brunnipes* (Lepeletier, 1845) | Halictidae | Halictinae | *Lasioglossum malachurum* (Kirby, 1802) | 1 | 0 | 1 | P | Brothers et al. (2000) |
| *Paramyrmosa brunnipes* (Lepeletier, 1845) | Halictidae | Halictinae | *Lasioglossum morio* (Fabricius, 1793) | 1 | 0 | 1 | C | Bogusch (2006) |
| *Paramyrmosa brunnipes* (Lepeletier, 1845) | Halictidae | Halictinae | *Lasioglossum pauxillum* (Schenck, 1853) | 1 | 0 | 1 | C | Bogusch (2006) |
| **Sphaeropthalminae: Dasymutillini** | | | | | | | | |
| *Aglaotilla chalcea* Taylor, Murphy, Hitchen & Brothers, 2019 | Crabronidae | Crabroninae | *Aulacophilinus mandibulatus* (Turner, 1908) | 0 | 1 | 0 | C | Taylor et al. (2019) |
| *Aglaotilla chalcea* Taylor, Murphy, Hitchen & Brothers, 2019 | Crabronidae | Crabroninae | *Pison* cf. *inconspicuum*Turner, 1916 | 0 | 1 | 0 | C | Taylor et al. (2019) |
| *Aglaotilla chalcea* Taylor, Murphy, Hitchen & Brothers, 2019 | Crabronidae | Crabroninae | *Pison* cf. *rufipes* Shuckard, 1838 | 0 | 1 | 0 | C | Taylor et al. (2019) |
| *Aglaotilla chalcea* Taylor, Murphy, Hitchen & Brothers, 2019 | Crabronidae | Crabroninae | *Pison fenestratum* Smith, 1869 | 0 | 1 | 0 | C | Taylor et al. (2019) |
| *Aglaotilla chalcea* Taylor, Murphy, Hitchen & Brothers, 2019 | Crabronidae | Crabroninae | *Pison strenuum* Turner, 1916 | 0 | 1 | 0 | C | Taylor et al. (2019) |
| *Aglaotilla chalcea* Taylor, Murphy, Hitchen & Brothers, 2019 | Crabronidae | Crabroninae | *Pison tibiale* Smith, 1869 | 0 | 1 | 0 | C | Taylor et al. (2019) |
| *Aglaotilla chalcea* Taylor, Murphy, Hitchen & Brothers, 2019 | Vespidae | Eumeninae | *Paralastor debilis* Perkins, 1914 | 0 | 1 | 0 | C | Taylor et al. (2019) |
| *Aglaotilla ignita* (Smith, 1855) | Crabronidae | Crabroninae | *Pison spinolae* Shuckard, 1838 | 0 | 1 | 0 | C | Brothers (1984) |
| *Aglaotilla lathronymphos* Taylor, Murphy, Hitchen & Brothers, 2019 | Crabronidae | Crabroninae | *Pison* cf. *Inconspicuum* Turner, 1916 | 0 | 1 | 0 | C | Taylor et al. (2019) |
| *Aglaotilla lathronymphos* Taylor, Murphy, Hitchen & Brothers, 2019 | Crabronidae | Crabroninae | *Pison* cf. *rufipes* Shuckard, 1838 | 0 | 1 | 0 | C | Taylor et al. (2019) |
| *Aglaotilla lathronymphos* Taylor, Murphy, Hitchen & Brothers, 2019 | Crabronidae | Crabroninae | *Pison fenestratum* Smith, 1869 | 0 | 1 | 0 | C | Taylor et al. (2019) |
| *Aglaotilla lathronymphos* Taylor, Murphy, Hitchen & Brothers, 2019 | Crabronidae | Crabroninae | *Pison* sp. | 0 | 1 | 0 | C | Taylor et al. (2019) |
| *Aglaotilla lathronymphos* Taylor, Murphy, Hitchen & Brothers, 2019 | Crabronidae | Crabroninae | *Pison strenuum* Turner, 1916 | 0 | 1 | 0 | C | Taylor et al. (2019) |
| *Aglaotilla micra* Taylor, Murphy, Hitchen & Brothers, 2019 | Crabronidae | Crabroninae | *Pison* sp. near *fenestratum* Smith, 1869 | 0 | 1 | 0 | C | Taylor et al. (2019) |
| *Aglaotilla schadophaga* Taylor, Murphy, Hitchen & Brothers, 2019 | Megachilidae | Megachilinae | *Megachile oblonga* Smith, 1879 | 1 | 1 | 0 | C | Taylor et al. (2019) |
| *Aglaotilla schadophaga* Taylor, Murphy, Hitchen & Brothers, 2019 | Megachilidae | Megachilinae | *Megachile* sp. | 1 | 1 | 0 | C | Taylor et al. (2019) |
| *Aglaotilla schadophaga* Taylor, Murphy, Hitchen & Brothers, 2019 | Megachilidae | Megachilinae | *Megachile tosticauda* Cockerell, 1912 | 1 | 1 | 0 | C | Taylor et al. (2019) |
| *Aglaotilla submetallescens* (Turner, 1916) | Vespidae | Eumeninae | *Abispa* sp. | 0 | 1 | 0 | C | Brothers (1984) |
| *Dasymutilla aequatorialis* (André, 1906) | Apidae | Apinae | *Melitoma* sp. | 1 | 0 | 0 | C | André (1906) |
| *Dasymutilla araneoides* (Smith, 1862) | Apidae | Apinae | *Centris flavofasciata* Friese, 1899 | 1 | 0 | 0 | P | Vinson et al. (1987) |
| *Dasymutilla araneoides* (Smith, 1862) | Crabronidae | Bembicinae | *Microbembex monodonata* (Say, 1824) | 0 | 0 | 0 | P | Cambra (2018) |
| *Dasymutilla asopus* (Cresson 1865) | Megachilidae | Megachilinae | *Dianthidium curvatum* (Smith, 1854) | 1 | 0 | 0 | C | Fischer (1951) |
| *Dasymutilla asopus* (Cresson 1865) | Megachilidae | Megachilinae | *Dianthidium* sp. | 1 | 0 | 0 | C | Mickel (1928) |
| *Dasymutilla bioculata* (Cresson 1865) | Crabronidae | Bembicinae | *Bembecinius quinquespinosus* (Say, 1823) | 0 | 0 | 0 | C | Mickel (1924b) |
| *Dasymutilla bioculata* (Cresson 1865) | Crabronidae | Bembicinae | *Bembix americana* Fabricius, 1793 | 0 | 0 | 0 | C | Cottrell (1936), Williams & Pitts (2013) |
| *Dasymutilla bioculata* (Cresson 1865) | Crabronidae | Bembicinae | *Bembix amoena* Handlirsch, 1893 | 0 | 0 | 0 | C | Evans (1966), Williams & Pitts (2013) |
| *Dasymutilla bioculata* (Cresson 1865) | Crabronidae | Bembicinae | *Bembix pallidipicta* Smith, 1873 | 0 | 0 | 0 | C | Brothers (1972) |
| *Dasymutilla bioculata* (Cresson 1865) | Crabronidae | Bembicinae | *Bembix sayi* Cresson, 1865 | 0 | 0 | 0 | P | Manley & Deyrup (1989) |
| *Dasymutilla bioculata* (Cresson 1865) | Crabronidae | Bembicinae | *Bembix texana* Cresson, 1873 | 0 | 0 | 0 | C | Evans & O'Neill (2007), Williams & Pitts (2013) |
| *Dasymutilla bioculata* (Cresson 1865) | Crabronidae | Bembicinae | *Bicyrtes quadrifasciata* (Say, 1824) | 0 | 0 | 0 | P | Krombein(1964) |
| *Dasymutilla bioculata* (Cresson 1865) | Crabronidae | Bembicinae | *Microbembex monodonata* (Say, 1824) | 0 | 0 | 0 | C | Deyrup & Manley (1986) |
| *Dasymutilla bioculata* (Cresson 1865) | Crabronidae | Philanthinae | *Philanthus bicinctus* (Mickel, 1916) | 0 | 0 | 0 | P | Evans & O'Neill (1988) |
| *Dasymutilla bioculata* (Cresson 1865) | Pompilidae | Pompilinae | *Anoplius marginalis* (Banks, 1910) | 0 | 0 | 0 | C | Krombein (1964) |
| *Dasymutilla canella* (Blake, 1871) | Halictidae | Halictinae | *Lasioglossum pruinosus* (Robertson, 1892) | 1 | 0 | 1 | P | Melander & Brues (1903) |
| *Dasymutilla coccineohirta* (Blake, 1871) | Crabronidae | Philanthinae | *Cerceris californica* Cresson, 1865 | 0 | 0 | 0 | C | Linsley & MacSwain (1956) |
| *Dasymutilla foxi* (Cockerell, 1894) | Apidae | Apinae | *Diadasia rinconis* Cockerell, 1897 | 1 | 0 | 0 | C | Schmidt & Buchmann (1986) |
| *Dasymutilla gloriosa* (Saussure 1878) | Crabronidae | Bembicinae | *Microbembex nigrifrons* (Provancher, 1888) | 0 | 0 | 0 | C | Duncan & Lighton (1997), Kombrein (1979) |
| *Dasymutilla jalisco* Manley 2003 | Apidae | Apinae | *Diadasia knabiana* Cockerell, 1917 | 1 | 0 | 0 | C | Manley (2003) |
| *Dasymutilla klugii* (Gray, 1832) | Crabronidae | Bembicinae | *Sphecius grandis* (Say, 1823) | 0 | 0 | 0 | P | Bradley (1920) |
| *Dasymutilla klugii* (Gray, 1832) | Sphecidae | Chloriontinae | *Chlorion cyaneum* Dahlbom, 1843 | 0 | 0 | 0 | P | Hook (2004) |
| *Dasymutilla militaris* (Smith 1855) | Crabronidae | Philanthinae | *Philanthus banabacoa* Alayo Dalmau, 1968 | 0 | 0 | 0 | P | Sanchez & Genaro (1992) |
| *Dasymutilla nigripes* (Fabricius, 1787) | Crabronidae | Philanthinae | *Cerceris flavofasciata* Smith, 1908 | 0 | 0 | 0 | C | Krombein(1959) |
| *Dasymutilla nigripes* (Fabricius, 1787) | Crabronidae | Philanthinae | *Philanthus gibbosus* (Fabricius, 1775) | 0 | 0 | 0 | P | Evans (1974) |
| *Dasymutilla nigripes* (Fabricius, 1787) | Crabronidae | Philanthinae | *Philanthus lepidus* Cresson, 1865 | 0 | 0 | 0 | P | Evans (1964) |
| *Dasymutilla nigripes* (Fabricius, 1787) | Crabronidae | Philanthinae | *Philanthus sanbornii* Cresson, 1865 | 0 | 0 | 0 | C | Evans & O'Neill (1988) |
| *Dasymutilla occidentalis* (Linnaeus, 1758) | Apidae | Apinae | *Bombus fraternus* (Smith, 1854) | 1 | 0 | 1 | C | Fattig (1943) |
| *Dasymutilla occidentalis* (Linnaeus, 1758) | Apidae | Apinae | *Apis mellifera* Linnaeus, 1758 | 1 | 1 | 1 | P | Brothers et al. (2000), Bryant (1870) |
| *Dasymutilla occidentalis* (Linnaeus, 1758) | Crabronidae | Bembicinae | *Sphecius speciosus* (Drury, 1773) | 0 | 0 | 0 | P | Byers (1991) |
| *Dasymutilla occidentalis* (Linnaeus, 1758) | Crabronidae | Bembicinae | *Stictia carolina* (Fabricius, 1793) | 0 | 0 | 0 | C | Brothers et al. (2000) |
| *Dasymutilla occidentalis* (Linnaeus, 1758) | Sphecidae | Sphecinae | *Sphex dorsalis* Lepeletier, 1845 | 0 | 0 | 0 | C | Brothers et al. (2000) |
| *Dasymutilla occidentalis* (Linnaeus, 1758) | Sphecidae | Sphecinae | *Sphex ichneumoneus* (Linnaeus, 1758) | 0 | 0 | 0 | P | Manley (1986) |
| *Dasymutilla quadriguttata* (Say, 1823) | Crabronidae | Bembicinae | *Glenostictia pictifrons* (Smith, 1856) | 0 | 0 | 0 | P | Alexander et al. (1993) |
| *Dasymutilla quadriguttata* (Say, 1823) | Scolidae | Scoliinae | *Scolia quinquecincta* Fabricius, 1793 | 0 | 0 | 0 | C | Hayes (1924), Thompson (1972) |
| *Dasymutilla quadriguttata* (Say, 1823) | Vespidae | Polistinae | *Polistes fuscatus* (Fabricius, 1793) | 0 | 1 | 1 | P | Fattig (1943) |
| *Dasymutilla sackenii* (Cresson, 1865) | Crabronidae | Bembicinae | *Bembix occidentalis* Fox, 1893 | 0 | 0 | 0 | C | Bohart & MacSwain (1939) |
| *Dasymutilla scaevola* (Blake 1871) | Crabronidae | Philanthinae | *Cerceris finitima* Cresson, 1865 | 0 | 0 | 0 | P | Shappirio (1949) |
| *Dasymutilla scaevola* (Blake 1871) | Crabronidae | Philanthinae | *Cerceris fumipennis* Say, 1837 | 0 | 0 | 0 | C | Hook (1991) |
| *Dasymutilla snoworum* (Cockerell 1897) | Crabronidae | Philanthinae | *Cerceris fumipennis* Say, 1837 | 0 | 0 | 0 | P | Evans & Rubink (1978) |
| *Dasymutilla vesta* (Cresson 1865) | Crabronidae | Philanthinae | *Philanthus bicinctus* (Mickel, 1916) | 0 | 0 | 0 | P | Evans & O'Neill (1988) |
| *Dasymutilla vesta* (Cresson 1865) | Crabronidae | Philanthinae | *Philanthus bilunatus* Cresson, 1865 | 0 | 0 | 0 | P | Evans & O'Neill (1988) |
| *Dasymutilla vesta* (Cresson 1865) | Crabronidae | Philanthinae | *Philanthus pulcher* Dalla Torre, 1897 | 0 | 0 | 0 | P | Evans & O'Neill (1988) |
| *Dasymutilla vesta* (Cresson 1865) | Halictidae | Halictinae | *Halcitus* sp. | 1 | 0 | 1 | P | Melander & Brues (1903) |
| *Dasymutilla vesta* (Cresson 1865) | Sphecidae | Sceliphrinae | *Chalybion caeruleum* (Linnaeus, 1763) | 0 | 1 | 0 | C | Rucker (1903) |
| *Dasymutilla vestita* (Lepeletier, 1845) | Apidae | Apinae | *Anthophora occidentalis* Cresson, 1869 | 1 | 0 | 0 | C | Mickel (1928) |
| *Dasymutilla vestita* (Lepeletier, 1845) | Apidae | Apinae | *Diadasia rinconis* Cockerell, 1897 | 1 | 0 | 0 | C | Schmidt & Buchmann (1986) Wilson et al. (2010) |
| *Ephutomorpha auricrucis* Rayment, 1953 | Colletidae | Euryglossinae | *Xanthesma maculata* (Smith, 1879) | 1 | 0 | 0 | C | Rayment (1953) |
| *Ephutomorpha emeraldiana* Rayment, 1937 | Halictidae | Halictinae | *Lasioglossum victoriellum* (Cockerell, 1914) | 1 | 0 | 0 | C | Rayment (1937) |
| *Ephutomorpha gondennda* (Rayment, 1955) | Halictidae | Halictinae | *Lasioglossum dimorphum* (Rayment, 1954) | 1 | 0 | 1 | C | Rayment (1955b) |
| *Ephutomorpha mackayensis* (André, 1901) | Crabronidae | Crabroninae | *Sericophorus relucens* Smith, 1856 | 0 | 0 | 0 | P | Matthews & Evans (1970) |
| *Ephutomorpha mackayensis* (André, 1901) | Formicidae | Myrmicinae | *Meranoplus hirsutus* Mayr, 1876 | 0 | 0 | 1 | P | André (1903) |
| *Ephutomorpha melanaderia* (Rayment, 1937) | Halictidae | Halictinae | *Lasioglossum victoriellum* (Cockerell, 1914) | 1 | 0 | 1 | P | Rayment (1937) |
| *Ephutomorpha morosa* (Westwood, 1843) | Stenotritidae | Stenotritinae | *Stenotritus greavesi* (Rayment, 1930) | 1 | 0 | 0 | C | Houston (1984) |
| *Ephutomorpha peremeraldiana* Rayment, 1937 | Halictidae | Halictinae | *Lasioglossum victoriellum* (Cockerell, 1914) | 1 | 0 | 1 | P | Rayment (1937) |
| *Ephutomorpha pulchella* (Smith, 1855) | Formicidae | Dolichoderinae | *Iridomyrmex purpureus* (Smith, 1858) | 0 | 0 | 1 | P | Brothers et al. (2000) |
| *Ephutomorpha redanamelia* Rayment, 1937 | Halictidae | Halictinae | *Lasioglossum victoriellum* (Cockerell, 1914) | 1 | 0 | 1 | P | Rayment (1937) |
| *Ephutomorpha sagittifera* Rayment, 1956 | Halictidae | Nomiinae | *Lipotriches australica* (Smith, 1875) | 1 | 0 | 0 | P | Rayment (1956) |
| *Ephutomorpha* sp. | Crabronidae | Crabroninae | *Sericophorus viridis* (Saussure, 1855) | 0 | 0 | 0 | P | Rayment (1955a) |
| *Ephutomorpha* sp. | Halictidae | Halictinae | *Lasioglossum platycephalum (*Rayment, 1927) | 1 | 0 | 0 | C | Knerer & Schwarz (1978) |
| *Ephutomorpha* sp. near *ferruginata* (Westwood, 1843) | Crabronidae | Bembicinae | *Bembix moma* Evans & Matthews, 1973 | 0 | 0 | 0 | P | Evans & Matthews (1973) |
| *Ephutomorpha* sp. near *peremeraldiana* Rayment, 1937 | Halictidae | Halictinae | *Lasioglossum cyclurum* (Cockerell, 1915) | 1 | 0 | 1 | P | Rayment (1953) |
| *Ephutomorpha tyla* Hearn, Williams & Parslow, 2019 | Colletidae | Hylaeinae | *Amphylaeus morosus* (Smith, 1879) | 1 | 1 | 1 | C | Hearn et al. (2019) |
| *Lomachaeta hicksi* Mickel, 1936 | Crabronidae | Crabroninae | *Solierella blaisdelli* Bridwell, 1920 | 0 | 1 | 0 | C | Parker (1962) Parker & Bohart (1966) |
| *Lomachaeta hicksi* Mickel, 1936 | Crabronidae | Crabroninae | *Solierella plenoculoides* (Fox, 1893) | 0 | 1 | 0 | C | Parker (1962) Parker & Bohart (1966) |
| *Lomachaeta powelli* Mickel, 1964 | Crabronidae | Pemphredoninae | *Diodontus occidentalis* Fox, 1892 | 0 | 0 | 0 | C | Powell (1963) |
| *Ponerotilla clarki* Brothers, 1994 | Formicidae | Ponerinae | *Brachyponera lutea* (Mayr, 1862) | 0 | 0 | 1 | C | Brothers (1994) |
| *Ponerotilla incarinata* Brothers, 1994 | Formicidae | Ponerinae | *Brachyponera lutea* (Mayr, 1862) | 0 | 0 | 1 | C | Brothers (1994) |
| *Ponerotilla lamelligera* Brothers, 1994 | Formicidae | Ponerinae | *Brachyponera lutea* (Mayr, 1862) | 0 | 0 | 1 | P | Brothers (1994) |
| *Ponerotilla lissantyx* Brothers, 1994 | Formicidae | Ponerinae | *Brachyponera lutea* (Mayr, 1862) | 0 | 0 | 1 | C | Brothers (1994) |
| *Reedomutilla heraldica* (Smith, 1855) | Apidae | Apinae | *Melissoptila dama* (Vachal, 1904) | 1 | 0 | 0 | C | Jörgensen (1912b) |
| *Traumatomutilla ocellaris* (Klug, 1821) | Crabronidae | Bembicinae | *Bicyrtes* sp. | 0 | 0 | 0 | C | Bartholomay et al. (2019) |
| *Traumatomutilla* sp. | Apidae | Apinae | *Diadasina distincta* (Holmberg, 1903) | 1 | 0 | 0 | C | Jörgensen (1912a) |
| *Traumatomutilla* sp. | Apidae | Apinae | *Monoeca xanthopyga* Harter-Marques, Cunha and Moure, 2001 | 1 | 0 | 0 | C | da Cunha (2004) |
| *Traumatomutilla sphegea* (Fabricius, 1804) | Crabronidae | Bembicinae | *Stictia signata* (Linnaeus, 1758) | 0 | 0 | 0 | P | Callan (1991), Callan (1990) |
| **Sphaeropthalminae: Pseudomethocini** | | | | | | | | |
| *Anomophotopsis quinteroi* Cambra, 2006 | Halictidae | Halictinae | *Paroxystoglossa transversa* Moure, 1943 | 1 | 0 | 0 | C | Rocha-Filho & Melo (2011) |
| *Atillum albicomum* Mickel, 1943 | Colletidae | Colletinae | *Caupolicana* sp. | 1 | 0 | 0 | P | Fritz (1998) |
| *Atillum charoneum* Mickel, 1943 | Colletidae | Colletinae | *Caupolicana* sp. | 1 | 0 | 0 | P | Fritz (1998) |
| *Atillum venatrix* Mickel, 1943 | Colletidae | Colletinae | *Caupolicana* sp. | 1 | 0 | 0 | P | Fritz (1998) |
| *Calomutilla temporalis* (Gerstaecker, 1874) | Halictidae | Halictinae | *Pseudaugochlora graminea* (Fabricius, 1804) | 1 | 0 | 0 | C | Ihering (1904) |
| *Darditilla araxa* (Cresson, 1902) | Crabronidae | Bembicinae | *Bicyrtes variegatus* (Oliver, 1789) | 0 | 0 | 0 | P | Lopez et al. (2018) |
| *Dimorphomutilla formosa* Mickel, 1938 | Halictidae | Halictinae | *Corynura chloris* (Spinola 1851) | 1 | 0 | 0 | P | Quintero & Cambra (2001) González-Vaquero et al. (2017) |
| *Dimorphomutilla suavissima* (Gerstaecker, 1874) | Halictidae | Halictinae | *Corynura moscosensis* González-Vaquero, 2017 | 1 | 0 | 0 | P | González-Vaquero et al. (2017) |
| *Euspinolia albicoma* Mickel, 1938 | Apidae | Apinae | *Anthophora incerta* Spinola, 1851 | 1 | 0 | 0 | C | Mickel (1938) |
| *Euspinolia rufula* Mickel, 1938 | Apidae | Apinae | *Melitoma* sp. | 1 | 0 | 0 | C | Luz et al. (2016) |
| *Hoplocrates specularis* (Gerstaecker, 1874) | Apidae | Apinae | *Monoeca haemorrhoidalis* (Smith, 1854) | 1 | 0 | 0 | P | Rocha-Filho & Melo (2011) |
| *Hoplognathoca costarricensis* Suárez, 1962 | Apidae | Apinae | *Thygater* sp. | 1 | 0 | 0 | P | Roubik (1989) |
| *Hoplomutilla biplagiata* Mickel, 1939 | Apidae | Apinae | *Centris burgdorfi* Friese, 1900 | 1 | 0 | 0 | C | Luz et al. (2016) |
| *Hoplomutilla conspecta* Mickel, 1939 | Apidae | Apinae | *Eulaema meriana* (Olivier, 1789) | 1 | 1 | 0 | P | Cameron & Ramírez (2001) |
| *Hoplomutilla myops* (Burmeister, 1854) | Apidae | Apinae | *Epicharis bicolor* Smith, 1854 | 1 | 0 | 0 | C | Rocha-Filho et al. (2008) |
| *Hoplomutilla opima* Mickel, 1939 | Apidae | Apinae | *Centris rufosuffusa* Cockerell, 1935 | 1 | 0 | 0 | P | Callan (1977) |
| *Hoplomutilla spinosa* (Swederus, 1787) | Apidae | Apinae | *Eulaema nigrita* Lepeletier, 1841 | 1 | 1 | 0 | C | Quintero & Cambra (2001) |
| *Hoplomutilla triumphans* Mickel, 1939 | Apidae | Apinae | *Eufriesea* sp. | 1 | 0 | 0 | C | Lenko (1964) |
| *Hoplomutilla xanthocerata* (Smith, 1862) | Apidae | Apinae | *Eulaema meriana* (Olivier, 1789) | 1 | 1 | 0 | C | Roubik (1990) |
| *Horcomutilla projectifrons* (Cresson, 1902) | Apidae | Apinae | *Exomalopsis auropilosa* Spinola, 1853 | 1 | 0 | 0 | C | Lopez et al. (2019) |
| *Lophomutilla corupa* Casal, 1968 | Halictidae | Halictinae | *Lasioglossum seabrai* (Moure, 1956) | 1 | 0 | 1 | C | Bergamaschi et al. (2010) |
| *Lophomutilla halicta* (Mickel 1973) | Halictidae | Halictinae | *Augochlorella edentata* Michener, 1954 | 1 | 0 | 1 | P | Clarence & Mickel (1973) |
| *Lophomutilla inca* Fritz and Pagliano, 1993 | Halictidae | Halictinae | *Neocorynura* sp. | 1 | 0 | 0 | P | Luz et al. (2016) |
| *Lophomutilla mickeli* Suárez 1962 | Apidae | Apinae | *Monoeca haemorrhoidalis* (Smith, 1854) | 1 | 0 | 0 | P | Rocha-Filho & Melo (2011) |
| *Lophostigma cincta* (du Buysson, 1892) | Halictidae | Halictinae | *Megalopta ecuadoria* Friese, 1926 | 1 | 1 | 1 | C | Cambra et al. (2005) |
| *Lophostigma cincta* (du Buysson, 1892) | Halictidae | Halictinae | *Megalopta genalis* Meade-Waldo, 1916 | 1 | 1 | 1 | C | Cambra et al. (2005) |
| *Lynchiatilla parana* Cambra, 2012 | Halictidae | Halictinae | *Paroxystoglossa spiloptera*  Moure, 1960 | 1 | 0 | 0 | C | Bergamaschi et al. (2012) |
| *Myrmilloides grandiceps* (Blake, 1872) | Halictidae | Halictinae | *Augochlorella persimilis* (Viereck, 1910) | 1 | 0 | 1 | P | Ordway (1964) |
| *Myrmilloides grandiceps* (Blake, 1872) | Halictidae | Halictinae | *Augochlorella striata* (Provancher, 1888) | 1 | 0 | 1 | P | Ordway (1964) |
| *Pappognatha limes* Mickel, 1939 | Apidae | Apinae | *Euglossa ignita* Smith, 1874 | 1 | 1 | 0 | C | Luz et al. (2016) |
| *Pappognatha myrmiciformis* (Cameron, 1897) | Apidae | Apinae | *Euglossa dodsoni* Moure, 1965 | 1 | 1 | 0 | C | Quintero & Cambra (2005) |
| *Pappognatha myrmiciformis* (Cameron, 1897) | Formicidae | Formicinae | *Camponotus sericeiventris* Guérin, 1838 | 0 | 1 | 1 | P | Wheeler (1983) |
| *Pappognatha panamensis* Quintero & Cambra, 2005 | Apidae | Apinae | *Euglossa hemichlora* Cockerell, 1917 | 1 | 1 | 0 | C | Cambra et al. (2015) |
| *Pappognatha panamensis* Quintero & Cambra, 2005 | Apidae | Apinae | *Euglossa imperialis* Cockerell, 1922 | 1 | 1 | 0 | C | Cambra et al. (2015) |
| *Pappognatha patruelis* (Andre, 1898) | Apidae | Apinae | *Euglossa* sp. | 1 | 1 | 0 | C | Luz et al. (2016) |
| *Pappognatha speciosa* Mickel, 1939 | Apidae | Apinae | *Euglossa intersecta* Latreille, 1838 | 1 | 1 | 0 | P | Quintero & Cambra (2005) |
| *Pseudomethoca bethae* Krombein, 1991 | Apidae | Apinae | *Exomalopsis solani* Cockerell, 1896 | 1 | 0 | 0 | C | Krombein(1992) |
| *Pseudomethoca frigida* (Smith, 1855) | Halictidae | Halictinae | *Augochlorella striata* (Provancher, 1888) | 1 | 0 | 1 | P | Michener & Wille (1961) |
| *Pseudomethoca frigida* (Smith, 1855) | Halictidae | Halictinae | *Lasioglossum cinctipes* (Provancher, 1888) | 1 | 0 | 1 | C | Knerer & Atwood (1966) |
| *Pseudomethoca frigida* (Smith, 1855) | Halictidae | Halictinae | *Lasioglossum coeruleum* (Robertson 1893) | 1 | 1 | 1 | C | Brothers (1972) |
| *Pseudomethoca frigida* (Smith, 1855) | Halictidae | Halictinae | *Lasioglossum imitatum* (Smith, 1853) | 1 | 0 | 1 | P | Brothers (1972) |
| *Pseudomethoca frigida* (Smith, 1855) | Halictidae | Halictinae | *Lasioglossum laevissimum* (Smith, 1853) | 1 | 0 | 1 | P | Brothers (1972) |
| *Pseudomethoca frigida* (Smith, 1855) | Halictidae | Halictinae | *Lasioglossum versatum* (Robertson, 1902) | 1 | 0 | 1 | C | Michener (1966) |
| *Pseudomethoca frigida* (Smith, 1855) | Halictidae | Halictinae | *Lasioglossum zephyrum* (Smith, 1853) | 1 | 0 | 1 | C | Brothers (1972) |
| *Pseudomethoca gila* (Blake, 1871) | Andrenidae | Panurginae | *Pseudopanurgus rugosus* (Robertson, 1895) | 1 | 0 | 0 | P | Mnaley & Neff (1989) |
| *Pseudomethoca hesperus* Brothers, 1982 | Halictidae | Halictinae | *Halictus hesperus* Smith, 1862 | 1 | 1 | 1 | P | Brothers (1982) |
| *Pseudomethoca hoplitiformis* (Strand, 1909) | Apidae | Apinae | *Ptilothrix plumata* Smith, 1853 | 1 | 0 | 0 | P | Friese (1908) Strand (1909) |
| *Pseudomethoca mulaiki* Mickel, 1938 | Crabronidae | Philanthinae | *Trachypus mexicanus* Saussure, 1867 | 0 | 0 | 0 | P | Krombein(1992) |
| *Pseudomethoca perditrix* Krombein, 1991 | Andrenidae | Panurginae | *Perdita portalis* Timberlake, 1954 | 1 | 0 | 0 | C | Krombein(1992) |
| *Pseudomethoca propinqua* (Cresson, 1865) | Andrenidae | Panurginae | *Triepolus wyomingensis* Cockerell, 1905 | 1 | 0 | 0 | P | Krombein(1992) |
| *Pseudomethoca propinqua* (Cresson, 1865) | Apidae | Apinae | *Diadasia rinconis* Cockerell, 1897 | 1 | 0 | 0 | P | Parks (2018) |
| *Pseudomethoca propinqua* (Cresson, 1865) | Apidae | Apinae | *Melissodes pallidisignata* Cockerell, 1905 | 1 | 0 | 0 | P | Krombein(1992) |
| *Pseudomethoca propinqua* (Cresson, 1865) | Halictidae | Halictinae | *Lasioglossum zephyrum* (Smith, 1853) | 1 | 0 | 1 | P | Melander & Brues (1903) |
| *Pseudomethoca propinqua* (Cresson, 1865) | Halictidae | Nomiinae | *Nomia melandri* Cockerell, 1906 | 1 | 0 | 0 | P | Krombein(1992) |
| *Pseudomethoca pumila* Burmeister, 1854 | Halictidae | Halictinae | *Lasioglossum seabrai* (Moure, 1956) | 1 | 0 | 1 | C | Bergamaschi et al. (2011) |
| *Pseudomethoca sanbornii* (Blake, 1871) | Crabronidae | Philanthinae | *Cerceris rufopicta* Smith, 1856 | 0 | 0 | 0 | P | Alexander & Asis (1997) |
| *Pseudomethoca sanbornii* (Blake, 1871) | Halictidae | Nomiinae | *Nomia pattoni* Cockerell, 1910 | 1 | 0 | 0 | C | Mickel (1924a) |
| *Pseudomethoca simillina* (Smith, 1855) | Crabronidae | Philanthinae | *Cerceris rufopicta* Smith, 1856 | 0 | 0 | 0 | P | Alexander & Asis (1997) |
| *Pseudomethoca* sp. | Halictidae | Halictinae | *Augochloropsis iris* (Schrottky, 1902) | 1 | 0 | 1 | C | Coelho (2002) |
| *Pseudomethoca spixi* (Diller, 1989) | Apidae | Apinae | *Monoeca haemorrhoidalis* (Smith, 1854) | 1 | 0 | 0 | P | Rocha-Filho & Melo (2011) |
| *Pseudomethoca torrida* Krombein, 1954 | Andrenidae | Panurginae | *Perdita graenicheri* Timberlake, 1947 | 1 | 0 | 0 | P | Krombein(1992) |
| *Pseudomethoca toumeyi* (Fox, 1894) | Andrenidae | Panurginae | *Perdita portalis* Timberlake, 1954 | 1 | 0 | 0 | P | Krombein(1992) |
| *Pseudomethoca toumeyi* (Fox, 1894) | Crabronidae | Bembicinae | *Hapalomellinus albitomentosus* (Bradley, 1920) | 0 | 0 | 0 | P | Krombein(1992) |
| *Pseudomethoca willei* Mickel, 1969 | Halictidae | Halictinae | *Lasioglossum umbripenne* (Ellis, 1913) | 1 | 0 | 1 | C | Mickel (1969) |
| **Sphaeropthalminae: Sphaeropthalmini** | | | | | | | | |
| *Cystomutilla ruficeps* (Smith, 1855) | Crabronidae | Crabroninae | *Ectemnius rubicola* (Dufour & Perris, 1840) | 0 | 0 | 0 | C | Bogusch (2006), Dufour & Perris (1840), Borries (1892) |
| *Cystomutilla ruficeps* (Smith, 1855) | Crabronidae | Pemphredoninae | *Pemphredon rugifer* (Dahlbom, 1844) | 0 | 1 | 0 | C | Lelej (1985) |
| *Cystomutilla ruficeps* (Smith, 1855) | Crabronidae | Pemphredoninae | *Pemphredon wesmaeli* (Morawitz, 1864) | 0 | 1 | 0 | P | Bogusch (2006), Ferton (1908), André (1899–1903) |
| *Dilophotopsis concolor* (Cresson, 1865) | Crabronidae | Crabroninae | *Tachysphex* sp. | 0 | 0 | 0 | C | Pitts (2003) |
| *Hoplocrates specularis* (Gerstaecker, 1874) | Apidae | Apinae | *Monoeca catarina* Aguiar, 2012 | 1 | 0 | 0 | P | Zillikens & Steiner (2020) |
| *Morsyma ashmeadii* Fox, 1899 | Crabronidae | Pemphredoninae | *Diodontus occidentalis* Fox, 1892 | 0 | 0 | 0 | C | Powell (1963) |
| *Odontophotopsis succinea* Viereck, 1903 | Crabronidae | Crabroninae | *Oxybelus uniglumis* (Linnaeus, 1758) | 0 | 0 | 0 | C | Pitts & Parker (2003) |
| *Sphaeropthalma abdominalis* (Blake, 1886) | Crabronidae | Crabroninae | *Trypoxylon tridentatum* Packard, 1867 | 0 | 1 | 0 | C | Pitts et al. (2004) |
| *Sphaeropthalma abdominalis* (Blake, 1886) | Megachilidae | Megachilinae | *Ashmeadiella aridula* Cockerell, 1910 | 1 | 1 | 0 | C | Pitts et al. (2004) |
| *Sphaeropthalma abdominalis* (Blake, 1886) | Megachilidae | Megachilinae | *Ashmeadiella gillettei* Titus, 1904 | 1 | 1 | 0 | C | Pitts et al. (2004) |
| *Sphaeropthalma abdominalis* (Blake, 1886) | Megachilidae | Megachilinae | *Ashmeadiella meliloti* (Cockerell, 1897) | 1 | 1 | 0 | C | Pitts et al. (2004) |
| *Sphaeropthalma abdominalis* (Blake, 1886) | Megachilidae | Megachilinae | *Ashmeadiella timberlakei* Michener, 1936 | 1 | 1 | 0 | C | Pitts et al. (2004) |
| *Sphaeropthalma abdominalis* (Blake, 1886) | Megachilidae | Megachilinae | *Hoplitis hypocrita* (Cockerell, 1906) | 1 | 1 | 0 | C | Pitts et al. (2004) |
| *Sphaeropthalma abdominalis* (Blake, 1886) | Megachilidae | Megachilinae | *Hoplitis producta* (Cresson, 1864) | 1 | 1 | 0 | C | Pitts et al. (2004) |
| *Sphaeropthalma abdominalis* (Blake, 1886) | Megachilidae | Megachilinae | *Megachile montivaga* Cresson, 1878 | 1 | 1 | 0 | C | Pitts et al. (2004) |
| *Sphaeropthalma abdominalis* (Blake, 1886) | Megachilidae | Megachilinae | *Stelis lateralis* Cresson, 1864 | 0 | 1 | 0 | P | Pitts et al. (2004) |
| *Sphaeropthalma abdominalis* (Blake, 1886) | Sapygidae | Sapyginae | *Sapyga pumila* Cresson, 1880 | 0 | 1 | 0 | C | Pitts et al. (2004) |
| *Sphaeropthalma abdominalis* (Blake, 1886) | Vespidae | Eumeninae | *Microdynerus bakerianus* (Cameron, 1908) | 0 | 1 | 0 | C | Pitts et al. (2004) |
| *Sphaeropthalma amphion* (Fox, 1899) | Crabronidae | Crabroninae | *Pisonopsis birkmanni* Rohwer, 1909 | 0 | 1 | 0 | C | Pitts et al. (2004) |
| *Sphaeropthalma amphion* (Fox, 1899) | Crabronidae | Crabroninae | *Trypoxylon tridentatum* Packard, 1867 | 0 | 1 | 0 | P | Pitts et al. (2004) |
| *Sphaeropthalma amphion* (Fox, 1899) | Megachilidae | Megachilinae | *Ashmeadiella bigeloviae* (Cockerell, 1897) | 1 | 1 | 0 | C | Pitts et al. (2004) |
| *Sphaeropthalma amphion* (Fox, 1899) | Megachilidae | Megachilinae | *Ashmeadiella gillettei* Titus, 1904 | 1 | 1 | 0 | C | Pitts et al. (2004) |
| *Sphaeropthalma amphion* (Fox, 1899) | Megachilidae | Megachilinae | *Ashmeadiella meliloti* (Cockerell, 1897) | 1 | 1 | 0 | P | Pitts et al. (2004) |
| *Sphaeropthalma amphion* (Fox, 1899) | Megachilidae | Megachilinae | *Atoposmia copelandica* (Cockerell, 1908) | 1 | 1 | 0 | C | Pitts et al. (2004) |
| *Sphaeropthalma amphion* (Fox, 1899) | Megachilidae | Megachilinae | *Atoposmia hypostomalis* (Michener, 1949) | 1 | 1 | 0 | C | Pitts et al. (2004) |
| *Sphaeropthalma amphion* (Fox, 1899) | Megachilidae | Megachilinae | *Hoplitis bullifacies* Michener, 1947 | 1 | 1 | 0 | C | Pitts et al. (2004) |
| *Sphaeropthalma amphion* (Fox, 1899) | Megachilidae | Megachilinae | *Hoplitis fulgida* (Cresson, 1864) | 1 | 1 | 0 | P | Pitts et al. (2004) |
| *Sphaeropthalma amphion* (Fox, 1899) | Megachilidae | Megachilinae | *Hoplitis grinnelli* Cockerell, 1910 | 1 | 1 | 0 | P | Pitts et al. (2004) |
| *Sphaeropthalma amphion* (Fox, 1899) | Megachilidae | Megachilinae | *Hoplitis sambuci* Titus, 1904 | 1 | 1 | 0 | C | Pitts et al. (2004) |
| *Sphaeropthalma amphion* (Fox, 1899) | Megachilidae | Megachilinae | *Osmia marginata* Michener, 1936 | 1 | 0 | 0 | C | Pitts et al. (2004) |
| *Sphaeropthalma amphion* (Fox, 1899) | Sapygidae | Sapyginae | *Sapyga aculeata* Cresson 1865 | 0 | 1 | 0 | C | Pitts et al. (2004) |
| *Sphaeropthalma amphion* (Fox, 1899) | Sapygidae | Sapyginae | *Sapyga elegans* Cresson, 1880 | 0 | 1 | 0 | C | Pitts et al. (2004) |
| *Sphaeropthalma amphion* (Fox, 1899) | Vespidae | Eumeninae | *Ancistrocerus catskill* (de Saussure, 1853) | 0 | 1 | 0 | P | Pitts et al. (2004) |
| *Sphaeropthalma amphion* (Fox, 1899) | Vespidae | Eumeninae | *Ancistrocerus simulator* Cameron, 1908 | 0 | 1 | 0 | C | Pitts et al. (2004) |
| *Sphaeropthalma amphion* (Fox, 1899) | Vespidae | Eumeninae | *Leptochilus chiricahua* Parker, 1966 | 0 | 1 | 0 | P | Pitts et al. (2004) |
| *Sphaeropthalma amphion* (Fox, 1899) | Vespidae | Eumeninae | *Leptochilus rufinodus* (Cresson, 1868) | 0 | 1 | 0 | C | Pitts et al. (2004) |
| *Sphaeropthalma blakeii* (Fox, 1893) | Apidae | Apinae | *Diadasia vallicola* Timberlake, 1940 | 1 | 0 | 0 | C | Ferguson (1962) |
| *Sphaeropthalma ferruginea* (Blake, 1879) | Sphecidae | Sphecinae | *Isodontia elegans* (Smith, 1856) | 0 | 1 | 0 | C | Ferguson (1962) |
| *Sphaeropthalma gayi* (Mickel, 1937) | Megachilidae | Fideliinae | *Neofidelia profuga* Moure & Michener, 1955 | 1 | 0 | 0 | C | Rozen (1973) |
| *Sphaeropthalma orestes* (Fox, 1899) | Crabronidae | Crabroninae | *Tachysphex pompiliformis* (Panzer,1805) | 0 | 0 | 0 | C | Ferguson (1962) |
| *Sphaeropthalma orestes* (Fox, 1899) | Megachilidae | Megachilinae | *Anthidium collectum collectum* Huard, 1896 | 1 | 0 | 0 | C | Ferguson (1962) |
| *Sphaeropthalma orestes* (Fox, 1899) | Vespidae | Eumeninae | *Euodynerus auranus* (Cameron, 1906) | 0 | 0 | 0 | P | Ferguson (1962) |
| *Sphaeropthalma pennsylvanica* (Lepeletier, 1845) | Crabronidae | Crabroninae | *Trypoxylon clavatum* Say, 1837 | 0 | 1 | 0 | C | Pitts et al. (2010) |
| *Sphaeropthalma pennsylvanica* (Lepeletier, 1845) | Crabronidae | Crabroninae | *Trypoxylon collinum* Smith, 1856 | 0 | 1 | 0 | C | Pitts et al. (2010) |
| *Sphaeropthalma pennsylvanica* (Lepeletier, 1845) | Crabronidae | Crabroninae | *Trypoxylon lactitarse* Saussure 1842 | 0 | 1 | 0 | C | Pitts et al. (2010) |
| *Sphaeropthalma pennsylvanica* (Lepeletier, 1845) | Crabronidae | Crabroninae | *Trypoxylon politum* Say, 1837 | 0 | 1 | 0 | C | Rau (1928) Pitts et al. (2010) |
| *Sphaeropthalma pennsylvanica* (Lepeletier, 1845) | Crabronidae | Crabroninae | *Trypoxylon striatum* Provancher, 1888 | 0 | 1 | 0 | C | Krombein(1967) |
| *Sphaeropthalma pennsylvanica* (Lepeletier, 1845) | Crabronidae | Crabroninae | *Trypoxylon tridentatum* Packard, 1867 | 0 | 1 | 0 | C | Pitts et al. (2010) Krombein (1967) |
| *Sphaeropthalma pennsylvanica* (Lepeletier, 1845) | Pompilidae | Pepsinae | *Auplopus architectus* (Say, 1836) | 0 | 1 | 0 | C | Krombein(1967) |
| *Sphaeropthalma pennsylvanica* (Lepeletier, 1845) | Pompilidae | Pepsinae | *Auplopus mellipes* (Say, 1836) | 0 | 1 | 0 | C | Krombein (1967) |
| *Sphaeropthalma pennsylvanica* (Lepeletier, 1845) | Sphecidae | Sceliphrinae | *Sceliphron caementarium* (Drury, 1773) | 0 | 1 | 0 | C | Rau & Rau (1916), Pitts et al. (2010) |
| *Sphaeropthalma pennsylvanica* (Lepeletier, 1845) | Sphecidae | Sphecinae | *Isodontia mexicana* (Saussure, 1867) | 0 | 1 | 0 | C | Manley & Carithers (1998) Pitts at al. (2010) |
| *Sphaeropthalma pennsylvanica* (Lepeletier, 1845) | Vespidae | Eumeninae | *Symmorphus albomarginatus* (de Saussure, 1855) | 0 | 1 | 0 | C | Pitts et al. (2010) |
| *Sphaeropthalma sp.* | Vespidae | Masarinae | *Pseudomasaris edwardsii* (Cresson, 1872) | 1 | 0 | 0 | P | Hicks (1929) |
| *Sphaeropthalma unicolor* (Cresson, 1865) | Apidae | Apinae | *Anthophora bomboides* Kirby, 1837 | 1 | 0 | 0 | C | Ferguson (1962) |
| *Sphaeropthalma unicolor* (Cresson, 1865) | Apidae | Apinae | *Anthophora linsleyi* Timberlake, 1941 | 1 | 0 | 0 | C | Ferguson (1962) |
| *Sphaeropthalma unicolor* (Cresson, 1865) | Apidae | Apinae | *Anthophora occidentalis* Cresson, 1869 | 1 | 0 | 0 | C | Ferguson (1962) |
| *Sphaeropthalma unicolor* (Cresson, 1865) | Apidae | Apinae | *Anthophora urbana urbana* Cresson 1878 | 1 | 0 | 0 | C | Torchio & Trostle (1986) |
| *Sphaeropthalma unicolor* (Cresson, 1865) | Apidae | Apinae | *Diadasia bitubercolata* (Cresson, 1878) | 1 | 0 | 0 | C | Ferguson (1962) |
| *Sphaeropthalma unicolor* (Cresson, 1865) | Apidae | Apinae | *Melissodes robustior* Cockerell, 1915 | 1 | 0 | 0 | C | Ferguson (1962) |
| *Sphaeropthalma unicolor* (Cresson, 1865) | Megachilidae | Megachilinae | *Anthidium illustre* Cresson, 1879 | 1 | 1 | 0 | C | Hurd (1979) |
| *Sphaeropthalma unicolor* (Cresson, 1865) | Megachilidae | Megachilinae | *Ashmeadiella californica* (Ashmead, 1897) | 1 | 1 | 0 | C | Ferguson (1962) |
| *Sphaeropthalma unicolor* (Cresson, 1865) | Megachilidae | Megachilinae | *Notanthidium* sp. | 1 | 0 | 0 | C | Ferguson (1962) |
| *Sphaeropthalma unicolor* (Cresson, 1865) | Sphecidae | Sphecinae | *Isodontia elegans* (Smith, 1856) | 0 | 1 | 0 | C | Davidson (1899) |
| *Sphaeropthalma uro* (Blake, 1879) | Crabronidae | Crabroninae | *Trypoxylon tridentatum* Packard, 1867 | 0 | 1 | 0 | C | Pitts et al. (2004) Krombein (1967) |
| *Sphaeropthalma uro* (Blake, 1879) | Megachilidae | Megachilinae | *Ashmeadiella gillettei* Titus, 1904 | 1 | 1 | 0 | C | Pitts et al. (2004) |
| *Sphaeropthalma uro* (Blake, 1879) | Megachilidae | Megachilinae | *Dianthidium curvatum* (Smith, 1854) | 1 | 0 | 0 | C | Pitts et al. (2004) Fischer (1951) |
| *Sphaeropthalma uro* (Blake, 1879) | Megachilidae | Megachilinae | *Hoplitis biscutellae* (Cockerell, 1897) | 1 | 1 | 0 | C | Pitts et al. (2004) |
| *Sphaeropthalma uro* (Blake, 1879) | Sapygidae | Sapyginae | *Sapyga pumila* Cresson, 1880 | 0 | 1 | 0 | C | Pitts et al. (2004) |
| *Sphaeropthalma uro* (Blake, 1879) | Vespidae | Eumeninae | *Pachodynerus astraeus* Cameron, 1905 | 0 | 0 | 0 | P | Pitts et al. (2004) |
| *Tallium aracati* Casal, 1962 | Apidae | Apinae | *Centris burgdorfi* Friese, 1900 | 1 | 0 | 0 | C | Luz et al. (2016) |
| *Xystromutilla asperiventris* André, 1905 | Crabronidae | Crabroninae | *Trypoxylon* sp. | 0 | 1 | 0 | C | Morato (1994) |
| *Xystromutilla asperiventris* André, 1905 | Sphecidae | Sceliphrinae | *Podium* sp. | 0 | 1 | 0 | C | Morato (1994) |
| *Xystromutilla turrialba* Casal, 1969 | Crabronidae | Crabroninae | *Trypoxylon* sp. | 0 | 1 | 0 | C | Cambra & Quintero (2004) |
| *Xystromutilla turrialba* Casal, 1969 | Sphecidae | Sceliphrinae | *Podium* sp. | 0 | 1 | 0 | C | Cambra & Quintero (2004) |

**References**

Abe S. The nest of Toramaruhanabachi (“tiger wasp”) (in Japanese). Konchû 1932;5: 245–247.

Alexander BA, Asis JD. Patterns of Nest Occupancy and Provisioning in Cerceris rufopicta Smith (Hymenoptera: Sphecidae). J Insect Behav. 1997;10: 6.

Alexander BA, Minckley RL, Yanega D. Nesting Biology of Glenostictia pictifrons (F. Smith) (Hymenoptera: Sphecidae: Bembicini). J Kansas Entomol Soc. 1993;66(1): 108-120.

Amiet F. Vespoidea 1 Mutillidae Sapygidae Scoliidae Tiphiidae. Fauna Helvetica 23. Switzerland: Centre suisse de cartographie de la faune und Schweizerische Entomologische Gesellschaft; 2008.

André E. Species des Hyménoptères d’Europe & d’Algérie. Tome Huitième. Les Mutillides. Paris: Dubosclard VVE, Hermann A; 1899-1903. pp. 1–64 + pls. 1–8 (1899), pp. 65–144 (1900), pp. 145–304 (1901), pp. 305–384 (1902), 385–478 (1903), pls. 9–15 (year unknown).

André E. Quatrième contribution a la connaisance des Mutillides de l’Australie. Mém Soc Zool France. 1903;15: 240–278.

André E. Nouvelles espèces de Mutillides d’Amérique (Hym.). Z Hymen Dipt. 1906;6: 33–48, 65–80, 161–169.

Aranda R, Graciolli G. First report of Exomalopsis fulvofasciata (Hymenoptera: Anthophoridae) as host of two Timulla species (Hymenoptera: Mutillidae). Biota Neotrop. 2013;13(4): 382-384.

Asìs JD, Tormos J, Gayubo SF. Nesting behaviour and provisioning in Bembix merceti and Bembix zonata (Hymenoptera: Crabronidae). J Nat Hist. 2004;38: 1799–1809.

Asís, JD, Gayubo SF, Tormos J. Notes on the natural history of Stizus perrisii ibericus Beaumont (Hymenoptera: Sphecidae). J Nat Hist. 1991;25: 5,1331-1337.

Bartholomay R, Williams KA, Lopez V, de Oliveira ML. Revision of the Traumatomutilla americana species group (Hymenoptera: Mutillidae) Zootaxa. 2019;4608(1): 001-034.

Bayliss PS, Brothers DJ. Behaviour and Host Relationships of Dolichomutilla sycorax (Smith) (Hymenoptera: Mutillidae, Sphecidae). J Hymenopt Res. 2001,10: 1-9.

Beljavsky AG. The Mutilla europaea, L. as a bee enemy. The Bee World. 1935;16: 122.

Bergamaschi ACB, Cambra R, Melo GAR. Male description and host record for Lophomutilla corupa Casal, 1968 (Hymenoptera: Mutillidae), with behavioural notes on mating behaviour and host nest attacks. J Nat Hist. 2010;44(43): 2597-2607.

Bergamaschi ACB, Cambra R, Melo GAR. New combinations, sex association, behavioural notes and potential host record for two Neotropical species of Pseudomethoca Ashmead, 1896 (Hymenoptera: Mutillidae). Zootaxa. 2011;3062: 55-63.

Bergamaschi ACB, Cambra R, Brothers DJ, Melo GAR. Lynchiatilla Casal, 1963 (Hymenoptera: Mutillidae): a new species from Brazil associated with Paroxystoglossa spiloptera Moure (Hymenoptera: Apidae: Halictinae), and notes on other species. Zootaxa. 2012;3548: 55-64.

Bogusch P. The velvet ants (Hymenoptera: Mutillidae) of the Czech Republic and Slovakia: an identification key and annotated checklist. Acta Mus Moraviae, Sci biol. 2006;91: 103-148.

Bogusch P, Hlaváčková L, Petr L, Bosch J. Nest structure, pollen utilization and parasites associated with two West-Mediterranean bees (Hymenoptera, Apiformes, Megachilidae) nesting in empty snail shells. J Hymenopt Res. 2020;76: 113-125.

Bohart GE, MacSwain JW. The Life History of the Sand Wasp, Bembix occidentalis beutenmuelleri Fox, and its Parasites. Bull S Calif Acad Sci. 1939;38: 84-98.

Borries H. Mutilla erythrocephala Fabr. som parasit hos Crabro (Solenius) rubicola D. & P. Entomol Tidskr. 1892;13: 247-249.

Bouvier EL. The Psychic Life of Insects. London: T. Fisher Unwin Ltd; 1922.

Bradley JC. Untiteled note in minutes of meeting of 22 May 1919 Ent Sect. Acad Nat Sci Philadelphia Ent News. 1920;31: 112-113.

Brothers DJ. A New Genus and Four New Species of Mutillidae Associated with Brachyponera lutea Mayr (Formicidae) in Western Australia. J Aust Ent Soc. 1994;33: 143-152.

Brothers DJ, Tschuch G, Burger F. Associations of mutillid wasps (Hymenoptera, Mutillidae) with eusocial insects. Insectes Soc. 2000;47: 201-211.

Brothers DJ. Gregarious parasitoidism in Australian Mutillidae (Hymenoptera). Aust Entomol Mag. 1984;11: 8-10.

Brothers DJ. Biology and immature stages of Pseudomethoca f. frigida, with notes on other species (Hymenoptera: Mutillidae). Kans Sci Bull. 1972;50: 1-38.

Brothers DJ. Biology and immature stages of Myrmosula parvula (Hymenoptera: Mutillidae). J Kansas Entomol Soc. 1978;51: 698-710.

Brothers DJ. Two new species of Mutillidae associated with Halictus hesperus (Halictidae) in Panama (Hymenoptera). Sociobiology. 1982;7: 205-212.

Bryant AHR. More about the “Cow-killer” (Mutilla coccinea). Am Entomol Bot. 1870;2: 337.

Byers GW. Mutillid parasitoid of the cicada killer? Sphecos. 1991;21: 4-5.

Callan EM. Mutillidae of Trinidad. Sphecos. 1990;19, 21-22.

Callan EM. Nesting behavior and prey of sand wasps in Trinidad (Hymenoptera: Sphecidae: Nyssoninae). The Entomologist. 1991;110(3): 134-138.

Callan EM. A note on Timulla (Timulla) eriphyla Mickel (Hym., Mutillidae), a parasite of Tachysphex blatticidus F.X. Williams (Hym., Larridae), from Trinidad. B.W.I. Proc R Entomol Soc. 1942;17: 18.

Callan EM. Observations on Centris rufosuffusa Cockerell (Hymenoptera: Anthophoridae) and its parasites. J Nat Hist. 1977;11: 127-135.

Cambra RA. Dasymutilla Ashmead (Hymenoptera, Mutillidae) in Panama: new species, sex associations and seasonal flight activity. Insecta Mundi 2018;0608.

Cambra RA, Quintero AD. New species of Xystromutilla André (Hymenoptera: Mutillidae) and the first illustrated key for the males of the genus. T Am Entomol Soc. 2004;130(4): 463-478.

Cambra RA, Buschini MLT, Arias DQ, Brozoski F, Lustoga RP. Ephuta icema Casal, 1969 and its host Auplopus subaurarius Dreisbach, 1963 (Hymenoptera: Mutillidae, Pompilidae) from Brazil. Zootaxa. 2017;4272(2): 285.

Cambra RA, Gonzalez VH, Wcislo WT. Description of the male, host associations, and new distribution records for Lophostigma cincta (du Buysson) (Hymenoptera: Mutillidae). Proc Entomol Soc Wash. 2005;107: 229-234.

Cambra RA, Roubik DW, Quintero DA. Hospederos de Pappognatha panamensis Quintero & Cambra, 2005 (Hymenoptera: Mutillidae) y su primer registro de distribuición para Costa Rica. Bol Mus Entomol Univ Valle. 2015;16: 5-7.

Cameron SA, Ramírez S. Nest Architecture and Nesting Ecology of the Orchid Bee Eulaema meriana (Hymenoptera: Apinae: Euglossini). J Kansas Entomol Soc. 2001; 74(3): 142-165.

Cameron P. Hymenoptera Orientalis (!), or Contributions to a knowledge of the Hymenoptera of the Indian Zoological Region. Part IV. Mem Proc Manch Lit Philos Soc. 1892;35: 97-137 + pl. 1. [Mutillidae – p. 116-137 + pl. 1].

Carpenter G. Note on the bionomics of the sphegid wasp Dasyproctus bipunctatus Lepeletier. Proc R Entomol Soc. 1942;47: 48.

Clarence E, Mickel CE. Paramutilla halicta n. genus, n. species, a parasite of the halictine bee Augochlorella edentata (Hymenoptera: Mutillidae). Kansas Entomol Soc. 1973;46: 1.

Coelho BWT. The biology of the primitively eusocial Augochloropsis iris (Schrottky, 1902) (Hymenoptera, Halictidae). Insectes Soc. 2002;49: 181-190.

Cottrell RG. The biology of Dasymutilla bioculata (Cresson). M.S. thesis. University of Minnesota; 1936.

Crèvecoeur A. Recherches biologiques sur Smicromyrme (Mutilla) rufipes F. Bull Annals Soc Ent Belg. 1930;70: 271.

da Cunha R. Monoeca xanthopyga (Hymenoptera, Apoidea, Tapinotaspidini), primeiro registro de hospedeiro para parasitoide do gênero Traumatomutilla (Hymenoptera: Mutillidae) na Serra Geral do Rio Grande do Sul. Acta Sci. 2004;6(2): 35-40.

Dahlbom AG. (1847) Bekräftelse på den förmoden att Sågflugor i hungersnöd angripa och uppäla hvrarandra; Galläplen förosakade af Sågflugor; Mutillæ Europææ parasitiska lefnadssätt. Forh skand Naturf fjerde Möde. 1847;1844, 3: 272–277.

Davidson A. Sphex elegans. Entomol News. 1899;10: 179-180.

Hearn LR, Williams KA, Stevens MI, Schwarz MP, Davies OK, Parslow BA. Description and novel host records for a new species of Australian mutillid wasp (Hymenoptera: Mutillidae) from hylaeine bee nests (Hymenoptera: Colletidae). Aust Entomol. 2019;58, 3: 524-532.

Lopez V, Guillermo-Ferreira R, Trad B, Silvestre R. Description of the male of Darditilla araxa (Cresson, 1902) (Hymenoptera, Mutillidae) with geographical distribution, biological notes and key to males of Brazil. Zootaxa. 2018;4532(1): 104.

De-Stefani Perez T. (1886) Raccolte imenotterologiche sui monti di Renda e loro adiacenze (Cont. V. N. prec.). Nat Sicil. 1886;5: 169-172.

De-Stefani Perez T. Notizie imenotterologiche, Fam. Diploptera (Cont. V. Num. prec.), Vespidae. Nat Sicil. 1882;2: 55-58.

Deyrup M, Manley D. Sex-Biased Size Variation in Velvet Ants (Hymenoptera: Mutillidae). Fla Entomol. 1986;69(2): 327-335.

Donald GM. An aberrant female and possible new host record for Dasymutilla occidentalis (Hymenoptera: Mutillidae). J Entomol Sci. 1986;21(4): 367-367.

Drewsen C. Mutilla europaea Linné. Stett Ent Zeit. 1847;8: 210-211.

Dufour L, Perris E. Mémoire sur les Insectes Hyménoptères qui nichent dans l'intérieur des tiges sèches de la Ronce. Ann Soc Entom France. 1840;9: 1-53.

Duncan FD, Lighton JRB. Discontinuous ventilation and energetics of locomotion in the desert-dwelling female mutillid wasp, Dasymutilla gloriosa. Physiol Entomol. 1997;22, 4: 310-315.

Dutt GR. Life histories of Indian insects - IV (Hymenoptera). Memoires of the Department of Agriculture in India. 1912;12(4): 183-185.

Erlandsson S. Notes on Hymenoptera. 3. Contribution to the knowledge of the distribution of Swedish aculeate Hymenoptera. Entomol Tidskr. 1964;85: 205-217.

Evans HE, O'Neill KH. The Natural History and Behavior of North American Beewolves. Ithaca: Cornell University Press; 1988.

Evans HE. Digger Wasps as Colonizers of New Habitat (Hymenoptera: Aculeata). J NY Entomol Soc. 1974;82(4): 259-267.

Evans HE. Notes on the nesting behavior of Philanthus lepidus Cresson. Psyche. 1964;71: 142-149.

Evans HE. The comparative ethology and evolution of the sand wasps. Cambridge: Harvard University Press; 1966.

Evans HE, O’Neill KM. The sand wasps: natural history and behavior. Cambridge: Massachusetts Harvard University Press; 2007.

Evans HE, Rubink WL. Observations on the prey and nests of seven species of Cerceris (Hymenoptera: Sphecidae). Great Basin Nat. 1978;38: 59-63.

Evans HE, Matthews RW. Observations on the nesting behavior of Tachytes petiolatus (Spinola) in Colombia and Argentina (Hymenoptera: Sphecidae: Philanthini). J Kansas Entomol Soc. 1973;46, 165-175.

Evans HE, Yoshimoto CM. The ecology and nesting behavior of the Pompilidae (Hymenoptera) of the Northeastern United States. Misc Publ Entomol Soc Am. 1962;3: 66-119.

Fabricius JC. Entomologia systematica emendata et aucta, secundum classes, ordines, genera, species adjectis synonymis, locis, observationibus, descriptionibus. Hafniae: impensis Christ. Gottl. Proft; 1793. pp. 304, 366-372.

Fabricius JC. Systema Piezatorum secundum ordines, genera, species adjectis synonymis, locis, observationibus, descriptionibus. Brunsvigae 440; 1804. pp. 320, 428-439.

Fattig PW. The Mutillidae or velvet ants of Georgia. Emory Univ Mus Bull. 1943;1: 1-24.

Ferguson WE. Biological characteristics of the mutillid subgenus Photopsis Blake and their systematic values. Univ Calif Publ Entomol. 1962;27 (I): 1-91.

Ferton C. Nouvelles observations sur l'instinct des Pompilides. Actes Soc Linn Bordeaux; 1897. pp. 101-132.

Ferton C. Notes détachées, etc ..., 8e série. Ann Soc Entom France; 1911. LXXXIII, pp. 81.118, pl. III-V.

Ferton C. Notes détachées, elc ..., 9" série. Ann Soc Entom Am. France; 1920. LXXXIX, pp. 329-375.

Ferton C. Sur les moeurs des Sphecodes Latr. et des Halictus Latr. Bull Soc Entomol France. 1898; 75-78.

Field J. Patterns of provisioning and iteroparity in a solitary halictine bee, Lasioglossum (Evylaeus)fratellum (Perez), with notes on L. (E.) calceatum (Scop.) and L. (E.) villosulum (K.). Insectes Soc. 1996;43: 167-182.

Fischer RL. Observations on the nesting habits of megachilid bees. J Kansas Entomol Soc. 1951;24: 46-50.

Forsius R. Über die Verbreitung der Mutilliden, Scoliiden und Sapygiden Finnlands. Not Entomol. 1927;7: 105-111.

Friese H. Die Apidae (Blumenwespen) von Argentina nach den Reisenergebnissen der Herren A. C. Jensen-Haarup und P. Jörgensen in den Jahren 1904-1907. Silkeborg: Flora og Fauna; 1908.

Fritz MA. Mutillidae. In: Morrone JJ and Coscarón S (Eds.), Biodiversidad de artrópodos argentinos, una perspectiva biotaxonómica. Argentina: Ediciones Sur; 1998. pp. 445-451.

Gess SK, Gess FW. Pollen wasps and flowers in southern Africa. South African National Biodiversity Institute; 2010. pag. 15.

Giner Marì J. Hymenopteros de Espana. Instituto español de entomología; 1943. pag. 25.

Giovanetti M, Asís JD, Tormos J. Living in aggregations: Theories and facts in the life of Hymenoptera. Marie Curie Fellowship Association Abstracts. 2006;4: 1-5.

Gogala A. Initiation of new nests in a social bee, Halictus scabiosae Rossi (Hymenoptera: Halictidae). Opusc Zool Flumin. 1991;67: 1-7.

Gogala A. Ose mravljarice Slovenije (Hymenoptera: Mutillidae) / Velvet Ants of Slovenia (Hymenoptera: Mutillidae). Scopolia. 2017;90: 1-39.

González-Vaquero RA, Polidori C, Nieves-Aldrey JL. Taxonomy and ecology of a new species of Corynura (Hymenoptera: Halictidae: Augochlorini) from Chile and Argentina. Zootaxa. 2017;4221(1): 095-110.

Grandi G. Studi di un entomologo sugli Imenotteri superiori. Boll Ist Entomol “Guido Grandi“ Univ Studi Bologna. 1961;25:i-xvi, 1-659.

Hayes WP. (1924) Two mutillids Hyperparasitic on White Grubs. Bull Brooklyn Entomol Soc. 1924;19: 153.

Hicks CH. Pseudomasaris edwardsii Cresson, anotherpollen-provisioning wasp, with further notes on P. vespoides (Cresson). Can Entomol. 1929;61: 121-125.

Hoffer A. F VI – Mutillidae Latreille, (Kodulky). In: Prodromus Hymenopterorum Cˇecho-Slovakiae (L. Bat’a, Ed.), Pars II (in Czech). Sborník Entomologického Oddeˇlení Národního Musea v Praze. 1938;16: 177-195.

Hoffer E. Zur Biologie der Mutilla europaea L. Zool Jahrb Abt Anat Ontog Thiere. 1886;1: 677-686.

Hook AW. Prey and Parasites of Cerceris fumipennis (Hymenoptera: Sphecidae) from Central Texas, with Description of the Larva of Dasymutilla scaevola (Hymenoptera: Mutillidae). J Kansas Entomol Soc. 1991;64(3): 257-264.

Hook AW. Nesting Behavior of Chlorion cyaneum (Hymenoptera: Sphecidae), a Predator of Cockroaches (Blattaria: Polyphagidae). J Kansas Entomol Soc. 2004;77(4): 558-564.

Houston TF, Thorp RW. Bionomics of the bee Stenotritus greavesi and ethological characteristics of Stenotritidae. Rec West Aust Mus. 1984;11: 375-385.

Hurd PD Jr. (1979) Superfamily Apoidea. In: Krombein KV, Hurd JPD, Smith DR, Burks BD, editors. Catalog of Hymenoptera in America North of Mexico. Vol. 2. Washington: Smithsonian Institution Press; 1979. pp. 1741-2209.

Ihering R. Biologia das abelhas solitarias do Brazil. Rev Mus Paul. 1904;6: 461-481.

Invrea S. Mutillidae – Myrmosidae. Fauna d’Italia. 1964;5: 1-312.

Invrea F. Studi sugli Apterogynidi e Mutillidi della Palestina (Hymenoptera). Mem Soc Entomol Ital. 1965;44, 53-93.

Invrea F. Smicromyrme partita (Kl.) parassita di Philanthus triangulum F. (Hymen. Mutillidae et Sphecidae). Boll Soc Entomol Ital. 1941;73: 115-117.

Jordan R. Die Spinnenameise, Mutilla europaea, ein Bienenschädling! Deut Imker. 1935;48: 421-427.

Jörgensen P. Revision der Apiden der Provinz Mendoza, Republica Argentina (Hym.). Zool Jahrb Abt Sys Geogr Biol Tiere. 1912a;32: 89-162, 643-644.

Jörgensen P. Los crisídidos y los himenópteros aculeatos de la Provincia de Mendoza. An Mus Nac Hist Nat Buenos Aires. 1912b;22: 267-338.

Okayasu J. Velvet ants of the tribe Smicromyrmini Bischoff (Hymenoptera: Mutillidae) of Japan. Zootaxa. 2020;4723.

Katayama E. Observations on the nests of Bombus ardens Smith and B. diversus Smith (Hym., Apidae) (in Japanese). Insekuto (Utsunomiya). 1966;17(2-3): 1-6.

Katayama E, Takamizawa K, Ochiai H. Supplementary notes on the nests of some Japanese bumblebees: III. Bombus (Thoracobombus) deuteronymus maruhanbachi. Jpn J Ent. 1993;61: 749-761.

Kieffer J. Zur Lebensweise von Oxybelus uniglumis Dahlbom und ihre Parasiten Ofutilliden und Musciden. Allg Z Ent. 1902;7: 81.8.

Knerer G, Schwarz M. Beobachtungen an australischen Furchenbienen (Hymenoptera; Halictinae). Zool Anz Jena. 1978;200: 321-333.

Knerer G, Plateaux-Quénu C. (1970) The life cycle and social level of Evylaeus nigripes (Hymenoptera: Halictinae), a Mediterranean halictine bee. Can Entomol. 1970;102: 185-196.

Knerer G, Atwood CE. Parasitization of social halictine bees in southern Ontario. Proc Entomol Soc Ont. 1967;97:103-110.

Knerer G. Periodizität und Strategie der Schmarotzer einer sozialen Schmalbiene, Evylaeus malachurus (K.) (Apoidea: Halictidae). Zool Anz Leipzig. 1973;190: 41-63.

Krombein KV. Catalog of hymenoptera in America north of Mexico. Washington: Smithsonian Institution Press; 1979.

Krombein KV. Trap-nesting wasps and bees: life histories, nests and associates. Washington: Smithsonian Institution Press; 1967.

Krombein KV. Superfamily Scolioidea [pp. 1253-1321]. In: Krombein KV, Hurd PD Jr, Smith DR, Burks BD, editors. Catalog of Hymenoptera in America North of Mexico, Vol. 2: Apocrita (Aculeata). Washington: Smithsonian Institution Press; 1979. xvi + pp. 1199-2209.

Krombein KV. Biological notes on some Floridian wasps (Hymenoptera, Aculeata). American Museum novitates, Archbold Biological Station no. 2201; 1964.

Krombein KV. Miscellaneous prey records of solitary wasps. III. Proc Biol Soc Wash. 1958;71: 21-26.

Krombein KV. (1959) Biological notes on some ground-nesting wasps at Kill Devil Hills, North Carolina, 1958, and additions to the faunal list (Hymenoptera, Aculeata). Proc Entomol Soc Wash. 1959;61: 193-199.

Krombein KV. Biosystematic studies of Ceylonese wasps, III. Life history, nest and associates of Paraleptomenes mephitis (Cameron) (Hymenoptera, Eumenidae). J Kansas Entomol Soc. 1978;51(4): 721-734.

Krombein KV, Norden BB. Behavior of nesting Episyron conterminus posterus (Fox) and its cleptoparasite Ephuta s. slossonae (Fox) (Hymenoptera: Pompilidae, Mutillidae). Proc Entomol Soc Wash. 1996;98: 188-194.

Krombein KV. Host relationships, ethology and systematics of Pseudomethoca Ashmead (Hymenoptera: Mutillidae, Andrenidae, Halictidae and Anthophoridae). Proc Entomol Soc Wash. 1992;94: 91-106.

Krombein KV, Lelej AS. Biosystematic studies of Ceylonese wasps, XXII: Bethsmyrmilla, a new genus of mutillid wasps (Hymenoptera: Mutillidae: Myrmillinae). Proc Entomol Soc Wash. 1999;101(1): 143-148.

Krombein KV, Norden BB, Rickson MM, Rickson FR. Biodiversity of the Domatia Occupants (Ants, Wasps, Bees, and Others) of the Sri Lankan Myrmecophyte Humboldtia laurifolia Vahl (Fabaceae). Smithson Contr Zool. 1999;603: I-IV + 1-34.

Lelej AS, Fateryga AV, Ivanov SP. The velvet ants (Hymenoptera: Mutillidae) of the Crimean Peninsula. Far East Entomol. 2016;314: 1-24.

Lelej AS, Choi JY, Tripotin P. Review of the mutillid wasps (Hymenoptera, Mutillidae) of Korea. Far East Entomol. 2001;96: 1-15.

Lelej AS. The velvet ants (Hymenoptera, Mutillidae) of the USSR and neighboring countries. Nauka Leningrad. 1985;268.

Lelej AS. A review of Palaearctic and Oriental species of genus Nemka Lelej with description of Oriental genus Mickelomyrme gen. n. (Hymenoptera, Mutillidae). Far East Entomol. 1995;6: 1-20.

Lelej AS. Catalogue of the Mutillidae (Hymenoptera) of the Oriental region. Dalnauka Vladivostok; 2005.

Lenko K. Hoplomutilla triumphans Mickel, 1939 (Hymenoptera, Mutillidae) como parasito de abelhas do gênero Euplusia (Hymenoptera, Apoidea). Pap Avulsos Dep Zool Sec Agric. 1964;16: 199-205.

Lichtenstein J. Feuille des jeunes naturalistes, Remarques sur Mutilla europaea. VIII; 1878. p. 35.

Linsley EG, MacSwain JW. Some observations on the nesting habits and prey of Cerceris californica Cresson (Hymenoptera: Sphecidae). Ann Entomol Soc Am. 1956;49: 71-84.

Lopez V, Bartholomay PR, Lima FVO, Silvestre R. Male description of Horcomutilla projectifrons (Cresson, 1902) Hymenoptera, Mutillidae) and the first host record for the genus. Zootaxa. 2019;4559(3): 573.

Lopez AW. Annual report of the Entomological Research Bureau, Philippine Sugar Association 1930-1931. In Annual Report of the Research Bureau, Philippine Sugar Association; 1931. pp. 227-273.

Luz DR, Waldren GC, Melo GAR. Bees as hosts of mutillid wasps in the Neotropical region (Hymenoptera, Apidae, Mutillidae). Rev Bras Entomol. 2016;60: 302-307.

Macgown JA. New state records of Myrmosinae (Hymenoptera: Mutillidae) from Mississippi, U.S.A. with notes on interactions between Myrmosula parvula (Hymenoptera: Myrmosinae) and Lasioglossum imitatum (Hymenoptera: Halictidae). Entomol News. 2006;117: 313-318.

Manley DG, Deynip MA. Notes on the biology of Dasymutilla pyrrhus (Fox) (Hymenoptera: Mutillidae). J Entomol Sci. 1989;24:(1) 53-56.

Manley DG, Carithers TP. (1998) A new host record for Sphaeropthalma pensylvanivca pensylvanica (Hymenoptera: Mutillidae). Entomol News. 1998;109: 198-200.

Manley DG. Dasymutilla jalisco, a new species of velvet ant (Hymenoptera: Mutillidae) plus synonymy for Dasymutilla canina (Smith). Proc Entomol Soc Wash. 2003;105: 679-684.

Matthews RW, Evans HE. Biological Notes on Two Species of Sericophorus from Australia (Hymenoptera: Sphecidae). Psyche. 1970;77: 413-429.

May J. Cˇmeláci v CˇSR: jejich bionomie, chov a hospodárˇsk´y v´yznam. Prague: Cˇeskoslovenská Akademie Zemeˇdeˇlsky´ch Veˇd; 1959.

Melander A l. and c. t. brues. (1903) Guests and parasites of the burrowing bee Halictus. Biol Bull. 1903;5(1): 1-27.

Michener CD. The Bionomics of a Primitively Social Bee, Lasioglossum versatum (Hymenoptera: Halictidae) J Kansas Entomol Soc. 1966;39(2): 193-217.

Michener CD, Wille A. The bionomics of a primitively social bee, Lasioglossum inconspicuum. Univ Kans Sci Bull. 1961;42: 1123-1202.

Mickel CE. Biological and taxonomic investigations on the mutillid wasps. Bull US Natl Mus. 1928;143.

Mickel CE. Pseudomethoca willei N. Sp. Reared from Cells of the Bee Lasioglossum (Dialictus) umbripenne (Ellis) (Hymenoptera: Mutillidae; Apoidea) J Kansas Entomol Soc. 1969;42(4): 524-526.

Mickel CE. A revision of the mutillid wasps of the genera Myrmilloides and Pseudomethoca occurring in America north of Mexico. Proc US Natl Mus. 1924a;64: 1-51.

Mickel CE. An analysis of a bimodal variation in size of the parasite Dasymutilla bioculata (Cresson). Entomol News. 1924b;35: 236-242.

Mickel CE. A synopsis of the neotropical mutillid genus Euspinolia Ashmead (Hym.). Rev Entomol Rio J. 1938;9: 53-74.

Miyamoto S. On the nest of Bombus diversus Smith which collapsed before completion (Biological studies on Japanese bees XI). Akitu. 1959;8: 85-90.

Miyamoto S. Biology of Bombus ignitus Smith. Kontyû. 1963;31: 91-98.

Móczár L. Das Nest von Bombus alticola Kriechbaumer (Hymenoptera: Apidae). Acta Biol Szeged. 1977;23: 133-138.

Monfared A, Shahrza A. Mutilla saltensis (Mutillidae, Hymenoptera) a parasite of Bombus armeniacus colony in Iran. IXth European Congress of Entomology Budapest, Hungary; 2010.

Morato EF. Xystromutilla asperiventris André, 1905 (Mutillidae) reared from sphecid wasps in trap-nests, Manaus, Amazonas, Brazil. Sphecos. 1994;28: 13-14.

Morimoto R, Iwata K, Yasumatsu K. Observations on Bombus diversus Smith (Hymenoptera, Apidae). Mushi. 1951;22: 51-58.

Moure JS. A review of the genus Paroxystoglossa (Hymenoptera: Halictidae). Univ Kans Sci Bull. 1960;40: 121-233.

Ordway E. Sphecodes pimpinellae and other enemies of Augochlorella (Hymenoptera: Halictidae). J Kansas Entomol Soc. 1964;37: 139-152.

Pagden HT. Description and records of Austro-Malaysian Methocidae and Mutillidae (Hymenoptera). Trans R Entomol Soc Lond. 1949;100(8): 191-231.

Parker FD. Two hosts of Lomachaeta varicrjata Mickel (Hymenoptera: Mutillidae). Pan-Pac Entomol. 1962;38(2): 116.

Parker FD, Bohart RM. Host-parasite associations in some twig-nesting Hymenoptera from Western North America. Ibid. 1966;42(2).

Parks R. A Cactus Bee Diadasia rinconis Female (Hymenoptera: Apidae) Aggressively Defends a Nest Against the Velvet Ant Pseudomethoca propinqua (Hymenoptera: Mutillidae). Entomol News. 2018;127(4): 329-332.

Peringuey L. Description of some New or Little Known South African Mutillidae in the Collection of the South African Museum. Ann S Afr Mus. 1898;1: 33-94.

Pitts JP, Parker F, Pitts-Singer TL. A Review of the Sphaeropthalma uro Species-group (Hymenoptera: Mutillidae), with Taxonomic Changes. J Kansas Entomol Soc. 2004;77(3):222-234.

Pitts JP, Tanner D, Waldren GC, Parker FD. Facultative Size-Dependent Sex Allocation in Sphaeropthalma pensylvanica Lepeletier (Hymenoptera: Mutillidae) with Further Host Records. J Kansas Entomol Soc. 2010;83(1): 68-75.

Pitts PP, Parker FD. Description of the female and larval stage of Odontophotopsis succinea Viereck (Hymenoptera: Mutillidae), with new synonymy and notes. Zootaxa. 2003;137: 1-10 DOI: 10.5281/zenodo.156584.

Pitts JP. Host Record for Dilophotopsis concolor crassa (Viereck) (Hymenoptera: Mutillidae). J Entomol Sci. 2003;38: 485-486.

Polidori C, Borruso L, Boesi R, Andrietti F. (2009) Segregation of temporal and spatial distribution between kleptoparasites and parasitoids of the eusocial sweat bee, Lasioglossum malachurum (Hymenoptera: Halictidae, Mutillidae). Entomol Sci. 2009;12: 116-129.

Polidori C, Mendiola P, Asìs JD, Tormos J, Selfa J. Temporal asynchrony and spatial co-occurrence with the host: the foraging patterns of Nemka viduata, a parasitoid of digger wasps (Hymenoptera: Mutillidae and Crabronidae). J Ethol. 2010;28: 353-361.

Polidori C, Beneitez A, Asís JD, Gayubo SF, Tormos J. Predicting Activity Patterns from Resource Exploitation in Guilds of Digger Wasps’ Natural Enemies. Adv Sci Lett. 2012;18: 77-84.

Pouvreau A. Les ennemis des bourdons. I – Étude d’une zoocé-nose: le nid de bourdons. Apidologie. 1973;4: 103-148.

Powell JA. Biology and behavior of Nearctic wasps of the genus Xylocelia, with special reference to X. occidentalis (Fox) (Hymenoptera: Sphecidae). Wassmann J Biol. 1963;21: 155-176.

Quintero A, Cambra RA. Pappognatha Mickel (Hymenoptera: Mutillidae: Sphaeropthalminae): New Species, Sex Associations, Hosts, and New Distribution Records. J Hymenopt Res. 2005;14: 191-199.

Quintero A, Cambra RA. On the Identity of Scaptopoda F. Lynch Arribálzaga, New Taxonomic Changes and New Distribution Records for Neotropical Mutillidae (Hymenoptera), with Notes on Their Biology. T Am Entomol Soc. 2001;127(3): 291-304.

Quintero D, Cambra RA. Timulla centroamericana (Dalla Torre) (Hymenoptera: Mutillidae), a parasitoid of Liris (Hymenoptera: Sphecidae). Southwest Entomol. 1996;21, 205-207.

Radoszkowski O. (1885) Revision des armures copulatrices des mâles de la famille Mutillides. Horae Soc Ent Ross. 1885;19: 3-49, pl. I–IX.

Rau P. Field studies in the behavior of non-social wasps. St Louis Acad Sci Trans. 1928;25: 319-489.

Rau P, Rau N. The biology of the mud-daubing wasps as revealed by the contents of their nests. J Anim Behav. 1916;6: 27-63.

Rayment T. Biology of a new halictine bee and specific descriptions of its parasites. Arb über Physiol u Angew Ent. 1937;4: 30-60.

Rayment T. Bees of the Portland District. PFNC. 1953; iii + 39 pp.

Rayment T. Taxonomy, morphology and biology of sericophorine wasps with diagnoses of two new genera and descriptions of forty new species and six sub-species. Mem Mus Vic. 1955a;19: 11-105. <https://doi.org/10.24199/j.mmv.1955.19.02>.

Rayment T. Dimorphism and parthenogenesis in halictine bees. Aust Zool. 1955b;12: 142-153, pls. 20-22.

Rayment T. The Nomia australica Sm. complex, its taxonomy, morphology and biology, with the description of a new mutillid wasp. Aust Zool. 1956;12: 176–200, pls. 23–26.

Rocha-Filho LC, Melo GAR. Nesting biology and behavioural ecology of the solitary bee Monoeca haemorrhoidalis (Smith) and its cleptoparasite Protosiris gigas Melo (Hymenoptera: Apidae: Tapinotaspidini; Osirini). J Nat Hist. 2011;45: 2815-2840.

Rocha-Filho LC, Silva CI, Gaglianone MC, Augusto SC. Nesting behavior and natural enemies of Epicharis (Epicharis) bicolor Smith, 1854 (Hymenoptera Apidae). Trop Zool. 2008;21: 227-242.

Roubik DW. A Mixed Colony of Eulaema (Hymenoptera: Apidae), Natural Enemies, and Limits to Sociality. J Kansas Entomol Soc. 1990;63(1): 150-157.

Roubik DW. Ecology and Natural History of Tropical Bees. Cambridge: Cambridge University Press; 1989.

Rozen JG Jr. The Biology of Svastra obliqua obliqua (Say) with a Taxonomic Description of its Larvae (Apoidea, Anthophoridae). Am Mus Novit. 1964;2170: 1-13.

Rozen JG. Life history and immature stages of the bee Neofidelia (Hymenoptera, Fideliidae). Am Mus Novit. 1973;2519: 1-14.

Rucker A. A Glimpse of the Life History of Mutilla vesta Cresson. Entomol News. 1903;14: 75-77.

Sakagami SF, Katayama E. Nests of Some Japanese Bumblebees (Hymenoptera, Apidae) (With 21 Text-figures and 17 Tables) J Fac Sci Hokkaido Univ, Ser VI Zool. 1977;21(1): 92-153.

Sanchez CS, Genaro JA. Notas Sobre la Conducta de Nidificacion de Philanthus banabacoa Alayo (Hymenoptera: Sphecidae). Caribb J Sci. 1992;28(1-2): 44-46.

Saz E. La Mutilla europaea y los Bombus de Nuria. Boll Soc Entomol Esp. 1935;17: 85-98.

Schmidt JO, Buchmann SL. Floral biology of the saguaro (Cereus giganteus). Oecologia. 1986;69: 491-498.

Schuster RM. A Revision of the Genus Ephuta (Mutillidae) in America North of Mexico. Journ NY Entomol Soc. 1956;64: 7-84.

Shappirio DG. Observations on the biology of some mutillid wasps (Hym.: Mutillidae)? II, with new distributional records. Bull Brooklyn Entomol Soc. 1949;43: 157-158.

Sichel J, Radoszkowksy OI. Essai d'une Monographie de Mutilles de l'ancient continent. Horoe Soc Entom Ross. 1869;6.

Sichel J. Sur la rareté relative de certains Hyménoptères et notamment sur la Mutilla incompleta et la Crocisas Culltellaris. Ann Soc Entomol France. 1852;10: 561-567.

Skorikov AS. Zur Mutilliden-Fauna Zentralasiens (in Russian with German summary). Trudy Tadzhikskoi Bazy, Akademiya Nauk SSSR. 1935;5: 257-349.

Smith F. Catalogue of Hymenopterous Insects in the Collection of the British Museum. Part III. Mutillidae and Pompilidae. London: Taylor and Francis; 1855.

Strand E. Beitrag zur Bienenfauna von Paraguay. (Hym.). Deutsch Entomol Z. 1909; 227-237.

Su W, Liang C, Ding G, Jiang Y, Huang J, Wu J. First Record of the Velvet Ant Mutilla europaea (Hymenoptera: Mutillidae) Parasitizing the Bumblebee Bombus breviceps (Hymenoptera: Apidae). Insects. 2019;12, 10(4).

Taniguchi S. Biological studies on the Japanese bees. II. Study on the nesting behaviour of Bombus ardens Smith. Science Rep Hyogo Univ Agric. 1955;2: 89-96.

Taylor CK, Murphy MV, Hitchen Y, Brothers DJ. Four new species of Australian velvet ants (Hymenoptera: Mutillidae, Aglaotilla) reared from bee and wasp nests, with a review of Australian mutillid host records. Zootaxa. 2019;4609(2): 201.

Templado J. Smicromyrme viduata Pall. parasito de Bembix mediterranea. Handl Graellsia. 1958;16(1/3): 45-47.

Thompson WR. A Catalogue of Parasites and Predators of Insect Pests Section 2: Host Parasite Catalogue Part 5: hosts of the Hymenoptera (Miscgasteridae to Trigonalidae) Lepidoptera and Strepsiptera. Ottawa: CAB; 1972.

Thompson WR. A catalogue of the parasites and predators of Insect pests. Sec. 1. Parasitic host catalogue Pt. 4. Belleville: Imperial Parasite Service; 1944.

Torchio PF, Trostle GE. Biological Notes on Anthophora urbana urbana and Its Parasite, Xeromelecta californica (Hymenoptera: Anthophoridae), Including Descriptions of Late Embryogenesis and Hatching. Ann Entomol Soc Am. 1986;79: 434-447.

Tormos J, Asìs JD, Benéitez A, Gayubo SF. Description of the mature larva of the sand wasp Bembix bidentata and those of its parasitoids (Hymenoptera: Crabronidae, Chrysididae, Mutillidae). Fla Entomol. 2009;92: 43-53.

Tournier H. Hyménoptères. Descriptions d'espèces nouvelles & remarques diverses. L'Entomologiste Genevois. 1889;1: 13.

Tsuneki K. Studies on the Mutillidae of Japan (Hymenoptera). Etizenia. 1972;61: 1-26.

Uboni A, Lorenzi MC. Poor Odors, Strength, and Persistence Give Their Rewards to Mutilla europaea Visiting Dangerous Wasp Nests. J Insect Behav. 2013;26: 246-252.

Vinson SB, Frankie GW, Coville RE. Nesting Habits of Centris flavofasciata Friese (Hymenoptera: Apoidea: Anthophoridae) in Costa Rica. J Kansas Entomol Soc. 1987;60(2): 249-263.

Weber NA. A new american myrmosid (Hymenoptera, Myrmosidae). Psyche. 1934;41: 57-59.

Wheeler GC. A mutillid mimic of an ant (Hymenoptera: Mutillidae and Formicidae). Entomol News. 1983;94: 143-144.

Williams FX. Philippine Wasp Studies. Part 11. Descriptions of New Species and Life History Studies. Bull Hawaiian Sug Plrs’ Ass Ex Stn, Ent Ser. 1919;14: 19-180.

Williams KA, Pitts JP. Caribbean and Mexican Additions to the Dasymutilla bioculata Species-Group (Hymenoptera: Mutillidae). Ann Entomol Soc Am. 2013;106(4): 429-436.

Zanette LRS, Soares LA, Pimenta HC, Conçalves AM, Martins RP. Nesting biology and sex ratios of Auplopus militaris (Lynch-Arribálzaga 1873) (Hymenoptera Pompilidae). Trop Zool. 2004;17: 145-154.

Zillikens A, Steiner J. Nest sites, breeding cycle and possible cleptoparasites of Monoeca catarina Aguiar (Hymenoptera, Apidae, Tapinotaspidini) from Santa Catarina Island, southern Brazil. Stud Neotrop Fauna E. 2020;55(1): 1-7.
